# Total synthesis of the capsular polysaccharide repeating unit towards the development of a glycoconjugate vaccine against *Klebsiella pneumoniae* ST512

**DOI:** 10.3762/bjoc.22.64

**Published:** 2026-05-29

**Authors:** Shuo Zhang, Ondřej Daněk, Peter H Seeberger

**Affiliations:** 1 Department of Biomolecular Systems, Max Planck Institute of Colloids and Interfaces, Am Mühlenberg 1, 14476 Potsdam, Germany; 2 Institute of Chemistry and Biochemistry, Freie Universität Berlin, Arnimallee 22, 14195 Berlin, Germany; 3 Current Address: Department of Chemistry, Dalian University of Technology, No. 2 Linggong Road, 116024 Dalian, China; 4 Institute of Organic Chemistry and Biochemistry of the Czech Academy of Sciences, Flemingovo náměstí 542/2, 160 00 Praha 6, Czech Republic

**Keywords:** glycoconjugate vaccines, glycosylation, oligosaccharides, selectivity control, total synthesis

## Abstract

*Klebsiella pneumoniae* ST512 is an emerging multidrug-resistant pathogen whose capsular polysaccharide represents a prime target for vaccine development. Here, we report the first total synthesis of the branched hexasaccharide repeating unit of the *K. pneumoniae* ST512 CPS, together with four structurally related oligosaccharide analogues. Key synthetic challenges including the stereoselective construction of the 1,2-*cis* glycosidic linkage on the galacturonic acid core and the inherently low reactivity of elongated oligosaccharide intermediates were addressed employing orthogonally protected building blocks. The resulting library of conjugation-ready oligosaccharides, equipped with aminopentyl linkers, enables glycan microarray-based identification of minimal immunogenic epitopes. This work establishes a robust chemical foundation for the rational development of semi-synthetic glycoconjugate vaccines targeting *K. pneumoniae* ST512.

## Introduction

The emergence and rapid spread of multidrug-resistant bacteria represent a critical threat to global health [1–4]. Among these pathogens, *Klebsiella pneumoniae* has surfaced as a leading cause of nosocomial infections, characterized by high morbidity and an escalating degree of antimicrobial resistance [5–8]. This bacterium is frequently responsible for severe clinical conditions, including urinary tract infections, pneumonia, and septicemia [9–10]. The increasing isolation of hypervirulent strains has severely limited available therapeutic options, underscoring the urgent need for alternative prophylactic strategies. Particularly, the *K. pneumoniae* sequence type 512 (ST512), a dominant carbapenem-resistant clone closely related to the notorious ST258, has become a major focus of clinical concern due to its widespread dissemination and limited treatment options [6,11–13].

The outer membrane of *K. pneumoniae* is encapsulated by high-molecular-weight capsular polysaccharides (CPS). These polysaccharides form a protective layer that facilitates the evasion of host immune defenses and enhances tolerance to antibiotic treatment [14–16]. As a major virulence factor, CPS can trigger specific adaptive immune responses, rendering these glycans attractive targets for vaccine development [17–18]. Glycoconjugate vaccines that are constructed by covalently attaching glycan antigens to carrier proteins, have emerged as highly effective vaccine candidates for preventing bacterial colonization and the resulting infectious diseases [19–23].

While several studies have explored vaccine candidates against *K. pneumoniae*, no commercial vaccines are currently available [24–27]. The identification of immunogenic epitopes is a key step toward the development of efficacious semi-synthetic vaccines [19,28]. Herein, we report the first total synthesis of a series of conjugation-ready oligosaccharides related to the CPS repeating unit of *K. pneumoniae* ST512. These structurally defined synthetic glycans serve as a precise molecular platform for elucidating structure-immunogenicity relationships and guiding the rational design of glycoconjugate vaccines.

## Results and Discussion

**Figure 1:**
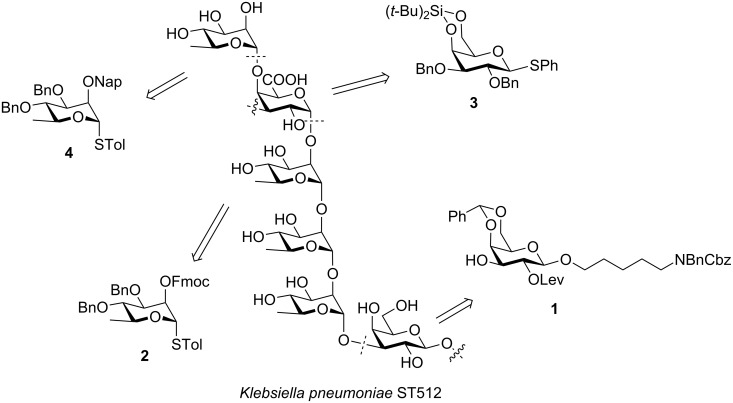
Structure of the *K. pneumoniae* ST512 CPS repeating unit and retrosynthetic analysis.

The CPS of *K. pneumoniae* ST512 consists of a branched hexasaccharide repeating unit {3)-[α-ʟ-Rha*p*-(1→4)]-α-ᴅ-Gal*p*A-(1→2)-α-ʟ-Rha*p*-(1→2)-α-ʟ-Rha*p*-(1→2)-α-ʟ-Rha*p*-(1→3)-β-ᴅ-Gal*p*-(1→}*_n_* (Figure 1) [13]. The chemical synthesis of this molecule is challenging due to the stereoselective formation of the 1,2-*cis* glycosidic linkage on the galacturonic acid core. In addition, the assembly and subsequent functionalization of long oligosaccharide chains are hampered by their intrinsically low reactivity [29]. Overcoming these challenges relying on a linear strategy employing four key building blocks **1**–**4**, we describe the first chemical synthesis of the hexasaccharide repeating unit.

**Scheme 1:**
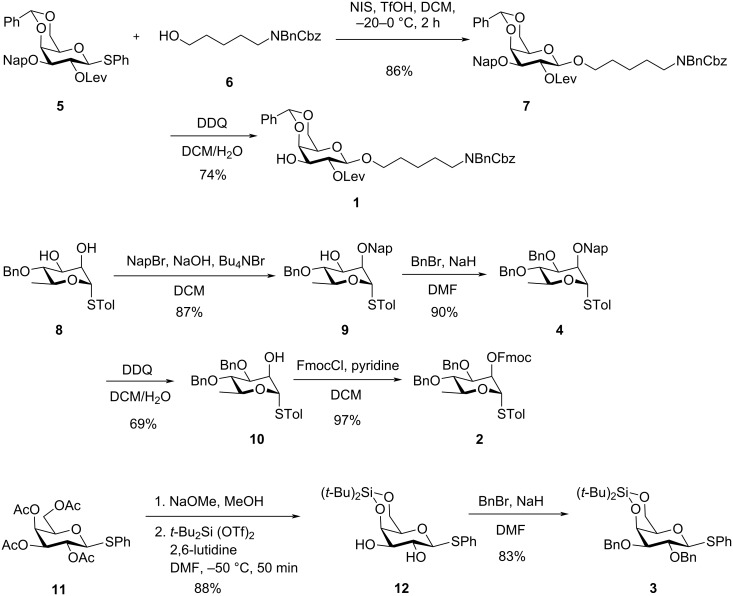
Preparation of building blocks **1**–**4**.

The total synthesis commenced with the preparation of the orthogonally protected monosaccharide building blocks **1–4** (Scheme 1). Galactoside **1** was synthesized starting from the β-selective glycosylation of thioglycoside **5** [30] with aminopentyl linker **6**. This reaction was promoted by *N*-iodosuccinimide (NIS) and trifluoromethanesulfonic acid (TfOH) to afford **7** in 86% yield, neighboring participation effect of the levulinoyl (Lev) group at C2 position provided only the β-product. The linker was introduced in anticipation of conjugation to a carrier protein or a glycan microarray surface [31]. Subsequent removal of the 2-naphthylmethyl (Nap) group using 2,3-dichloro-5,6-dicyano-1,4-benzoquinone (DDQ) furnished the desired alcohol **1** in 74% yield.

For the synthesis of ʟ-rhamnoside building blocks **2** and **4**, the C2-hydroxy group of known thioglycoside **8** [32–33] was regioselectively protected with a Nap group to yield **9** (87% yield). Protection of the remaining C3-hydroxy group with a benzyl (Bn) ether furnished building block **4**. To obtain building block **2**, the Nap group was replaced with a fluorenylmethyloxycarbonyl protecting group (Fmoc) via a two-step sequence: DDQ-mediated deprotection (69% yield) followed by treatment with 9-fluorenylmethyloxycarbonyl chloride to install the Fmoc group in 97% yield.

The galacturonic acid precursor **3** was prepared from thioglycoside **11**. Global deacetylation followed by the introduction of a 4,6-*O*-silylidene group afforded diol **12** (88% over two steps). Finally, protection of the hydroxy groups with benzyl ethers furnished building block **3** in 83% yield.

**Scheme 2:**
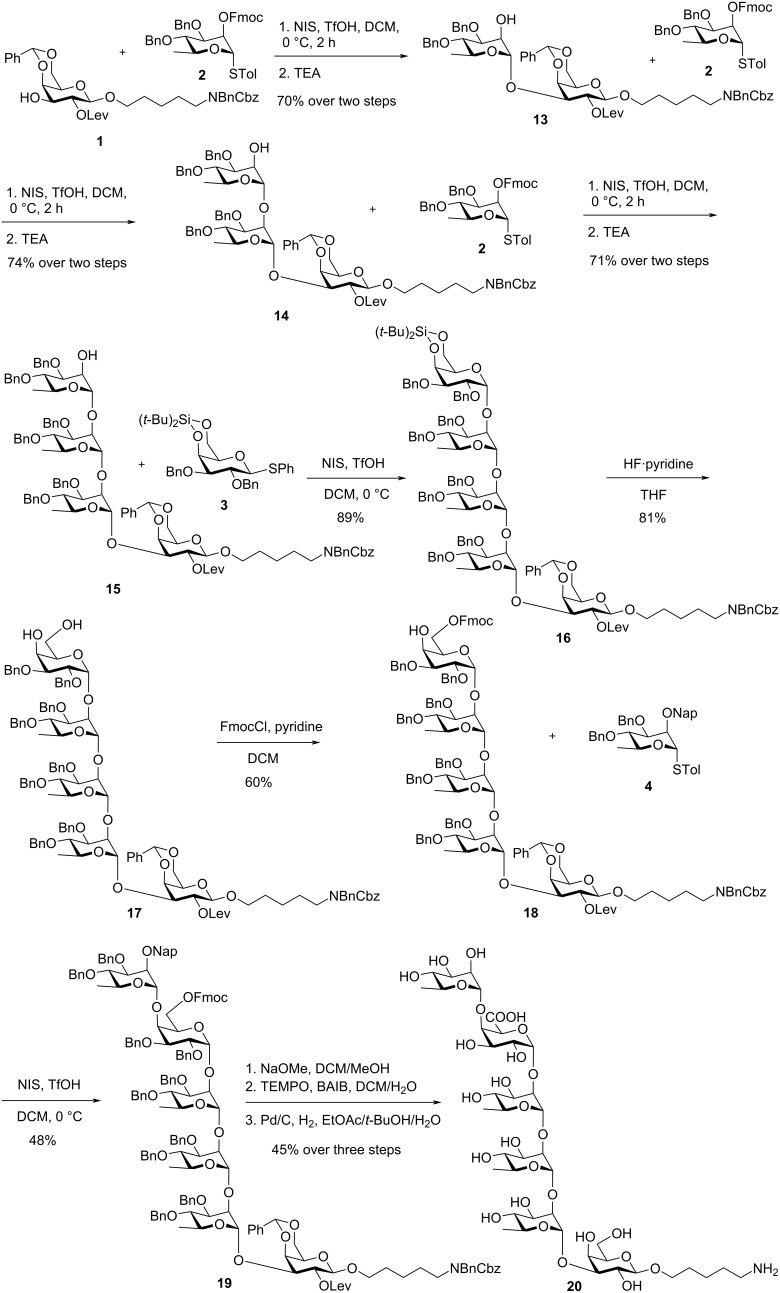
Synthesis of hexasaccharide repeating unit **20**.

With the building blocks in hand, the assembly of the hexasaccharide was pursued as outlined in Scheme 2. The synthesis commenced with the coupling of monosaccharides **1** and **2** promoted by NIS/TfOH followed by a one-pot Fmoc deprotection, afforded disaccharide **13** in 70% yield over two steps. Complete α-selectivity was observed, which can be attributed to the anomeric effect and the neighboring group participation of the C2-Fmoc group [34]. Two sequential cycles of glycosylation and Fmoc removal furnished tetrasaccharide **15**. Subsequently, donor **3** was coupled with compound **15** under NIS/TfOH promotion to yield pentasaccharide **16** in 89% yield. The stereoselective construction of the 1,2-*cis* linkage was directed by the bulky 4,6-*O*-silylidene protecting group of **3**. Treatment with HF·pyridine successfully cleaved the silylidene group, producing pentasaccharide diol **17** in 81% yield.

The final stages involved the regioselective installation of an Fmoc group at the C6-hydroxy group, followed by glycosylation with building block **4** to provide hexasaccharide **19** with complete α-selectivity. Global deprotection was achieved by removing the Fmoc and Lev protecting groups with sodium methoxide, followed by 2,2,6,6-tetramethyl-1-piperidinyl-1-oxyl (TEMPO)/bis(acetoxy)iodobenzene (BAIB)-mediated oxidation of the C6-primary alcohol [35]. Final hydrogenolysis furnished the target hexasaccharide repeating unit **20** in 45% yield over three steps.

**Scheme 3:**
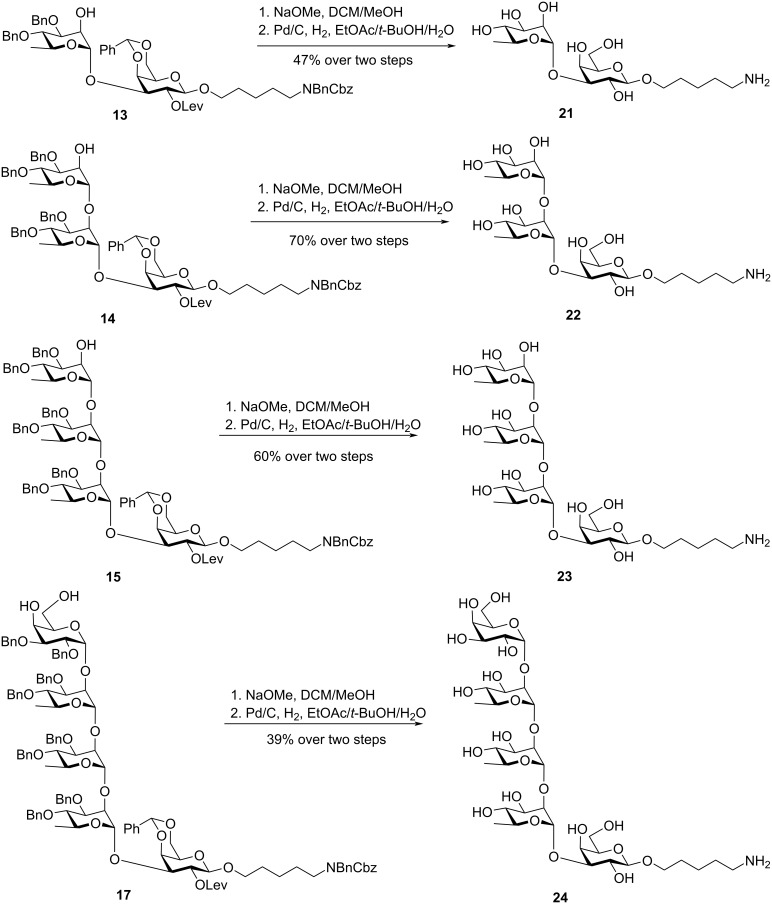
Synthesis of the analogues **21**–**24**.

The identification of the minimal antigenic epitope is of paramount importance in the development of semi-synthetic glycoconjugate vaccines [36–37]. To expand the library of synthetic carbohydrate antigens and provide a chemical foundation for subsequent high-throughput screening, a series of analogues including di-, tri-, tetra-, and pentasaccharides were synthesized (Scheme 3). We utilized the intermediate products from the assembly of the hexasaccharide repeating unit **20**. The protecting groups of disaccharide **13** were removed by treatment with sodium methoxide followed by catalytic hydrogenation over palladium on carbon, affording disaccharide **21** in 47% yield over two steps. Similarly, trisaccharide **22**, tetrasaccharide **23**, and pentasaccharide **24** were prepared from intermediates **14**, **15**, and **17**, respectively. The analogues **21–24**, together with the repeating unit **20**, constitute a collection of well-defined synthetic oligosaccharide antigens resembling the surface of *K. pneumoniae* ST512. Glycan microarray analysis is currently underway to identify the lead antigens and key epitopes responsible for inducing specific immune responses against the native ST512 CPS.

## Conclusion

We accomplished the first total synthesis of the hexasaccharide repeating unit of the *K. pneumoniae* ST512 capsular polysaccharide, along with four structurally defined analogues. Major synthetic challenges, including the stereoselective formation of multiple 1,2-*cis* and 1,2-*trans* glycosidic linkages and the selective manipulation of long oligosaccharide chains, were successfully overcome using orthogonally protected building blocks. The resulting conjugation-ready oligosaccharides bearing aminopentyl linkers provide direct access to glycan microarray screening and in vivo immunological evaluation. This work represents a key step toward the development of semi-synthetic glycoconjugate vaccines against multidrug-resistant *K. pneumoniae.*

## Supporting Information

File 1 Experimental procedures and NMR spectra.

## Data Availability

All data that supports the findings of this study is available in the published article and/or the supporting information of this article.
