# Supplementary material for: Total synthesis of the capsular polysaccharide repeating unit towards the development of a glycoconjugate vaccine against Klebsiella pneumoniae ST512
Source: Beilstein J Org Chem. 2026 May 29;22:821–7. doi: 10.3762/bjoc.22.64 (PMC13224054; doi:10.3762/bjoc.22.64)
Supplement: File 1 — Experimental procedures and NMR spectra. [file Beilstein_J_Org_Chem-22-821-s001.pdf]

BEILSTEIN JOURNAL OF ORGANIC CHEMISTRY

Supporting Information

for

Total synthesis of the capsular polysaccharide repeating unit towards the development of a glycoconjugate vaccine against *Klebsiella pneumoniae* ST512

Shuo Zhang, Ondřej Daněk and Peter H. Seeberger

Beilstein J. Org. Chem. **2026,** *22,* 821–827. doi:10.3762/bjoc.22.64

Experimental procedures and NMR spectra

License and Terms: This is a supporting information file under the terms of the Creative Commons Attribution License (https://creativecommons.org/licenses/by/4.0). Please note that the reuse, redistribution and reproduction in particular requires that the author(s) and source are credited and that individual graphics may be subject to special legal provisions.
The license is subject to the *Beilstein Journal of Organic Chemistry* terms and conditions: (https://www.beilstein-journals.org/bjoc/terms)

Table of contents

1. General information S3

2. Experimental procedures S3

3. NMR spectra S27

4. References S79

1. General information

Commercial grade solvents and reagents were used without further purification. Sugar building blocks indicated as commercially available were purchased from GlycoUniverse GmbH. Anhydrous solvents were obtained from a solvent drying system (JCMeyer) or dried according to reported procedures. Analytical TLC was performed on Kieselgel 60 F254 glass (Macherey-Nagel). Spots were visualized with UV light, sulfuric acid stain [1 mL of 3-methoxyphenol in 1 L of EtOH and 30 mL H_2SO_4] or ceric ammonium molybdate stain [0.5 g $Ce(NH_4)_4(SO_4)_4 \cdot 2H_2O$, 12 g $(NH_4)_6Mo_7O_{24} \cdot 4H_2O$ and 15 mL H_2SO_4 in 235 mL H_2O]. Flash chromatography was performed on Kieselgel 60 230-400 mesh (Sigma-Aldrich). Preparative, high-performance liquid chromatography was performed on an Agilent 1260 Infinity instrument with an attached DAD detector with automated fraction collection at 210, 230 and 254 nm wavelengths using a Thermo Scientific™ Hypercarb™ column (150 × 20 mm, 5 µm, 35005-159270A). NMR spectra were recorded on a Varian 400 MHz spectrometer (Agilent), Ascend 400 MHz (cryoprobe, Bruker), Varian 600 MHz (Agilent) and Ascend 700 MHz (cryoprobe, Bruker) at 25 °C unless indicated otherwise. Chemical shifts (δ) are reported in parts per million (ppm) relative to the respective residual solvent peaks ($CHCl_3$: δ 7.26 in ^1H and 77.16 in ^{13}C; HDO δ 4.79 in ^1H). Bidimensional and non-decoupled experiments were performed to assign identities of peaks showing relevant structural features. The following abbreviations are used to indicate peak multiplicities: *s* (singlet), *d* (doublet) *dd* (doublet of doublets), *t* (triplet), *dt* (doublet of triplets), *td* (triplet of doublets), *q* (quartet), *p* (pentet), *m* (multiplet). Coupling constants (*J*) are reported in Hertz (Hz). NMR spectra were processed using MestreNova 14.1 (MestreLab Research). High-resolution mass spectra (ESI-HRMS) were recorded with a Xevo G2-XS Q-Tof (Waters).

2. Experimental procedures

Phenyl 4,6-*O*-benzylidene-2-*O*-levulinoyl-3-*O*-(2-naphthylmethyl)-1-thio-*β*-D-galactopyranoside (5)

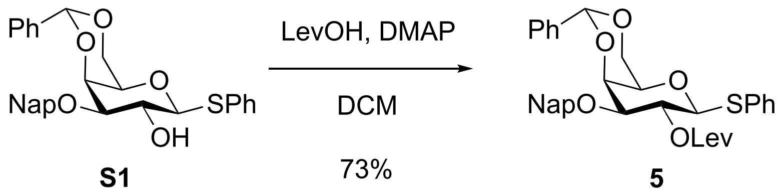

To a stirred solution of known thioglycoside **S1**[1] (2.5 g, 5.0 mmol) in DCM (25 mL) levulinic acid (0.77 mL, 7.5 mmol), EDC·HCl (1.43 g, 7.5 mmol) and DMAP (0.42 g, 3.4 mmol) were added and the reaction mixture was stirred for 18 h at room temperature. The reaction mixture was diluted with DCM (25 mL), washed with HCl (0.05M, 50 mL), sat. $NaHCO_3$ and brine. The combined organic layer was dried over Na_2SO_4, filtered and concentrated under reduced pressure. The crude product was purified by flash silica column chromatography (hexane/ethyl acetate = 1/1) to give **5** as a light yellow solid (2.18 g, 3.65 mmol, 73%)[2].

^{1}H NMR (400 MHz, $CDCl_3$) δ 7.89 – 7.77 (m, 4H, Ar), 7.65 – 7.59 (m, 2H, Ar), 7.54 – 7.47 (m, 3H, Ar), 7.47 – 7.42 (m, 2H, Ar), 7.41 – 7.35 (m, 3H, Ar), 7.33 – 7.22 (m, 3H, Ar), 5.46 (s, 1H, benzylidene, CH), 5.38 (t, *J* = 9.7 Hz, 1H, H-2), 4.83 (d, *J* = 2.7 Hz, 2H, Nap-CH_2), 4.64 (d, *J* = 9.9 Hz, 1H, H-1), 4.34 (dd, *J* = 12.4, 1.7 Hz, 1H, H-6), 4.22 – 4.20 (m, 1H, H-4), 3.98 (dd, *J* = 12.3, 1.7 Hz, 1H, H-6), 3.68 (dd, *J* = 9.6, 3.4 Hz, 1H, H-3), 3.42 (q, *J* = 1.7 Hz, 1H, H-5), 2.79 – 2.71 (m, 2H, Lev), 2.65 (dd, *J* = 7.1, 5.6 Hz, 2H, Lev), 2.18 (s, 3H, Lev); ^{13}C NMR (101 MHz, $CDCl_3$) δ 206.59 (Lev-CO), 171.25 (Lev-CO), 137.69 (Ar), 135.60 (Ar), 133.55 (Ar), 133.20 (Ar), 133.11 (Ar), 131.75 (Ar), 129.16 (Ar), 128.83 (Ar), 128.31 (Ar), 128.22 (Ar), 128.01 (Ar), 127.95 (Ar), 127.83 (Ar), 126.71 (Ar), 126.58 (Ar), 126.31 (Ar), 126.13 (Ar), 125.86 (Ar), 101.33 (benzylidene-CH), 85.40 (C-1), 78.47 (C-3), 73.38 (C-4), 71.60 (CH_2Ph), 70.04 (C-5), 69.30 (C-6), 68.69 (C-2), 38.00 (Lev-CH_2), 30.03 (Lev-CH_3), 28.22 (Lev-CH_2); $[\alpha]_D^{20}$ = -2.90° (c = 1.0, $CHCl_3$); IR νmax (film) 2983, 1747, 1719, 1366, 1158, 1102, 817, 749, 698 cm^{-1}; HRMS (ESI) calculated for $C_{35}H_{34}O_7S$ $[M+Na]^+$: 621.1917, found: 621.1912.

_N_-(Benzyl)benzyloxycarbonyl-5-amino-pentanyl 4,6-_O_-benzylidene-2-_O_-levulinoyl-3-_O_-(2-naphthylmethyl)-_β_-D-galactopyranoside (7)

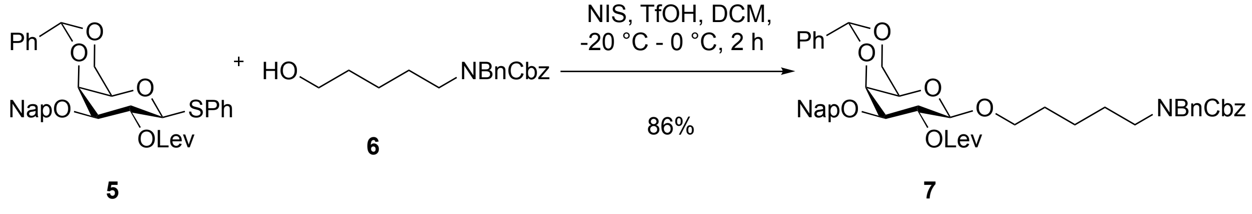

Thioglycoside **5** (1.2 g, 2.0 mmol) and aminopropyl linker **6** (0.98 g, 3.0 mmol) were coevaporated with toluene for three times and dried under high vacuum for 2 h. The mixture was dissolved in anhydrous DCM (25 mL), 4 Å molecular sieves were added and the reaction mixture was stirred

for 30 minutes at room temperature. The reaction was then cooled to 0 °C, NIS (0.68 g, 3.0 mmol) was added along with TfOH (35.4 µL, 0.4 mmol). The reaction mixture was stirred for 2 h. After TLC showed full conversion of starting material, the reaction was quenched with triethylamine (0.1 mL), diluted with DCM (25 mL) and washed with sat. $Na_2S_2O_3$ and brine. The combined organic layer was dried over Na_2SO_4, filtered and concentrated under reduced pressure. The crude product was purified by flash silica column chromatography (hexane/ethyl acetate = 1/1) to give **7** as a colorless syrup (1.41 g, 1.72 mmol, 86%).

^{1}H NMR (400 MHz, $CDCl_3$) δ 7.90 – 7.77 (m, 4H, Ar), 7.60 – 7.47 (m, 5H, Ar), 7.43 – 7.15 (m, 13H, Ar), 5.50 (s, 1H, benzylidene, CH), 5.40 (dd, *J* = 10.1, 8.0 Hz, 1H, H-2), 5.18 (d, *J* = 13.3 Hz, 2H, CH_2Ph), 4.92 – 4.79 (m, 2H, Nap-CH_2), 4.56 – 4.45 (m, 2H, CH_2Ph), 4.37 (t, *J* = 8.1 Hz, 1H, H-1), 4.30 (d, *J* = 11.0 Hz, 1H, H-6), 4.18 (d, *J* = 3.7 Hz, 1H, H-4), 4.02 (dd, *J* = 12.4, 1.9 Hz, 1H, H-6), 3.84 (d, *J* = 6.8 Hz, 1H, linker-CH_2), 3.64 (dd, *J* = 10.0, 3.5 Hz, 1H. H-3), 3.49 – 3.35 (m, 1H, linker-CH_2), 3.36 – 3.11 (m, 3H, H-5, linker-CH_2), 2.81 – 2.45 (m, 4H, Lev), 2.13 (s, 3H, Lev), 1.61 – 1.44 (m, 4H, linker-CH_2), 1.39 – 1.16 (m, 2H, linker-CH_2); ^{13}C NMR (101 MHz, $CDCl_3$) δ 206.62 (Lev-CO, rotamers), 206.55 (Lev-CO, rotamers), 192.59, 171.54 (Lev-CO), 156.82 (Cbz-CO, rotamers), 156.26 (Cbz-CO, rotamers), 138.04 (Ar), 137.70 (Ar), 135.72 (Ar), 134.62 (Ar), 133.23 (Ar), 133.14 (Ar), 129.88 (Ar), 129.12 (Ar), 129.08 (Ar), 128.62 (Ar), 128.60 (Ar), 128.53 (Ar), 128.32 (Ar), 128.24 (Ar), 128.20 (Ar), 128.00 (Ar), 127.95 (Ar), 127.90 (Ar), 127.86 (Ar), 127.30 (Ar), 126.59 (Ar), 126.33 (Ar), 126.12 (Ar), 125.91 (Ar), 101.29 (benzylidene-CH), 101.04 (C-1), 77.36 (C-3), 73.40 (C-4), 71.52 (CH_2Ph), 70.54 (C-2), 69.20 (C-6), 69.03 (linker, rotamers), 68.88 (linker, rotamers), 67.20 (CH_2Ph), 66.61 (C-5), 50.51 (CH_2Ph, rotamers), 50.19 (CH_2Ph, rotamers), 47.17 (linker), 46.23 (linker), 37.96 (Lev-CH_2), 30.00 (Lev-CH_3), 29.23 (linker), 28.09 (Lev-CH_2), 23.22 (linker); $[\alpha]_D^{20}$ = 15.01° (c = 1.0, $CHCl_3$); IR νmax (film) 2931.71, 1745.44, 1698.40, 1497.84, 1455.00, 1421.76, 1366.60, 1151.55, 1106.78, 1082.99, 1059.74, 1027.85, 1002.63, 860.22, 822.03, 735.34, 699.31 cm^{-1}; HRMS (ESI) calculated for $C_{49}H_{53}NO_{10}$ $[M+Na]^+$: 838.3562, found: 838.3542.

***N*-(Benzyl)benzyloxycarbonyl-5-amino-pentanyl 4,6-*O*-benzylidene-2-*O*-levulinoyl-*β*-D-galactopyranoside (1)**

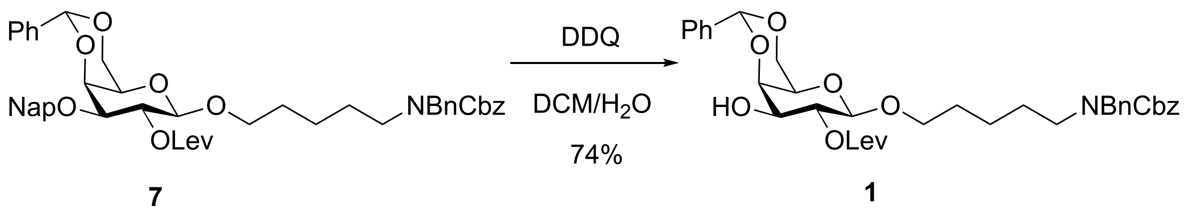

To a solution of **7** (1.2 g, 1.47 mmol) in DCM/H_2O (20 mL/1 mL), DDQ (426 mg, 2.21 mmol) was added and the reaction mixture was stirred for 5 hours under nitrogen atmosphere. The reaction mixture was then diluted with DCM (20 mL), washed with sat. $Na_2S_2O_3$ solution and brine. The organic layer was dried over Na_2SO_4, filtered and concentrated under reduced pressure. The crude product was purified by flash silica column chromatography (hexane/ethyl acetate = 1/2) to give **1** as a colorless syrup (0.74 g, 1.09 mmol, 74%).

^{1}H NMR (400 MHz, $CDCl_3$) δ 7.52 – 7.46 (m, 2H, Ar), 7.41 – 7.13 (m, 13H, Ar), 5.55 (s, 1H, benzylidene, CH), 5.17 (d, *J* = 15.8 Hz, 2H, CH_2Ph), 5.09 (dd, *J* = 10.0, 8.0 Hz, 1H, H-2), 4.49 (d, *J* = 7.3 Hz, 2H, CH_2Ph), 4.40 (t, *J* = 9.0 Hz, 1H, H-1), 4.33 (d, *J* = 12.2 Hz, 1H, H-6), 4.21 (dd, *J* = 3.9, 1.2 Hz, 1H, H-4), 4.15 – 4.04 (m, 1H, H-6), 3.85 (h, *J* = 5.9 Hz, 1H, linker-CH_2), 3.74 (dd, *J* = 10.0, 4.0 Hz, 1H, H-3), 3.52 – 3.36 (m, 2H, H-5, linker-CH_2), 3.32 – 3.14 (m, 2H, linker-CH_2), 2.72 (t, *J* = 7.2 Hz, 2H, Lev), 2.65 – 2.56 (m, 2H, Lev), 2.14 (s, 3H, Lev), 1.65 – 1.45 (m, 4H, linker-CH_2), 1.35 – 1.28 (m, 2H, linker-CH_2); ^{13}C NMR (101 MHz, $CDCl_3$) δ 207.67 (Lev-CO, rotamers), 206.75 (Lev-CO, rotamers), 173.17 (Lev-CO, rotamers), 172.52 (Lev-CO, rotamers), 156.88 (Cbz-CO, rotamers), 156.32 (Cbz-CO, rotamers), 137.97 (Ar), 137.43 (Ar), 134.63 (Ar), 129.90 (Ar), 129.41 (Ar), 129.14 (Ar), 128.68 (Ar), 128.66 (Ar), 128.55 (Ar), 128.38 (Ar), 128.03 (Ar), 127.93 (Ar), 127.78 (Ar), 127.32 (Ar), 126.57 (Ar), 101.61 (benzylidene-CH), 100.71 (C-1), 75.66 (C-4), 72.59 (C-2), 71.76 (C-3), 69.38 (linker), 69.13 (C-6), 67.32 (CH_2Ph), 66.62 (C-5), 50.55 (CH_2Ph, rotamers), 50.27 (CH_2Ph, rotamers), 47.19 (linker), 38.44 (Lev-CH_2, rotamers), 38.16 (Lev-CH_2, rotamers), 30.00 (Lev-CH_3), 29.26 (linker), 28.28 (Lev-CH_2), 28.17 (linker), 23.23 (linker); $[\alpha]_D^{20}$ = -2.82° (c = 1.0, $CHCl_3$); IR νmax (film) 3430.39, 2928.87, 1699.86, 1423.72, 1366.78, 1161.37, 1074.87, 699.96 cm^{-1}; HRMS (ESI) calculated for $C_{38}H_{45}NO_{10}$ $[M+Na]^+$: 698.2936, found: 698.2933.

***p*-Tolyl 2-*O*-(2-naphthylmethyl)-4-*O*-benzyl-1-thio-*α*-L-rhamnopyranoside (9)**

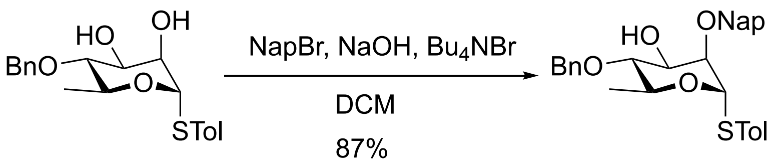

8 9

A 100 mL flask was loaded with compound **8**[3,4] (3 g, 8.32 mmol), tetrabutyl ammonium bromide (5.4 g, 16.75 mmol) and 2-(bromomethyl)naphthalene (4.05 g, 18.32 mmol). DCM (60 mL) and 10% aqueous NaOH (9.6 mL) were added. The reaction mixture was stirred overnight and then diluted with DCM (50 mL). After the mixture was washed with 1 M HCl (50 mL) and brine, the organic layer was dried over Na_2SO_4, filtered and concentrated under reduced pressure. The crude product was purified by flash silica column chromatography (hexane/ethyl acetate = 5/1) to give **9** as a clear syrup (3.62 g, 7.24 mmol, 87%).

^{1}H NMR (400 MHz, $CDCl_3$) δ 7.93 – 7.75 (m, 4H, Ar), 7.57 – 7.47 (m, 3H, Ar), 7.44 – 7.26 (m, 7H, Ar), 7.11 – 7.05 (m, 2H, Ar), 5.51 (d, *J* = 1.7 Hz, 1H, H-1), 4.93 (dd, *J* = 13.9, 11.4 Hz, 2H, CH_2Ph), 4.72 (dd, *J* = 11.4, 8.5 Hz, 2H, CH_2Ph), 4.21 (dq, *J* = 9.4, 6.2 Hz, 1H, H-5), 4.08 (dd, *J* = 3.7, 1.5 Hz, 1H, H-2), 4.01 (dd, *J* = 9.2, 3.8 Hz, 1H, H-3), 3.45 (t, *J* = 9.3 Hz, 1H, H-4), 2.35 (s, 3H, CH_3), 1.39 (d, *J* = 6.2 Hz, 3H, CH_3); ^{13}C NMR (101 MHz, $CDCl_3$) δ 138.52 (Ar), 137.82 (Ar), 134.83 (Ar), 133.30 (Ar), 133.26 (Ar), 132.31 (Ar), 130.54 (Ar), 129.96 (Ar), 128.69 (Ar), 128.59 (Ar), 128.11 (Ar), 128.09 (Ar), 127.92 (Ar), 127.88 (Ar), 127.29 (Ar), 126.46 (Ar), 126.34 (Ar), 126.03 (Ar), 85.61 (C-1), 82.58 (C-4), 80.02 (C-2), 75.32 (CH_2Ph), 72.71 (CH_2Ph), 72.17 (C-3), 68.66 (C-5), 21.27 (CH_3), 18.06 (CH_3); $[\alpha]_D^{20}$ = -79.38° (c = 1.0, $CHCl_3$); IR νmax (film) 3550.65, 2920.67, 1603.25, 1493.58, 1454.84, 1399.18, 1358.37, 1272.52, 1210.82, 1087.67, 1029.57, 893.50, 855.86, 811.60, 787.96, 749.19, 699.23, 667.90 cm^{-1}; HRMS (ESI) calculated for $C_{31}H_{32}O_4S$ $[M+Na]^+$: 523.1913, found:523.1967.

***p*-Tolyl 2-*O*-(2-naphthylmethyl)-3,4-di-*O*-benzyl-1-thio-*α*-L-rhamnopyranoside (4)**

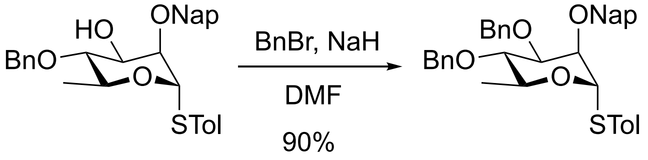

9 4

To a stirred solution of **9** (3.5 g, 7.0 mmol) in anhydrous DMF (23 mL) at 0 °C, NaH (0.559 g of 60% dispersion) was added, the reaction mixture was stirred at 0 °C for 10 minutes. Benzyl bromide (1.66 mL, 14.0 mmol) was then added, the reaction mixture was allowed to warm to room temperature and was stirred for 2 h. After TLC after showed full conversion, the reaction mixture was quenched with sat. $NaHCO_3$ (50 mL), diluted with water (100 mL) and extracted with DCM (50 mL). The organic solution was then washed with brine and dried over Na_2SO_4, filtered and concentrated under reduced pressure. The crude product was purified by flash silica column chromatography (hexane/ethyl acetate = 4/1) to give **4** as a colorless syrup (3.77 g, 6.30 mmol, 90%).

^{1}H NMR (400 MHz, $CDCl_3$) δ 7.90 – 7.74 (m, 4H, Ar), 7.57 – 7.47 (m, 3H, Ar), 7.42 – 7.31 (m, 10H, Ar), 7.27 – 7.20 (m, 2H, Ar), 7.10 – 7.02 (m, 2H, Ar), 5.46 (d, *J* = 1.7 Hz, 1H, H-1), 5.02 (d, *J* = 10.8 Hz, 1H, CH_2Ph), 4.94 – 4.80 (m, 2H, CH_2Ph), 4.74 – 4.58 (m, 3H, CH_2Ph), 4.25 – 4.13 (m, 1H, H-5), 4.05 (dd, *J* = 3.1, 1.7 Hz, 1H, H-2), 3.89 (dd, *J* = 9.3, 3.1 Hz, 1H, H-3), 3.75 (t, *J* = 9.4 Hz, 1H, H-4), 2.34 (s, 3H, CH_3), 1.40 (d, *J* = 6.2 Hz, 3H, CH_3); ^{13}C NMR (101 MHz, $CDCl_3$) δ 138.63 (Ar), 138.36 (Ar), 137.64 (Ar), 135.44 (Ar), 133.29 (Ar), 133.17 (Ar), 132.09 (Ar), 130.83 (Ar), 129.92 (Ar), 129.90 (Ar), 128.55 (Ar), 128.53 (Ar), 128.38 (Ar), 128.17 (Ar), 128.06 (Ar), 127.93 (Ar), 127.83 (Ar), 127.80 (Ar), 127.04 (Ar), 126.24 (Ar), 126.21 (Ar), 126.09 (Ar), 86.37 (C-1), 80.68 (C-4), 80.08 (C-3), 76.43 (C-2), 75.64 (CH_2Ph), 72.32 (CH_2Ph), 72.30 (CH_2Ph), 69.46 (C-5), 21.25 (CH_3), 18.06 (CH_3); $[\alpha]_D^{20}$ = -41.26° (c = 1.0, $CHCl_3$); IR νmax (film) 2871.01, 1493.78, 1454.45, 1366.81, 1088.37, 1029.16, 907.53, 812.07, 737.24, 698.62, 670.35 cm^{-1}; HRMS (ESI) calculated for $C_{38}H_{38}O_4S$ $[M+Na]^+$: 613.2383, found: 613.2381.

***p*-Tolyl 3,4-di-*O*-benzyl-1-thio-*α*-L-rhamnopyranoside (10)**

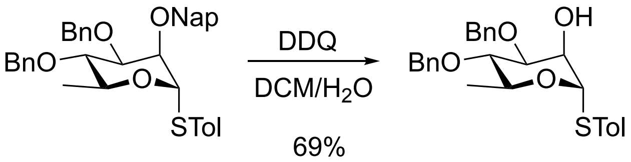

4 **10**

To a solution of **4** (3.2 g, 5.42 mmol) in DCM/H_2O (56 mL/2.8 mL), DDQ (1.84 g, 8.13 mmol) was added and the reaction mixture was stirred for 5 hours under nitrogen atmosphere. The reaction mixture was then diluted with DCM (50 mL), washed with sat. $Na_2S_2O_3$ solution and brine. The organic layer was dried over Na_2SO_4, filtered and concentrated under reduced pressure.

The crude product was purified by flash silica column chromatography (hexane/ethyl acetate = 3/1) to give **10** as a colorless syrup (1.75 g, 3.74 mmol, 69%).

^{1}H NMR (400 MHz, $CDCl_3$) δ 7.44 – 7.31 (m, 12H, Ar), 7.16 – 7.10 (m, 2H, Ar), 5.48 (d, *J* = 1.7 Hz, 1H, H-1), 4.93 (d, *J* = 10.9 Hz, 1H, CH_2Ph), 4.75 (s, 2H, CH_2Ph), 4.68 (d, *J* = 11.0 Hz, 1H, CH_2Ph), 4.28 – 4.19 (m, 2H, H-2, H-4), 3.89 (dd, *J* = 9.0, 3.3 Hz, 1H, H-5), 3.55 (t, *J* = 9.3 Hz, 1H, H-3), 2.35 (s, 3H, CH_3), 1.34 (d, *J* = 6.2 Hz, 3H, CH_3); ^{13}C NMR (101 MHz, $CDCl_3$) δ 138.37 (Ar), 137.78 (Ar), 137.73 (Ar), 132.19 (Ar), 130.31 (Ar), 129.97 (Ar), 128.77 (Ar), 128.62 (Ar), 128.58 (Ar), 128.25 (Ar), 128.14 (Ar), 128.13 (Ar), 127.95 (Ar), 87.43 (C-1), 80.26 (C-3), 80.19 (C-5), 75.61 (CH_2Ph), 72.30 (CH_2Ph), 70.16 (C-2), 68.80 (C-4), 21.26 (CH_3), 17.91 (CH_3); $[\alpha]_D^{20}$ = -199.90° (c = 1.0, $CHCl_3$); IR νmax (film) 3432.76, 2875.96, 1494.20, 1454.95, 1367.44, 1276.46, 1261.58, 1209.82, 1100.79, 1087.51, 980.22, 910.11, 845.39, 809.92, 787.64, 750.39, 698.27 cm^{-1}; HRMS (ESI) calculated for $C_{27}H_{30}O_4S$ $[M+Na]^+$: 473.1757, found:473.1819.

***p*-Tolyl 2-*O*-fluorenylmethyloxycarbonyl-3,4-di-*O*-benzyl-1-thio-*α*-L-rhamnopyranoside (2)**

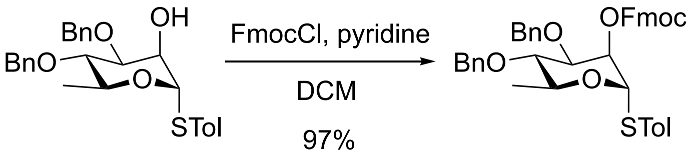

10 **2**

To a stirred solution of **10** (1.7 g, 3.77 mmol) in anhydrous DCM (45 mL), FmocCl (2.0 g, 7.54 mmol) and pyridine (0.91 mL, 11.31 mmol) were added. The reaction mixture was stirred overnight. After TLC showed full conversion, the reaction mixture was diluted with DCM (45 mL), washed with 1M HCl (60 mL), sat. $NaHCO_3$ and brine. The organic solution was dried over Na_2SO_4, filtered and concentrated under reduced pressure. The crude product was purified by flash silica column chromatography (hexane/ethyl acetate = 4/1) to give **2** as a colorless syrup (2.53 g, 3.66 mmol, 97%).

^{1}H NMR (400 MHz, $CDCl_3$) δ 7.84 – 7.77 (m, 2H, Ar), 7.70 – 7.61 (m, 2H, Ar), 7.49 – 7.22 (m, 16H, Ar), 7.18 – 7.12 (m, 2H, Ar), 5.51 – 5.46 (m, 2H, H-1, H-2), 5.01 (d, *J* = 10.9 Hz, 1H, CH_2Ph), 4.80 (d, *J* = 11.4 Hz, 1H, CH_2Ph), 4.68 (dd, *J* = 25.8, 11.1 Hz, 2H, CH_2Ph), 4.50 – 4.43 (m, 1H, Fmoc-CH_2), 4.38 – 4.23 (m, 3H, Fmoc-CH_2, H-5), 3.98 (dd, *J* = 9.3, 2.8 Hz, 1H, H-3), 3.65 (t, *J* =

9.4 Hz, 1H, H-4), 2.36 (s, 3H), 1.41 (d, J = 6.2 Hz, 3H); ^{13}C NMR (101 MHz, $CDCl_3$) δ 154.84 (Fmoc-CO), 143.59 (Ar), 143.47 (Ar), 143.32 (Ar), 141.45 (Ar), 141.41 (Ar), 141.34 (Ar), 138.43 (Ar), 138.20 (Ar), 137.82 (Ar), 132.59 (Ar), 130.05 (Ar), 129.96 (Ar), 128.57 (Ar), 128.50 (Ar), 128.20 (Ar), 128.11 (Ar), 128.09 (Ar), 128.06 (Ar), 128.03 (Ar), 127.98 (Ar), 127.94 (Ar), 127.91 (Ar), 127.33 (Ar), 127.30 (Ar), 125.51 (Ar), 125.34 (Ar), 125.31 (Ar), 120.24 (Ar), 120.18 (Ar), 120.14 (Ar), 86.31 (C-1), 80.17 (C-4), 78.41 (C-3), 75.78 (CH_2Ph), 74.71 (C-2), 72.01 (CH_2Ph), 70.39 (Fmoc-CH_2), 69.24 (C-5), 46.70 (Fmoc-CH), 21.29 (CH_3), 17.92 (CH_3); $[\alpha]_D^{20}$ = -51.86° (c = 1.0, $CHCl_3$); IR νmax (film) 2876.88, 1747.16, 1451.97, 1385.14, 1257.34, 1105.72, 781.13, 759.83, 737.24, 700.26 cm^{-1}; HRMS (ESI) calculated for $C_{42}H_{40}O_6S$ $[M+K]^+$: 711.2177, found:711.2197.

Phenyl 4,6-*O*-di-tertbutylsilyldene-1-thio-*β*-D-galactopyranoside (12)

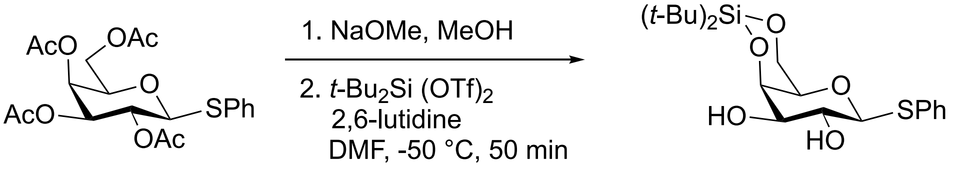

11 **12**

To a solution of phenyl 2,3,4,6-tetra-*O*-acetyl-1-thio-*β*-D-galactopyranoside[5] (2.0 g, 4.54 mmol) in MeOH/DCM (12 mL/ 12 mL), sodium methoxide (5 M solution in methanol) was added until pH of 11 was reached. The reaction mixture was stirred at room temperature for 2 h. The solution was neutralized with amberlite, filtered and concentrated to give a white solid after drying on high vacuum. The intermediate was dissolved in anhydrous DMF (33 mL) and cooled to –50 °C, 2,6-lutidine (1.6 mL, 13.62 mmol) and di-*tert* -butylsilyl bis-trifluormethylsulfonate (1.47 mL, 1.2 equiv) were added. After 50 minutes of stirring at –50 °C, the reaction mixture was diluted with brine (350 mL) and extracted with DCM. Combined organic layer was washed with brine, dried over Na_2SO_4, filtered and concentrated under reduced pressure. The crude product was purified by flash silica column chromatography (hexane/ethyl acetate = 3/2) to give **12** as a colorless syrup (1.64 g, 4.0 mmol, 88%).

^{1}H NMR (400 MHz, $CDCl_3$) δ 7.58 – 7.53 (m, 2H, Ar), 7.34 – 7.27 (m, 3H, Ar), 4.55 (d, J = 9.8 Hz, 1H, H-1), 4.44 (dd, J = 3.5, 1.2 Hz, 1H, H-2), 4.26 (dd, J = 2.1, 1.2 Hz, 2H, H-6), 3.74 (dd, J

= 9.8, 8.8 Hz, 1H, H-3), 3.54 (dd, *J* = 8.8, 3.5 Hz, 1H, H-4), 3.48 (q, *J* = 2.0 Hz, 1H, H-5), 1.05 (s, 9H, *t*-Bu), 1.03 (s, 9H, *t*-Bu); ^{13}C NMR (101 MHz, $CDCl_3$) δ 133.23 (Ar), 132.67 (Ar), 129.05 (Ar), 127.99 (Ar), 89.11 (C-1), 75.24 (C-4), 75.19 (C-5), 72.61 (C-2), 70.72 (C-3), 67.16 (C-6), 27.64 (*t*-Bu-CH_3), 27.50 (*t*-Bu-CH_3), 23.44 (*t*-Bu-$C(CH_3)_3$), 20.74 (*t*-Bu-$C(CH_3)_3$); $[\alpha]_D^{20}$ = -67.52° (c = 1.0, $CHCl_3$); IR νmax (film) 3407.95, 2934.96, 2860.80, 1585.27, 1475.17, 1389.32, 1364.47, 1164.99, 1098.20, 1066.59, 962.50, 918.13, 885.88, 828.58, 810.99, 780.75, 747.11, 691.80 cm^{-1}; HRMS (ESI) calculated for $C_{20}H_{32}O_5SSi$ $[M+Na]^+$: 435.1632, found: 435.1660.

Phenyl 2,3-di-*O*-benzyl-4,6-*O*-di-tertbutylsilyldene-1-thio-*β*-D-galactopyranoside (3)

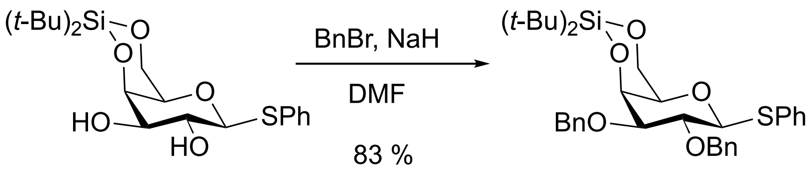

12 **3**

To a stirred solution of **12** (1.6 g, 3.90 mmol) in DMF (15 mL) sodium hydride (374.5 mg, 15.6 mmol) was added at 0 °C. After 10 minutes of stirring at 0 °C, benzyl bromide (1.85 mL, 15.6 mmol) was added dropwise at 0 °C. The reaction was allowed to warm to room temperature and was stirred for 5 h. The reaction mixture was quenched with sat. $NaHCO_3$ (50 mL) and extracted with DCM. The combined organic layer was dried over Na_2SO_4, filtered and concentrated under reduced pressure. The crude product was purified by flash silica column chromatography (hexane/ ethyl acetate = 10/1) to give **3** as a white solid (1.90 g, 3.24 mmol, 83%).

^{1}H NMR (400 MHz, $CDCl_3$) δ 7.57 – 7.50 (m, 2H, Ar), 7.47 – 7.39 (m, 4H, Ar), 7.38 – 7.20 (m, 9H, Ar), 4.91 (s, 2H, CH_2Ph), 4.82 – 4.63 (m, 3H, H-1, CH_2Ph), 4.50 (dd, *J* = 3.1, 0.9 Hz, 1H, H-4), 4.28 – 4.13 (m, 2H, H-6), 3.85 (dd, *J* = 9.8, 9.0 Hz, 1H, H-2), 3.48 (dd, *J* = 9.0, 3.1 Hz, 1H, H-3), 3.29 (t, *J* = 1.5 Hz, 1H, H-5), 1.14 (s, 9H, *t*-Bu), 1.09 (s, 9H, *t*-Bu); ^{13}C NMR (101 MHz, $CDCl_3$) δ 138.46 (Ar), 134.97 (Ar), 132.17 (Ar), 128.88 (Ar), 128.61 (Ar), 128.58 (Ar), 128.46 (Ar), 127.97 (Ar), 127.89 (Ar), 127.40 (Ar), 88.79 (C-1), 82.92 (C-3), 77.33 (C-2), 76.11 (CH_2Ph), 74.84 (C-5), 71.16 (CH_2Ph), 70.09 (C-4), 67.51 (C-6), 27.80 (*t*-Bu-CH_3), 27.77 (*t*-Bu-CH_3), 23.57 (*t*-Bu-$C(CH_3)_3$), 20.86 (*t*-Bu-$C(CH_3)_3$); $[\alpha]_D^{20}$ = 10.70° (c = 1.0, $CHCl_3$); IR νmax (film) 3033.83, 2935.18, 2860.12, 1585.21, 1474.89, 1364.24, 1282.41, 1209.03, 1169.01, 1085.31, 1054.69,

1027.78, 968.17, 920.95, 826.75, 784.18, 745.56, 696.93 cm^{-1}; HRMS (ESI) calculated for $C_{34}H_{44}O_5SSi$ $[M+Na]^+$: 615.2571, found: 615.2596.

***N*-(Benzyl)benzyloxycarbonyl-5-amino-pentanyl 3,4-di-*O*-benzyl-*α*-L-rhamnopyranosyl-(1→3)-4,6-*O*-benzylidene-2-*O*-levulinoyl-*β*-D-galactopyranoside (13)**

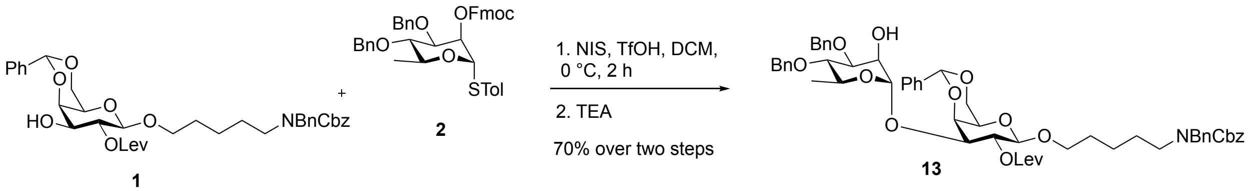

Thioglycoside **1** (427 mg, 0.64 mmol) and compound **2** (330 mg, 0.49 mmol) were coevaporated three times with toluene, the resulting mixture was dried under high vacuum for 2 h. Then, the mixture was dissolved in anhydrous DCM (10 mL) and 4 Å molecular sieves were added. The solution was stirred at room temperature for 30 min and then cooled to 0 °C, NIS (143 mg, 0.64 mmol) and TfOH (8.7 µL, 0.10 mmol) were added. The reaction mixture was stirred at 0 °C for 4 h. After complete consumption of the staring material, triethylamine (0.1 mL) was added and the mixture was stirred for 1 h. The reaction was diluted with DCM (15 mL), filtered and washed with sat. $Na_2S_2O_3$ and brine. The organic layer was dried over Na_2SO_4, filtered and concentrated. The crude product was purified by flash silica column chromatography (hexane/ethyl acetate = 5/1) to give **13** (340 mg, 0.34 mmol, 70% over two steps) as a colorless syrup.

^{1}H NMR (400 MHz, $CDCl_3$) δ 7.47 – 7.40 (m, 2H), 7.33 – 7.05 (m, 23H), 5.44 (s, 1H, benzylidene, CH), 5.23 (dd, *J* = 10.1, 7.9 Hz, 1H), 5.10 (d, *J* = 14.4 Hz, 2H), 4.97 (d, *J* = 1.8 Hz, 1H, Rha-H1), 4.79 (d, *J* = 11.1 Hz, 1H), 4.66 (d, *J* = 11.4 Hz, 1H), 4.52 (dd, *J* = 14.2, 11.2 Hz, 2H), 4.42 (d, *J* = 8.7 Hz, 2H), 4.33 (t, *J* = 8.5 Hz, 1H, Gal-H1), 4.24 (d, *J* = 12.2 Hz, 1H), 4.16 (d, *J* = 3.5 Hz, 1H), 4.10 (dd, *J* = 3.4, 1.7 Hz, 1H), 3.99 (d, *J* = 11.1 Hz, 1H), 3.93 – 3.84 (m, 1H), 3.82 – 3.66 (m, 3H), 3.45 – 3.27 (m, 3H), 3.12 (ddd, *J* = 32.9, 19.7, 12.2 Hz, 3H), 2.88 – 2.70 (m, 1H), 2.63 – 2.40 (m, 2H), 2.38 – 2.24 (m, 1H), 2.06 (s, 3H), 1.54 – 1.36 (m, 4H), 1.27 – 1.15 (m, 5H); ^{13}C NMR (101 MHz, $CDCl_3$) δ 207.46 (Lev-CO, rotamers), 207.33 (Lev-CO, rotamers), 171.42 (Lev-CO), 156.85 (Cbz-CO, rotamers), 156.31 (Cbz-CO, rotamers), 138.73, 138.31, 137.61, 134.62, 129.89, 129.13, 128.96, 128.66, 128.53, 128.50, 128.40, 128.22, 128.19, 128.13, 128.10, 127.92, 127.82, 127.63, 127.41, 127.30, 126.44, 118.72, 102.18 (Rha-C1), 101.21 (benzylidene), 100.94 (Gal-C1), 79.70, 79.64, 79.38, 79.03, 77.93, 75.89, 75.47, 74.94, 71.90, 71.85, 71.73, 70.76, 69.15, 68.35, 68.20,

68.08, 67.32, 67.26, 66.63, 50.52, 50.21, 47.16, 46.20, 37.77, 32.06, 30.06 (Lev-CH_3), 29.83, 29.22, 27.93, 23.24, 22.83, 18.14 (Rha-CH_3); $[\alpha]_D^{20}$ = -9.75° (c = 1.0, $CHCl_3$); IR νmax (film) 3461.28, 2923.40, 1748.36, 1701.78, 1497.78, 1454.84, 1421.58, 1366.46, 1212.40, 1061.98, 736.53, 698.69 cm^{-1}; HRMS (ESI) calculated for $C_{58}H_{67}NO_{14}$ $[M+Na]^+$: 1024.4454, found:1024.9910.

_N_-(Benzyl)benzyloxycarbonyl-5-amino-pentanyl 3,4-di-_O_-benzyl-_α_-L-rhamnopyranosyl-(1→2)-3,4-di-_O_-benzyl-_α_-L-rhamnopyranosyl-(1→3)-4,6-_O_-benzylidene-2-_O_-levulinoyl-_β_-D-galactopyranoside (14)

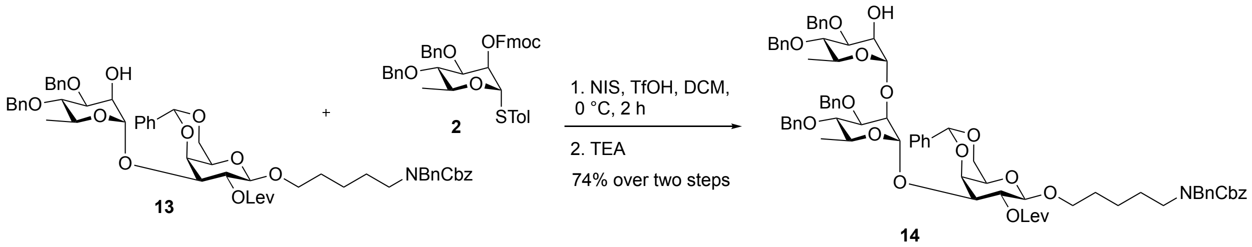

Thioglycoside **2** (344 mg, 0.51 mmol) and compound **13** (395 mg, 0.39 mmol) were coevaporated three times with toluene, the resulting mixture was dried under high vacuum for 2 h. Then, the mixture was dissolved in anhydrous DCM (10 mL) and 4 Å molecular sieves were added. The solution was stirred at room temperature for 30 min and then cooled to 0 °C, NIS (115 mg, 0.51 mmol) and TfOH (7.0 µL, 0.079 mmol) were added. The reaction mixture was stirred at 0 °C for 4 h. After complete consumption of the staring material, triethylamine (0.1 mL) was added and the mixture was stirred for 1 h. The reaction was diluted with DCM (15 mL), filtered and washed with sat. $Na_2S_2O_3$ and brine. The organic layer was dried over Na_2SO_4, filtered and concentrated. The crude product was purified by flash silica column chromatography (toluene/ethyl acetate = 3/1) to give **14** (385 mg, 0.29 mmol, 74% over two steps) as a colorless syrup.

^{1}H NMR (600 MHz, $CDCl_3$) δ 7.56 – 7.50 (m, 2H), 7.46 – 7.17 (m, 35H), 5.53 (s, 1H, benzylidene, CH), 5.30 (td, *J* = 10.1, 4.0 Hz, 1H), 5.25 – 5.11 (m, 3H, Rha-H1), 4.97 (s, 1H, Rha-H1'), 4.92 (dd, *J* = 10.9, 3.8 Hz, 1H), 4.85 (dd, *J* = 11.3, 3.7 Hz, 1H), 4.77 – 4.69 (m, 3H), 4.66 (dd, *J* = 10.9, 3.9 Hz, 1H), 4.61 (dd, *J* = 11.4, 3.8 Hz, 1H), 4.54 (td, *J* = 12.9, 7.0 Hz, 3H), 4.44 (d, *J* = 10.6 Hz, 1H, Gal-H1), 4.36 (d, *J* = 12.5 Hz, 1H), 4.31 – 4.24 (m, 2H), 4.17 (s, 1H), 4.09 (d, *J* = 12.0 Hz, 1H), 3.99 (tq, *J* = 9.7, 6.2 Hz, 1H), 3.93 – 3.80 (m, 4H), 3.73 – 3.64 (m, 1H), 3.54 – 3.41 (m, 3H),

3.38 (t, J = 4.0 Hz, 1H), 3.33 – 3.16 (m, 2H), 2.86 – 2.64 (m, 2H), 2.62 – 2.54 (m, 1H), 2.53 – 2.42 (m, 1H), 2.12 (s, 3H), 1.69 – 1.47 (m, 4H), 1.41 – 1.19 (m, 8H); ^{13}C NMR (151 MHz, $CDCl_3$) δ 206.05 (Lev-CO), 171.44 (Lev-CO), 156.84 (Cbz-CO, rotamers), 156.29 (Cbz-CO, rotamers), 139.02, 138.64, 138.48, 138.08, 137.58, 137.05, 136.90, 128.92, 128.64, 128.55, 128.45, 128.35, 128.29, 128.24, 128.18, 128.09, 128.05, 127.92, 127.64, 127.50, 127.43, 127.33, 126.37, 102.17 (Rha-C1'), 101.35 (Rha-C1), 101.22 (benzylidene), 101.04 (Gal-C1), 80.16, 79.86, 79.67, 78.98, 75.92, 75.57, 74.37, 72.11, 71.89, 70.10, 69.20, 69.06, 68.91, 68.73, 68.38, 67.91, 67.25, 66.59, 50.57, 50.26, 47.21, 46.26, 37.68, 29.98 (Lev-CH_3), 29.24, 27.99, 27.88, 27.54, 23.24, 18.22 (Rha-CH_3), 18.15 (Rha-CH_3); $[\alpha]_D^{20}$ = 7.79° (c = 1.0, $CHCl_3$); IR νmax (film) 2928.56, 1751.39, 1700.60, 1498.01, 1455.05, 1367.48, 1209.75, 1061.05, 821.49, 735.55, 698.28 cm^{-1}; HRMS (ESI) calculated for $C_{78}H_{89}NO_{18}$ $[M+Na]^+$: 1350.5972, found:1350.5967.

_N_-(Benzyl)benzyloxycarbonyl-5-amino-pentanyl 3,4-di-_O_-benzyl-_α_-L-rhamnopyranosyl-(1→2)-3,4-di-_O_-benzyl-_α_-L-rhamnopyranosyl-(1→2)-3,4-di-_O_-benzyl-_α_-L-rhamnopyranosyl-(1→3)-4,6-_O_-benzylidene-2-_O_-levulinoyl-_β_-D-galactopyranoside (15)

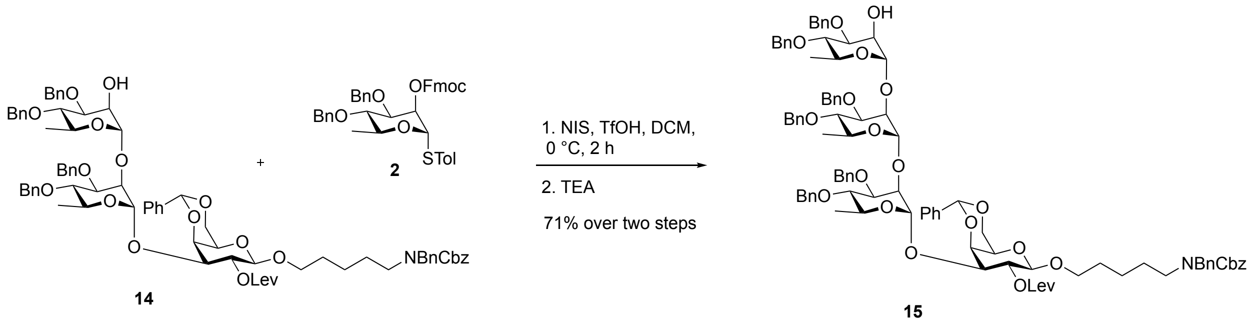

Thioglycoside **2** (253 mg, 0.38 mmol) and compound **14** (385 mg, 0.29 mmol) were coevaporated three times with toluene, the resulting mixture was dried under high vacuum for 2 h. Then, the mixture was dissolved in anhydrous DCM (10 mL) and 4 Å molecular sieves were added. The solution was stirred at room temperature for 30 min and then cooled to 0 °C, NIS (85 mg, 0.38 mmol) and TfOH (5.1 µL, 0.058 mmol) were added. The reaction mixture was stirred at 0 °C for 4 h. After complete consumption of the staring material, triethylamine (0.1 mL) was added and the mixture was stirred for 1 h. The reaction was diluted with DCM (15 mL), filtered and washed with sat. $Na_2S_2O_3$ and brine. The organic layer was dried over Na_2SO_4, filtered and concentrated. The

crude product was purified by flash silica column chromatography (toluene/ethyl acetate = 5/1) to give **15** (340 mg, 0.21 mmol, 71% over two steps) as a colorless syrup.

^{1}H NMR (600 MHz, $CDCl_3$) δ 7.58 – 7.10 (m, 45H), 5.52 (s, 1H, benzylidene, CH), 5.28 (ddd, *J* = 10.5, 7.9, 2.7 Hz, 1H), 5.25 – 5.10 (m, 4H, Rha-H1', Rha-H1''), 4.91 (dd, *J* = 10.8, 2.6 Hz, 3H, , Rha-H1), 4.83 (dd, *J* = 11.3, 2.8 Hz, 1H), 4.77 – 4.61 (m, 7H), 4.54 (ddd, *J* = 19.9, 11.4, 2.7 Hz, 4H), 4.43 (d, *J* = 10.4 Hz, 1H, Gal-H1), 4.35 (d, *J* = 12.4 Hz, 1H), 4.30 – 4.22 (m, 2H), 4.20 – 4.15 (m, 2H), 4.08 (d, *J* = 12.6 Hz, 1H), 4.00 – 3.77 (m, 7H), 3.69 – 3.62 (m, 1H), 3.46 (dtd, *J* = 19.2, 9.4, 2.8 Hz, 4H), 3.37 – 3.18 (m, 3H), 2.70 (q, *J* = 15.5 Hz, 2H), 2.49 (dq, *J* = 22.6, 10.6 Hz, 2H), 2.02 (s, 3H), 1.65 – 1.46 (m, 4H), 1.39 – 1.15 (m, 11H); ^{13}C NMR (151 MHz, $CDCl_3$) δ 205.97 (Lev-CO), 171.38 (Lev-CO), 156.85 (Cbz-CO, rotamers), 156.29 (Cbz-CO, rotamers), 139.03, 138.58, 138.55, 138.51, 138.38, 138.32, 138.14, 137.58, 128.88, 128.65, 128.60, 128.55, 128.53, 128.49, 128.26, 128.22, 128.17, 128.09, 128.06, 128.02, 127.95, 127.93, 127.88, 127.81, 127.67, 127.60, 127.54, 127.42, 127.38, 127.34, 126.34, 102.17 (Rha-C1), 101.18 (benzylidene), 101.05 (Gal-C1), 101.01 (Rha-C1'), 100.51 (Rha-C1''), 80.53, 80.23, 79.76, 79.74, 79.63, 79.43, 78.70, 75.87, 75.58, 75.48, 74.54, 74.34, 72.35, 72.15, 71.71, 70.02, 69.20, 68.74, 68.46, 68.41, 67.96, 67.25, 67.23, 66.58, 50.57, 50.27, 47.23, 46.26, 37.60, 29.93 (Lev-CH_3), 29.25, 27.99, 27.89, 27.54, 23.24, 18.31 (Rha-CH_3), 18.21 (Rha-CH_3), 17.94 (Rha-CH_3); $[\alpha]_D^{20}$ = -4.97° (c = 1.0, $CHCl_3$); IR νmax (film) 2931.24, 1698.66, 1455.13, 1365.13, 1066.06, 735.77, 698.36 cm^{-1}; HRMS (ESI) calculated for $C_{98}H_{111}NO_{22}$ $[M+Na]^+$: 1676.7490, found:1676.7572.

***N*-(Benzyl)benzyloxycarbonyl-5-amino-pentanyl 2,3-di-*O*-benzyl-4,6-*O*-di-tertbutylsilyldene-*α*-D-galactopyranosyl-(1→2)-3,4-di-*O*-benzyl-*α*-L-rhamnopyranosyl-(1→2)-3,4-di-*O*-benzyl-*α*-L-rhamnopyranosyl-(1→2)-3,4-di-*O*-benzyl-*α*-L-rhamnopyranosyl-(1→3)-4,6-*O*-benzylidene-2-*O*-levulinoyl-*β*-D-galactopyranoside (16)**

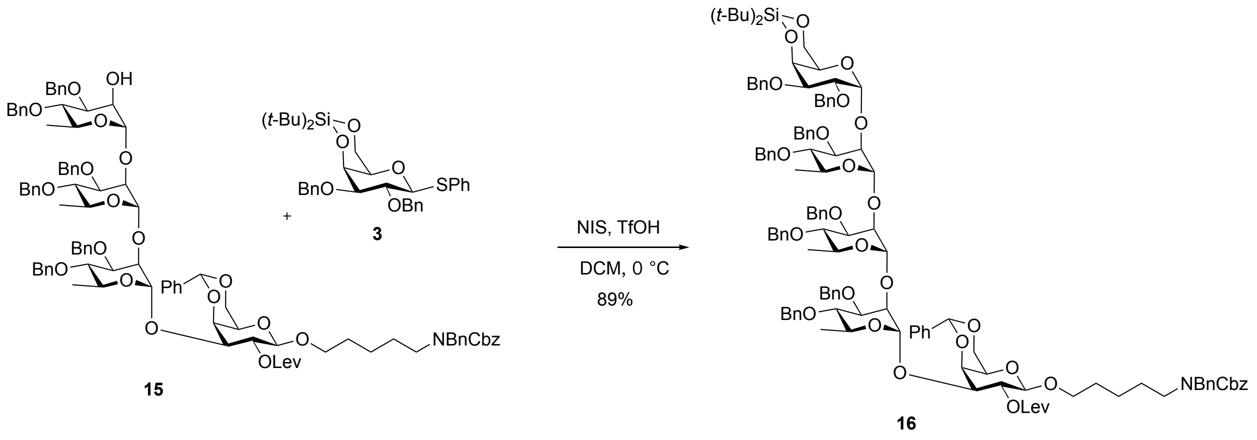

Thioglycoside **3** (149 mg, 0.25 mmol) and compound **15** (320 mg, 0.19 mmol) were coevaporated three times with toluene, the resulting mixture was dried under high vacuum for 2 h. Then, the mixture was dissolved in anhydrous DCM (10 mL) and 4 Å molecular sieves were added. The solution was stirred at room temperature for 30 min and then cooled to 0 °C, NIS (57 mg, 0.25 mmol) and TfOH (3.4 μL, 0.039 mmol) were added. The reaction mixture was stirred at 0 °C for 4 h. After complete consumption of the staring material, triethylamine (0.1 mL) was added. The reaction was diluted with DCM (15 mL), filtered and washed with sat. $Na_2S_2O_3$ and brine. The organic layer was dried over Na_2SO_4, filtered and concentrated. The crude product was purified by flash silica column chromatography (toluene/ethyl acetate = 7/1) to give **16** (366 mg, 0.17, 89%) as a colorless syrup.

^{1}H NMR (600 MHz, $CDCl_3$) δ 8.01 – 7.08 (m, 55H), 5.52 (s, 1H, benzylidene, CH), 5.28 (dd, *J* = 10.1, 8.0 Hz, 1H), 5.23 – 5.14 (m, 3H, Rha-H1''), 5.10 (t, *J* = 1.7 Hz, 1H, Rha-H1'), 4.99 – 4.91 (m, 3H, Gal-H1'), 4.86 (ddd, *J* = 18.8, 9.4, 3.5 Hz, 2H, Rha-H1), 4.78 (dt, *J* = 11.8, 3.6 Hz, 2H), 4.68 (dddd, *J* = 26.5, 21.1, 13.6, 10.5 Hz, 7H), 4.60 – 4.48 (m, 6H), 4.45 – 4.32 (m, 2H, Gal-H1), 4.30 – 4.17 (m, 3H), 4.11 – 4.01 (m, 2H), 4.00 – 3.77 (m, 10H), 3.76 – 3.69 (m, 2H), 3.65 (ddd, *J* = 17.3, 10.2, 3.7 Hz, 1H), 3.57 – 3.35 (m, 5H), 3.33 – 3.18 (m, 2H), 2.79 – 2.56 (m, 2H), 2.51 – 2.39 (m, 2H), 2.00 – 1.92 (m, 3H), 1.66 – 1.48 (m, 4H), 1.37 – 1.20 (m, 11H), 1.08 (s, 9H), 1.05 (s, 9H); ^{13}C NMR (151 MHz, $CDCl_3$) δ 206.23 (Lev-CO), 171.66 (Lev-CO), 156.88 (Cbz-CO), 139.41, 139.00, 138.63, 138.34, 138.07, 134.60, 129.89, 129.14, 128.88, 128.66, 128.60, 128.55, 128.48, 128.37, 128.25, 128.19, 128.12, 127.93, 127.80, 127.59, 127.53, 127.47, 127.16, 126.34, 102.19 (Rha-C1), 101.76 (Rha-C1''), 101.27 (Gal-C1, benzylidene), 98.60 (Rha-C1'), 96.79 (Gal-C1'), 80.70, 80.40, 79.80, 75.88, 75.46, 74.29, 73.74, 72.85, 72.23, 71.81, 71.64, 71.37, 71.01,

70.82, 70.46, 70.02, 69.23, 68.76, 68.46, 67.31, 62.61, 50.60, 50.32, 47.24, 46.28, 37.56, 29.95, 29.26, 27.82 (*t*-Bu-CH_3), 27.58 (*t*-Bu-CH_3), 23.50, 23.25 (*t*-Bu-$C(CH_3)_3$), 20.80 (*t*-Bu-$C(CH_3)_3$) 18.30 (Rha-CH_3, overlapped); $[\alpha]_D^{20}$ = 37.26° (c = 1.0, $CHCl_3$); IR νmax (film) 2934.47, 1749.32, 1700.01, 1497.96, 1455.45, 1364.92, 1209.22, 1069.60, 915.15, 827.19, 736.53, 698.44 cm^{-1}; HRMS (ESI) calculated for $C_{126}H_{149}NO_{27}Si$ $[M+Na]^+$: 2158.9979, found: 2158.9975.

N-(Benzyl)benzyloxycarbonyl-5-amino-pentanyl 2,3-di-*O*-benzyl-*α*-D-galactopyranosyl-(1→2)-3,4-di-*O*-benzyl-*α*-L-rhamnopyranosyl-(1→2)-3,4-di-*O*-benzyl-*α*-L-rhamnopyranosyl-(1→2)-3,4-di-*O*-benzyl-*α*-L-rhamnopyranosyl-(1→3)-4,6-*O*-benzylidene-2-*O*-levulinoyl-*β*-D-galactopyranoside (17)

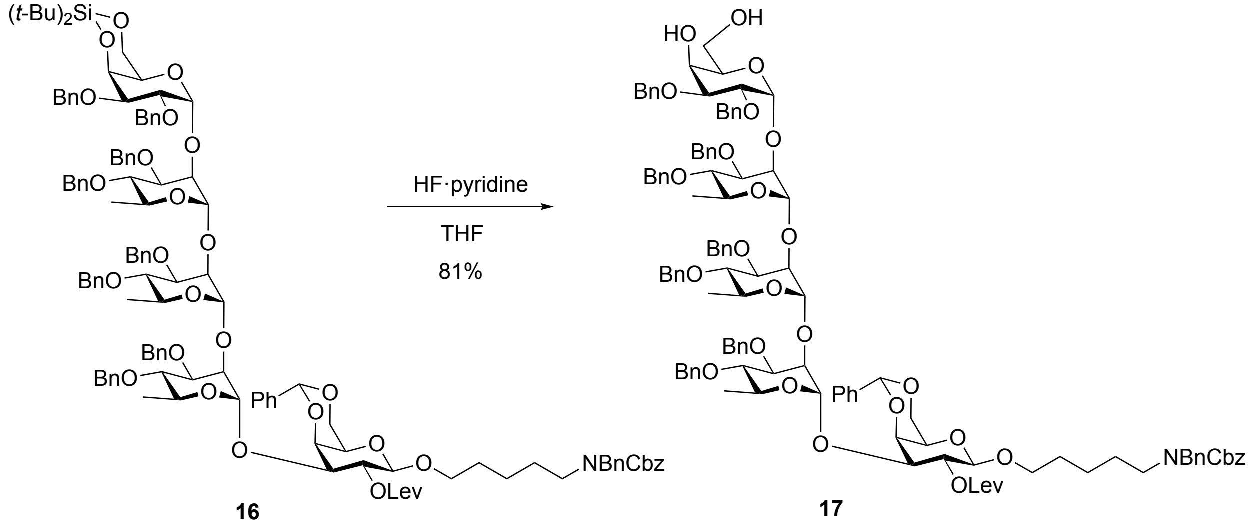

To a solution of **16** (366mg, 0.17 mmol) in THF (6 mL) was added HF·pyridine (70% HF, 44 μL, 1.71 mmol). After TLC analysis indicated complete conversion of the starting material, the reaction was quenched with triethylamine. The mixture was concentrated, the resulting residue was dissolved in ethyl acetate and subsequently washed with saturated aqueous $NaHCO_3$ and brine. The aqueous layers were then extracted with ethyl acetate, the combined organic layer was dried over Na_2SO_4, filtered and concentrated under reduced pressure. The crude product was purified by flash silica column chromatography (toluene/ethyl acetate = 3/1) afforded **17** (278 mg, 0.14 mmol, 81%) as a colorless syrup.

^1H NMR (600 MHz, $CDCl_3$) δ 7.55 – 7.12 (m, 55H), 5.52 (s, 1H, benzylidene, CH), 5.28 (dd, *J* = 10.2, 8.0 Hz, 1H), 5.24 – 5.13 (m, 4H, Rha-H1', Rha-H1''), 5.00 – 4.89 (m, 4H, Rha-H1, Gal-H1'), 4.84 (d, *J* = 11.2 Hz, 1H), 4.80 – 4.64 (m, 7H), 4.63 – 4.48 (m, 7H), 4.47 – 4.39 (m, 2H, Gal-H1), 4.35 (d, *J* = 12.3 Hz, 1H), 4.24 (dt, *J* = 23.1, 4.5 Hz, 4H), 4.12 – 4.01 (m, 3H), 4.00 – 3.72 (m, 9H), 3.69 – 3.62 (m, 2H), 3.62 – 3.53 (m, 2H), 3.47 (dd, *J* = 18.6, 9.2 Hz, 3H), 3.37 (t, *J* = 9.5 Hz, 1H), 3.25 (dt, *J* = 40.9, 6.9 Hz, 2H), 2.77 – 2.63 (m, 2H), 2.47 (q, *J* = 8.6 Hz, 2H), 1.99 (s, 3H), 1.65 – 1.49 (m, 4H), 1.38 – 1.19 (m, 11H); ^{13}C NMR (151 MHz, $CDCl_3$) δ 206.03 (Lev-CO), 171.41 (Lev-CO), 156.86 (Cbz-CO, rotamers), 156.29 (Cbz-CO, rotamers), 139.02, 138.87, 138.71, 138.69, 138.57, 138.53, 138.28, 138.22, 138.10, 137.59, 129.17, 128.88, 128.65, 128.55, 128.48, 128.46, 128.34, 128.27, 128.23, 128.20, 128.13, 128.01, 127.94, 127.72, 127.63, 127.55, 127.49, 127.34, 126.34, 102.17 (Rha-C1), 101.17 (benzylidene), 101.14 (Gal-C1), 101.01 (Rha-C1'), 98.12 (Rha-C1''), 95.42 (Gal-C1'), 80.64, 79.76, 79.66, 78.63, 78.59, 75.87, 75.53, 75.41, 74.29, 73.85, 72.93, 72.59, 72.38, 72.04, 71.90, 71.63, 70.01, 69.20, 69.18, 69.15, 68.78, 68.52, 68.40, 67.25, 66.58, 63.22, 50.58, 50.28, 47.23, 46.27, 37.55, 29.94 (Lev-CH_3), 29.25, 27.88, 23.24, 18.29 (Rha-CH_3), 18.22 (Rha-CH_3), 18.15 (Rha-CH_3); $[\alpha]_D^{20}$ = 40.44° (c = 1.0, $CHCl_3$); IR νmax (film) 2924.74, 1699.12, 1455.25, 1365.24, 1062.21, 738.12, 698.25 cm^{-1}; HRMS (ESI) calculated for $C_{118}H_{133}NO_{27}$ $[M+Na]^+$: 2018.8957, found: 2018.9022.

***N*-(Benzyl)benzyloxycarbonyl-5-amino-pentanyl 2,3-di-*O*-benzyl-6-*O*-fluorenylmethyloxycarbonyl-*α*-D-galactopyranosyl-(1→2)-3,4-di-*O*-benzyl-*α*-L-rhamnopyranosyl-(1→2)-3,4-di-*O*-benzyl-*α*-L-rhamnopyranosyl-(1→2)-3,4-di-*O*-benzyl-*α*-L-rhamnopyranosyl-(1→3)-4,6-*O*-benzylidene-2-*O*-levulinoyl-*β*-D-galactopyranoside (18)**

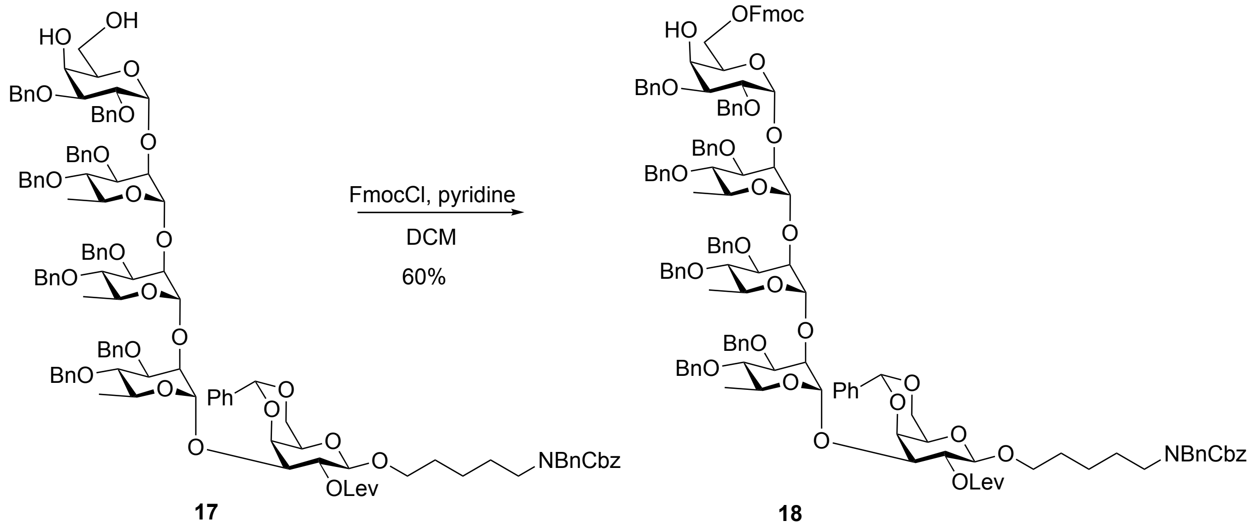

To a stirred solution of diol **17** (100 mg, 0.05 mmol) in anhydrous DCM (1.0 mL) FmocCl (16.8 mg, 0.065 mmol) and pyridine (0.3 mL) was added. The reaction mixture was stirred for 4 h at room temperature under nitrogen atmosphere. The reaction mixture was diluted with DCM (20 mL) and washed with 1 M HCl (20 mL) and brine, the organic layer was dried over Na_2SO_4, filtered and concentrated under reduced pressure. The crude product was purified by flash silica column chromatography (toluene/ethyl acetate = 5/1) afforded **18** (67 mg, 0.03 mmol, 60%) as a colorless syrup.

^{1}H NMR (400 MHz, $CDCl_3$) δ 7.65 (dd, *J* = 7.6, 2.7 Hz, 2H), 7.48 – 7.37 (m, 4H), 7.34 – 7.01 (m, 57H), 5.41 (s, 1H, benzylidene, CH), 5.17 (dd, *J* = 10.1, 7.9 Hz, 1H), 5.13 – 5.02 (m, 4H, Rha-H1', Rha-H1''), 4.87 – 4.68 (m, 6H, Rha-H1, Gal-H1'), 4.68 – 4.59 (m, 4H), 4.58 – 4.39 (m, 8H), 4.38 – 4.27 (m, 3H, Gal-H1), 4.27 – 4.12 (m, 8H), 4.10 (t, *J* = 2.4 Hz, 1H), 4.04 (t, *J* = 7.4 Hz, 1H), 4.00 – 3.93 (m, 2H), 3.86 (ddd, *J* = 19.2, 9.7, 3.3 Hz, 3H), 3.81 – 3.60 (m, 6H), 3.55 (dd, *J* = 10.1, 3.5 Hz, 1H), 3.49 – 3.22 (m, 5H), 3.13 (dt, *J* = 22.9, 7.8 Hz, 2H), 2.65 – 2.49 (m, 2H), 2.42 – 2.26 (m, 2H), 1.87 (s, 3H), 1.55 – 1.38 (m, 4H), 1.25 – 1.09 (m, 11H); ^{13}C NMR (101 MHz, $CDCl_3$) δ 206.06 (Lev-CO), 171.44 (Lev-CO), 156.84 (Cbz-CO, rotamers), 156.30 (Cbz-CO, rotamers), 155.03 (Fmoc-CO), 143.46, 143.36, 141.36, 141.34, 138.99, 138.84, 138.76, 138.69, 138.50, 138.45, 138.41, 138.14, 138.07, 137.55, 137.03, 136.89, 129.90, 129.14, 129.11, 128.89, 128.74, 128.65, 128.56, 128.54, 128.48, 128.44, 128.40, 128.31, 128.27, 128.23, 128.21, 128.17, 128.10, 128.05, 127.99, 127.94, 127.88, 127.79, 127.72, 127.67, 127.64, 127.58, 127.55, 127.47, 127.38, 127.33, 127.27, 127.24, 126.33, 125.32, 125.23, 120.15, 102.17 (Rha-C1), 101.15 (Gal-C1,

benzylidene), 101.00 (Rha-C1'), 98.17 (Rha-C1''), 95.46 (Gal-C1'), 80.70, 80.48, 79.72, 79.67, 78.54, 78.12, 75.86, 75.58, 75.47, 75.41, 74.28, 74.14, 72.92, 72.57, 72.35, 71.87, 71.70, 71.58, 70.00, 69.97, 69.21, 69.03, 68.87, 68.73, 68.44, 68.35, 67.34, 67.31, 67.25, 66.73, 66.56, 50.54, 50.25, 47.21, 46.72, 46.25, 37.51, 29.95 (Lev-CH_3), 29.84, 29.25, 27.99, 27.84, 27.54, 23.23, 18.26 (Rha-CH_3), 18.21 (Rha-CH_3), 18.14 (Rha-CH_3); $[\alpha]_D^{20}$ = 40.15° (c = 1.0, $CHCl_3$); IR νmax (film) 2934.41, 1751.88, 1699.76, 1454.72, 1367.57, 1261.51, 1063.10, 739.72, 698.89 cm^{-1}; HRMS (ESI) calculated for $C_{133}H_{143}NO_{29}$ $[M+Na]^+$: 2240.9638, found: 2240.9717.

***N*-(Benzyl)benzyloxycarbonyl-5-amino-pentanyl 2-*O*-(2-naphthylmethyl)-3,4-di-*O*-benzyl-*α*-L-rhamnopyranosyl-(1→4)-2,3-di-*O*-benzyl-6-*O*-fluorenylmethyloxycarbonyl-*α*-D-galactopyranosyl-(1→2)-3,4-di-*O*-benzyl-*α*-L-rhamnopyranosyl-(1→2)-3,4-di-*O*-benzyl-*α*-L-rhamnopyranosyl-(1→2)-3,4-di-*O*-benzyl-*α*-L-rhamnopyranosyl-(1→3)-4,6-*O*-benzylidene-2-*O*-levulinoyl-*β*-D-galactopyranoside (19)**

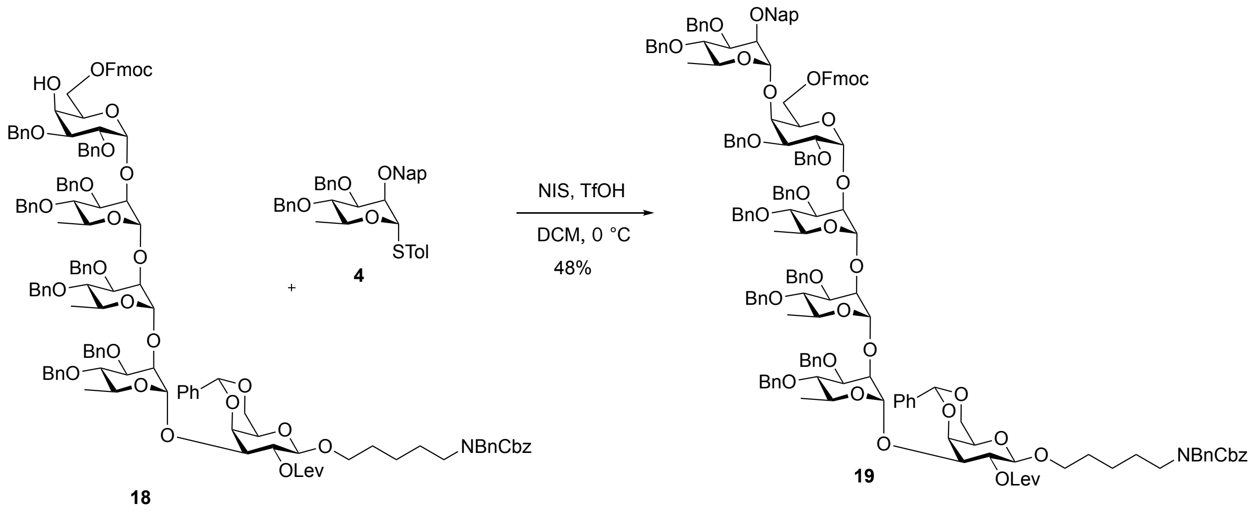

Thioglycoside **4** (30 mg, 0.051 mmol) and compound **18** (60 mg, 0.027 mmol) were coevaporated three times with toluene, the resulting mixture was dried under high vacuum for 2 h. Then, the mixture was dissolved in anhydrous DCM (1 mL) and 4 Å molecular sieves were added. The solution was stirred at room temperature for 30 min and then cooled to 0 °C, NIS (4.1 mg, 0.018 mmol) and TfOH (0.2 µL, 0.0018 mmol) were added. The reaction mixture was stirred at 0 °C for 4 h. After complete consumption of the staring material, the reaction was diluted with DCM (15 mL), filtered and washed with sat. $NaHCO_3$, sat. $Na_2S_2O_3$ and brine. The organic layer was dried over Na_2SO_4, filtered and concentrated. The crude product was purified by flash silica column

chromatography (toluene/ethyl acetate = 8/1) to give **19** (35 mg, 0.013 mmol, 48%) as a colorless syrup.

^{1}H NMR (700 MHz, $CDCl_3$) δ 7.77 – 7.55 (m, 7H), 7.52 (s, 1H), 7.45 – 6.99 (m, 72H), 5.40 (s, 1H, benzylidene, CH), 5.29 (s, 1H, Gal-H1'), 5.16 (t, *J* = 9.1 Hz, 1H), 5.13 – 5.06 (m, 2H), 5.02 (d, *J* = 6.7 Hz, 2H, Rha-H1', Rha-H1''), 4.86 (d, *J* = 11.0 Hz, 1H), 4.80 (dd, *J* = 11.0, 7.0 Hz, 3H), 4.76 – 4.67 (m, 5H, Rha-H1, Rha-H1'''), 4.66 – 4.55 (m, 7H), 4.54 – 4.45 (m, 6H), 4.42 (td, *J* = 14.9, 5.7 Hz, 4H), 4.31 (dt, *J* = 20.1, 9.3 Hz, 2H, Gal-H1), 4.28 – 4.20 (m, 2H), 4.18 – 4.11 (m, 5H), 4.11 – 4.05 (m, 3H), 4.02 (t, *J* = 7.3 Hz, 1H), 3.98 – 3.90 (m, 3H), 3.89 – 3.80 (m, 3H), 3.80 – 3.67 (m, 5H), 3.63 (dd, *J* = 9.5, 6.2 Hz, 1H), 3.60 – 3.51 (m, 3H), 3.46 – 3.40 (m, 1H), 3.39 – 3.29 (m, 3H), 3.25 (q, *J* = 7.8 Hz, 1H), 3.21 – 3.06 (m, 2H), 2.62 – 2.50 (m, 2H), 2.40 – 2.29 (m, 2H), 1.87 (s, 3H), 1.53 – 1.38 (m, 4H), 1.28 – 1.09 (m, 11H); ^{13}C NMR (176 MHz, $CDCl_3$) δ 205.98 (Lev-CO), 171.42 (Lev-CO), 154.85 (Cbz-CO), 143.52, 143.46, 141.45, 141.36, 139.06, 138.86, 138.84, 138.79, 138.76, 138.74, 138.71, 138.54, 138.50, 138.48, 138.39, 138.11, 137.91, 137.62, 136.17, 134.59, 133.34, 133.04, 129.89, 129.73, 129.14, 129.09, 128.89, 128.75, 128.73, 128.71, 128.66, 128.64, 128.61, 128.58, 128.56, 128.54, 128.49, 128.43, 128.42, 128.40, 128.38, 128.35, 128.28, 128.24, 128.22, 128.20, 128.17, 128.15, 128.12, 128.11, 128.08, 128.04, 128.00, 127.97, 127.95, 127.94, 127.89, 127.87, 127.79, 127.77, 127.69, 127.64, 127.60, 127.56, 127.54, 127.50, 127.47, 127.45, 127.43, 127.38, 127.35, 127.31, 127.27, 127.13, 126.51, 126.39, 126.36, 126.15, 126.11, 126.04, 125.90, 125.83, 125.35, 125.31, 120.21, 120.16, 120.12, 103.16 (Rha-C1), 102.19, 101.19 (Rha-C1'), 101.16 (Gal-C1), 101.03 (benzylidene), 99.80 (Rha-C1'''), 98.20 (Rha-C1''), 95.97, 95.34 (Gal-C1'), 92.11, 80.78, 80.48, 80.45, 80.08, 79.83, 79.80, 79.68, 79.62, 79.34, 78.62, 78.10, 76.08, 75.89, 75.67, 75.57, 75.47, 75.38, 74.86, 74.40, 74.30, 73.90, 73.70, 73.24, 72.83, 72.35, 72.19, 72.12, 71.91, 71.84, 71.68, 71.60, 70.85, 70.22, 70.07, 70.04, 69.31, 69.22, 68.75, 68.50, 68.40, 68.14, 67.66, 67.32, 67.27, 66.73, 66.61, 66.31, 50.61, 50.33, 46.91, 46.76, 37.56, 29.93 (Lev-CH_3), 29.28, 27.90, 23.26, 18.26 (Rha-CH_3), 18.23 (Rha-CH_3), 18.16 (Rha-CH_3); $[\alpha]_D^{20}$ = 24.05° (c = 1.0, $CHCl_3$); IR νmax (film) 2930.86, 1751.92, 1454.69, 1261.27, 1075.89, 739.87, 699.49 cm^{-1}; HRMS (ESI) calculated for $C_{164}H_{173}NO_{33}$ $[M+Na]^+$: 2707.1782, found: 2707.1914.

5-Aminopentyl *α*-L-rhamnopyranosyl-(1→4)-*α*-D-galactopyranosyluronate-(1→2)-*α*-L-rhamnopyranosyl-(1→2)-*α*-L-rhamnopyranosyl-(1→2)-*α*-L-rhamnopyranosyl-(1→3)-*β*-D-galactopyranoside (20)

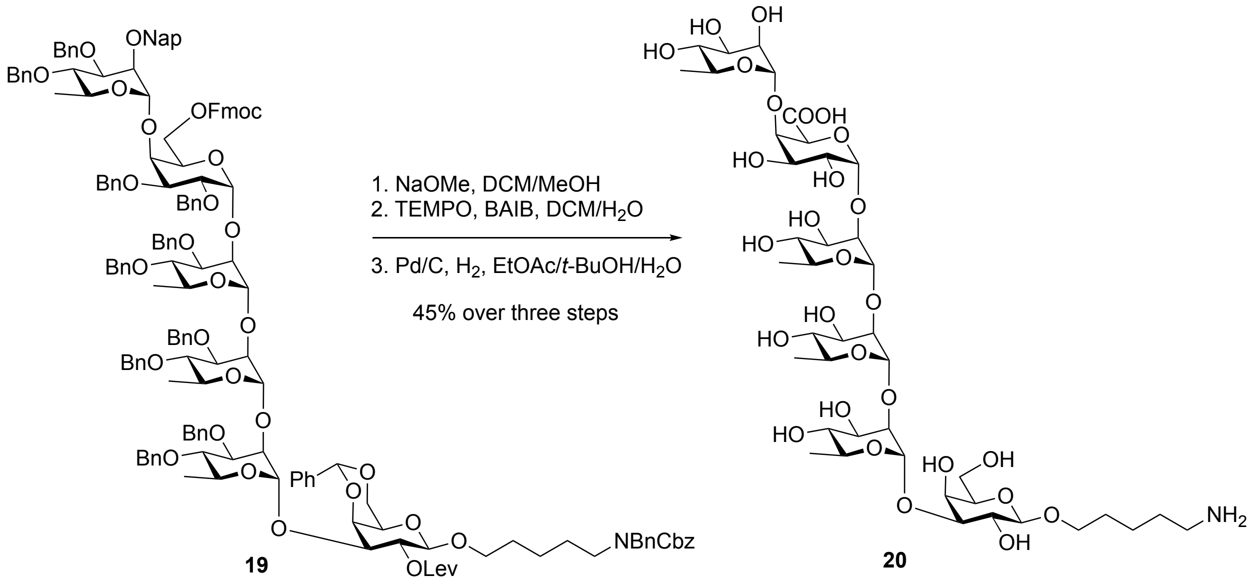

NaOMe was added slowly to a solution of **19** (22 mg, 0.008 mmol) in MeOH/DCM = 1/1 (1 mL) until the solution reached pH 11. The reaction mixture was stirred for 1 h, neutralized with Amberlite, filtered and concentrated under reduced pressure. The residue was dissolved in DCM (1.5 mL) and H_2O (0.5 mL). 2,2,6,6-Tetramethylpiperidine-1-oxyl (6.3 mg, 0.004 mmol) was added followed by $PhI(OAc)_2$ (64 mg, 0.02 mmol) at 0 °C. The reaction mixture was warmed to room temperature, after 4 h, the reaction mixture was quenched by the addition of saturated aqueous $Na_2S_2O_3$, and the organic layer was washed with brine. The separated organic layer was dried over Na_2SO_4 and concentrated under reduced pressure. The crude product was then dissolved in EtOAc/*t*-BuOH/H_2O (2 mL/2 mL/1 mL), palladium on activated charcoal (10% Pd, 30 mg) was added and the reaction mixture was stirred under a hydrogen atmosphere for 8 h. After complete consumption of the starting material, the reaction mixture was filtered and the crude product obtained after solvent removal was purified by reversed phase HPLC using a preparative Hypercarb column (From 0% to 30% MeCN in H_2O in 30 min, flow rate 3 mL/min, t_R = 21.6 min) to afford **20** (3.6 mg, 0.0036 mmol, 45%) as a white solid.

^{1}H NMR (600 MHz, D_2O) δ 5.11 (q, *J* = 2.0 Hz, 1H, Rha-H1’’’), 5.07 – 5.04 (m, 1H, Rha-H1’’), 5.04 – 5.01 (m, 1H, Rha-H1’), 4.97 (d, *J* = 3.0 Hz, 1H, Rha-H1), 4.95 – 4.92 (m, 1H, Gal-H1’),

4.56 – 4.50 (m, 1H), 4.34 – 4.27 (m, 2H, Gal-H1), 4.03 – 3.92 (m, 5H), 3.89 – 3.75 (m, 6H), 3.72 – 3.55 (m, 10H), 3.54 – 3.48 (m, 1H), 3.43 – 3.34 (m, 3H), 3.24 (td, *J* = 9.9, 5.0 Hz, 1H), 2.89 (td, *J* = 7.6, 4.9 Hz, 2H), 1.63 – 1.52 (m, 4H), 1.39 – 1.29 (m, 2H), 1.23 – 1.08 (m, 14H); ^{13}C NMR (151 MHz, D_2O) δ 174.94 (COOH), 102.50 (Gal-C1), 100.76 (Rha-C1', Rha-C1''), 100.50 (Rha-C1'''), 99.70 (Rha-C1), 97.47 (Gal-C1), 79.79, 78.42, 77.94, 76.22, 75.67, 75.05, 72.10, 71.99, 71.65, 71.35, 70.48, 70.31, 69.98, 69.87, 69.24, 68.67, 68.32, 67.82, 60.78 (linker), 39.29 (linker), 28.09 (linker), 26.35 (linker), 22.03 (linker), 16.59 (Rha-CH_3), 16.55 (Rha-CH_3, overlapped), 16.46 (Rha-CH_3); HRMS (ESI) calculated for $C_{41}H_{71}NO_{28}$ [M+H]$^+$: 1026.4235, found: 1026.4307.

5-Aminopentyl *α*-L-rhamnopyranosyl-(1→3)-*β*-D-galactopyranoside (21)

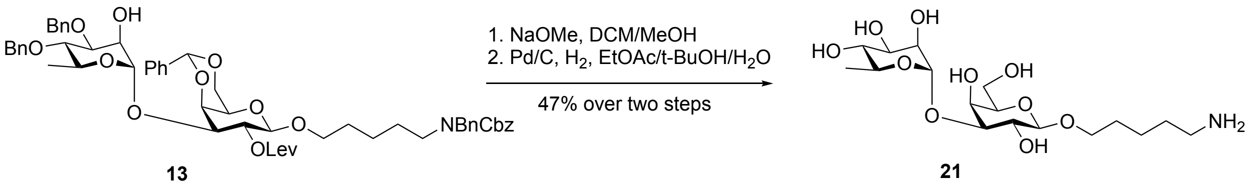

NaOMe was added slowly to a solution of **13** (20 mg, 0.02 mmol) in MeOH/DCM = 1/1 (1 mL) until the solution reached pH 11. The reaction mixture was stirred for 1 h, neutralized with Amberlite, filtered and concentrated under reduced pressure. The residue was dissolved in EtOAc/*t*-BuOH/H_2O (2 mL/2 mL/1 mL), palladium on activated charcoal (10% Pd, 30 mg) was added and the reaction mixture was stirred under a hydrogen atmosphere for 8 h. After complete consumption of the starting material, the reaction mixture was filtered and the crude product obtained after solvent removal was purified by reversed phase HPLC using a preparative Hypercarb column (From 0% to 30% MeCN in H_2O in 30 min, flow rate 3 mL/min, t_R = 16.0 min) to afford **21** (3.9 mg, 0.01 mmol. 47%) as a white solid.

^{1}H NMR (600 MHz, D_2O) δ 5.01 (d, *J* = 1.9 Hz, 1H, Rha-H1), 4.43 (dd, *J* = 7.9, 1.0 Hz, 1H, Gal-H1), 4.05 (dd, *J* = 3.4, 1.8 Hz, 1H), 3.98 (d, *J* = 3.4 Hz, 1H), 3.93 (dt, *J* = 10.0, 6.6 Hz, 1H), 3.85 – 3.76 (m, 2H), 3.76 – 3.65 (m, 5H), 3.59 (dd, *J* = 10.0, 7.9 Hz, 1H), 3.45 (t, *J* = 9.7 Hz, 1H), 2.99 (t, *J* = 7.5 Hz, 2H), 1.73 – 1.62 (m, 4H), 1.49 – 1.41 (m, 2H), 1.26 (d, *J* = 6.3 Hz, 3H); ^{13}C NMR (151 MHz, D_2O) δ 102.43 (Gal-C1), 102.37 (Rha-C1), 80.49, 75.06, 71.88, 70.11, 70.02, 70.00, 69.92, 69.14, 68.36, 60.81 (linker), 39.30 (linker), 28.10 (linker), 26.37 (linker), 22.04 (linker), 16.55 (Rha-CH_3); HRMS (ESI) calculated for $C_{17}H_{33}NO_{10}$ [M+H]$^+$: 412.2177, found: 412.2203.

5-Aminopentyl α-L-rhamnopyranosyl-(1→2)-α-L-rhamnopyranosyl-(1→3)-β-D-galactopyranoside (22)

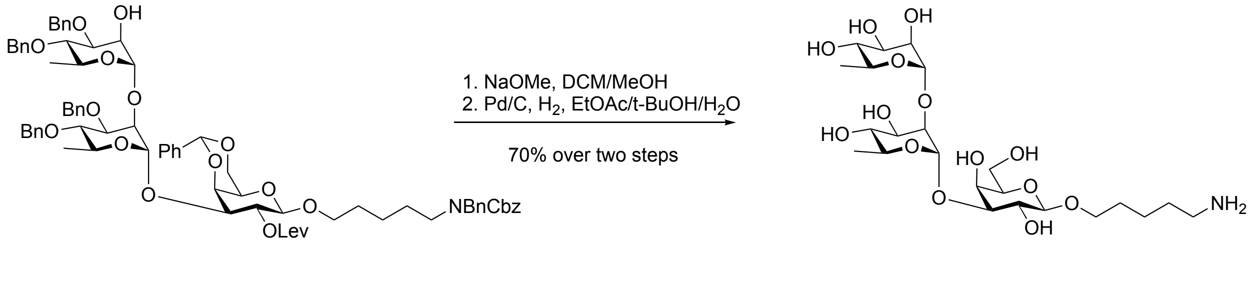

14 **22**

NaOMe was added slowly to a solution of **14** (20 mg, 0.015 mmol) in MeOH/DCM = 1/1 (1 mL) until the solution reached pH 11. The reaction mixture was stirred for 1 h, neutralized with Amberlite, filtered and concentrated under reduced pressure. The residue was dissolved in EtOAc/*t*-BuOH/H_2O (2 mL/2 mL/1 mL), palladium on activated charcoal (10% Pd, 30 mg) was added and the reaction mixture was stirred under a hydrogen atmosphere for 8 h. After complete consumption of the starting material, the reaction mixture was filtered and the crude product obtained after solvent removal was purified by reversed phase HPLC using a preparative Hypercarb column (From 0% to 30% MeCN in H2O in 30 min, flow rate 3 mL/min, t_R = 15.4 min) to afford **22** (5.9 mg, 0.01 mmol, 70%) as a white solid.

^{1}H NMR (700 MHz, D_2O) δ 5.20 (d, *J* = 1.6 Hz, 1H, Rha-H1'), 4.98 (d, *J* = 1.7 Hz, 1H, Rha-H1), 4.44 (d, *J* = 7.9 Hz, 1H, Gal-H1), 4.08 (dt, *J* = 3.5, 1.8 Hz, 2H), 3.99 (d, *J* = 3.5 Hz, 1H), 3.95 (ddd, *J* = 9.6, 5.1, 2.3 Hz, 2H), 3.86 – 3.76 (m, 3H), 3.76 – 3.66 (m, 5H), 3.63 (dd, *J* = 9.8, 7.9 Hz, 1H), 3.48 (dt, *J* = 31.6, 9.7 Hz, 2H), 3.01 (t, *J* = 7.6 Hz, 2H), 1.69 (dp, *J* = 15.3, 7.0 Hz, 4H), 1.47 (p, *J* = 7.6 Hz, 2H), 1.30 (d, *J* = 6.2 Hz, 3H), 1.27 (d, *J* = 6.3 Hz, 3H); ^{13}C NMR (176 MHz, D_2O) δ 102.57 (Gal-C1), 102.26 (Rha-C1), 100.78 (Rha-C1'), 79.82, 78.14, 75.10, 72.07, 71.98, 70.53, 70.05, 70.01, 69.86, 69.28, 69.16, 68.39, 60.84 (linker), 39.37 (linker), 28.17 (linker), 26.41 (linker), 22.09 (linker), 16.63 (Rha-CH_3), 16.60 (Rha-CH_3); HRMS (ESI) calculated for $C_{23}H_{43}NO_{14}$ $[M+H]^+$: 558.2756, found: 558.2791.

5-Aminopentyl *α*-L-rhamnopyranosyl-(1→2)-*α*-L-rhamnopyranosyl-(1→2)-*α*-L-rhamnopyranosyl-(1→3)-*β*-D-galactopyranoside (23)

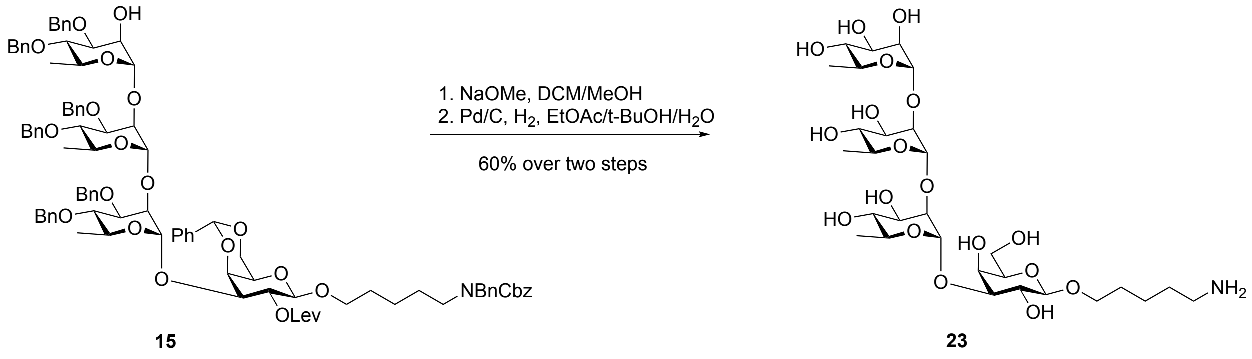

NaOMe was added slowly to a solution of **15** (16 mg, 0.0097 mmol) in MeOH/DCM = 1/1 (1 mL) until the solution reached pH 11. The reaction mixture was stirred for 1 h, neutralized with Amberlite, filtered and concentrated under reduced pressure. The residue was dissolved in EtOAc/*t*-BuOH/H_2O (2 mL/2 mL/1 mL), palladium on activated charcoal (10% Pd, 30 mg) was added and the reaction mixture was stirred under a hydrogen atmosphere for 8 h. After complete consumption of the starting material, the reaction mixture was filtered and the crude product obtained after solvent removal was purified by reversed phase HPLC using a preparative Hypercarb column (From 0% to 30% MeCN in H_2O in 30 min, flow rate 3 mL/min, t_R = 18.1 min) to afford **23** (4.1 mg, 0.006 mmol, 60%) as a white solid.

^{1}H NMR (600 MHz, D_2O) δ 5.07 (d, *J* = 1.7 Hz, 1H, Rha-H1’’), 5.03 (d, *J* = 1.8 Hz, 1H, Rha-H1’), 4.88 (d, *J* = 1.8 Hz, 1H, Rha-H1), 4.34 (dd, *J* = 7.9, 1.8 Hz, 1H, Gal-H1), 3.99 (ddq, *J* = 7.0, 3.5, 1.8 Hz, 3H), 3.91 – 3.82 (m, 3H), 3.80 (ddd, *J* = 9.8, 3.4, 1.8 Hz, 1H), 3.75 – 3.57 (m, 9H), 3.53 (ddd, *J* = 9.8, 7.9, 1.8 Hz, 1H), 3.44 – 3.32 (m, 3H), 2.95 – 2.87 (m, 2H), 1.65 – 1.52 (m, 4H), 1.43 – 1.34 (m, 2H), 1.26 – 1.14 (m, 9H); ^{13}C NMR (151 MHz, D_2O) δ 102.53 (Gal-C1), 102.24 (Rha-C1), 100.81 (Rha-C1’), 100.78 (Rha-C1’’), 79.86, 78.21, 77.86, 75.07, 72.03, 72.01, 71.93, 70.49, 70.01, 69.98, 69.91, 69.77, 69.26, 69.12, 68.35, 60.80 (linker), 39.32 (linker), 28.13 (linker), 26.37 (linker), 22.05 (linker), 16.58 (Rha-CH_3, overlapped), 16.55 (Rha-CH_3); HRMS (ESI) calculated for $C_{29}H_{53}NO_{18}$ $[M+H]^+$: 704.3335, found: 704.3344.

5-Aminopentyl *α*-D-galactopyranosyl-(1→2)-*α*-L-rhamnopyranosyl-(1→2)-*α*-L-rhamnopyranosyl-(1→2)-*α*-L-rhamnopyranosyl-(1→3)-*β*-D-galactopyranoside (24)

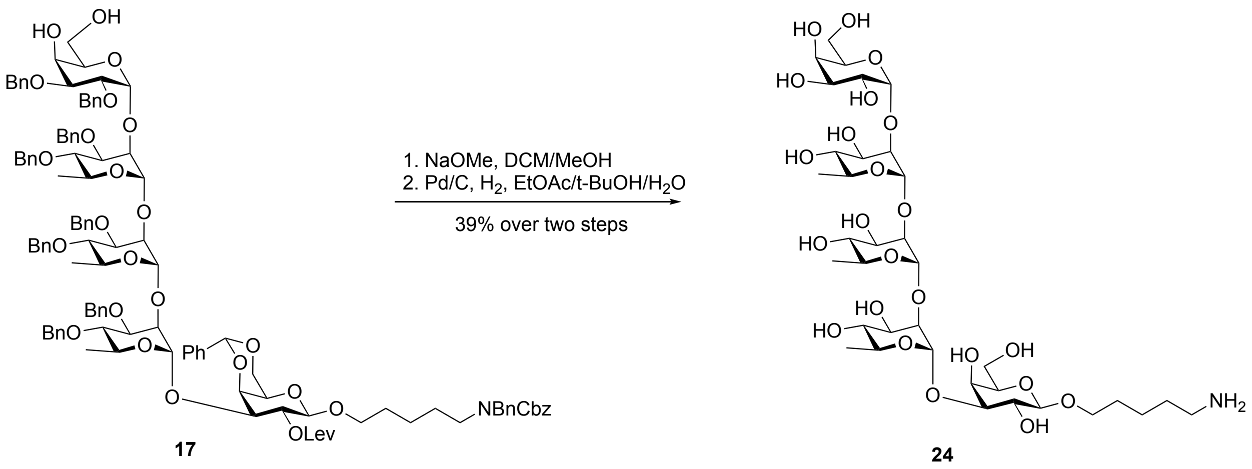

NaOMe was added slowly to a solution of **17** (15 mg, 0.0075 mmol) in MeOH/DCM = 1/1 (1 mL) until the solution reached pH 11. The reaction mixture was stirred for 1 h, neutralized with Amberlite, filtered and concentrated under reduced pressure. The residue was dissolved in EtOAc/*t*-BuOH/H_2O (2 mL/2 mL/1 mL), palladium on activated charcoal (10% Pd, 30 mg) was added and the reaction mixture was stirred under a hydrogen atmosphere for 8 h. After complete consumption of the starting material, the reaction mixture was filtered and the crude product obtained after solvent removal was purified by reversed phase HPLC using a preparative Hypercarb column (From 0% to 30% MeCN in H_2O in 30 min, flow rate 3 mL/min, t_R = 18.7 min) to afford **24** (2.5 mg, 0.003 mmol, 39%) as a white solid.

^{1}H NMR (600 MHz, D_2O) δ 5.16 (s, 1H, Rha-H1''), 5.14 (s, 1H, Rha-H1'), 5.11 (s, 1H, Rha-H1), 5.05 (d, *J* = 4.0 Hz, 1H, Gal-H1'), 4.43 (d, *J* = 7.8 Hz, 1H, Gal-H1), 4.25 (t, *J* = 6.4 Hz, 1H), 4.12 (t, *J* = 2.3 Hz, 2H), 4.07 (d, *J* = 3.3 Hz, 1H), 4.00 (dd, *J* = 13.3, 3.5 Hz, 2H), 3.97 – 3.86 (m, 5H), 3.84 – 3.66 (m, 11H), 3.62 (dd, *J* = 10.3, 7.5 Hz, 1H), 3.55 – 3.45 (m, 3H), 3.00 (t, *J* = 7.5 Hz, 2H), 1.68 (dp, *J* = 13.3, 7.2, 6.7 Hz, 4H), 1.46 (p, *J* = 7.6, 6.9 Hz, 2H), 1.28 (dt, *J* = 8.6, 4.5 Hz, 9H); ^{13}C NMR (151 MHz, D_2O) δ 102.53 (Gal-C1), 100.79 (Rha-C1''), 100.74 (Rha-C1'), 99.48 (Rha-C1), 97.60 (Gal-C1'), 79.84, 78.47, 77.91, 75.89, 75.07, 72.04, 72.00, 71.84, 70.92, 70.49, 70.00, 69.90, 69.72, 69.53, 69.38, 69.26, 69.09, 68.35, 68.20, 60.86 (linker), 39.32 (linker), 28.13 (linker), 26.38 (linker), 22.05 (linker), 16.58 (Rha-CH_3), 16.54 (Rha-CH_3), 16.51 (Rha-CH_3); HRMS (ESI) calculated for $C_{35}H_{63}NO_{23}$ $[M+H]^+$: 866.3864, found: 866.3925.

3. NMR spectra

^{1}H NMR ($CDCl_3$, 400 MHz) of compound **5**

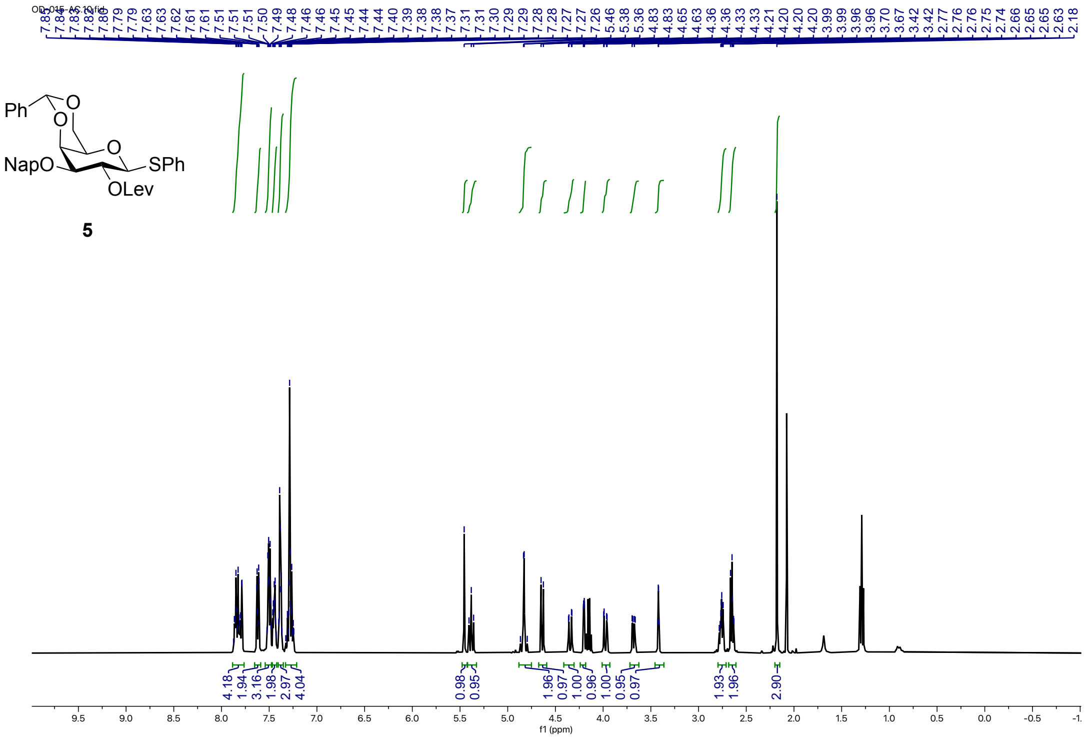

^{13}C NMR ($CDCl_3$, 101 MHz) of compound **5**

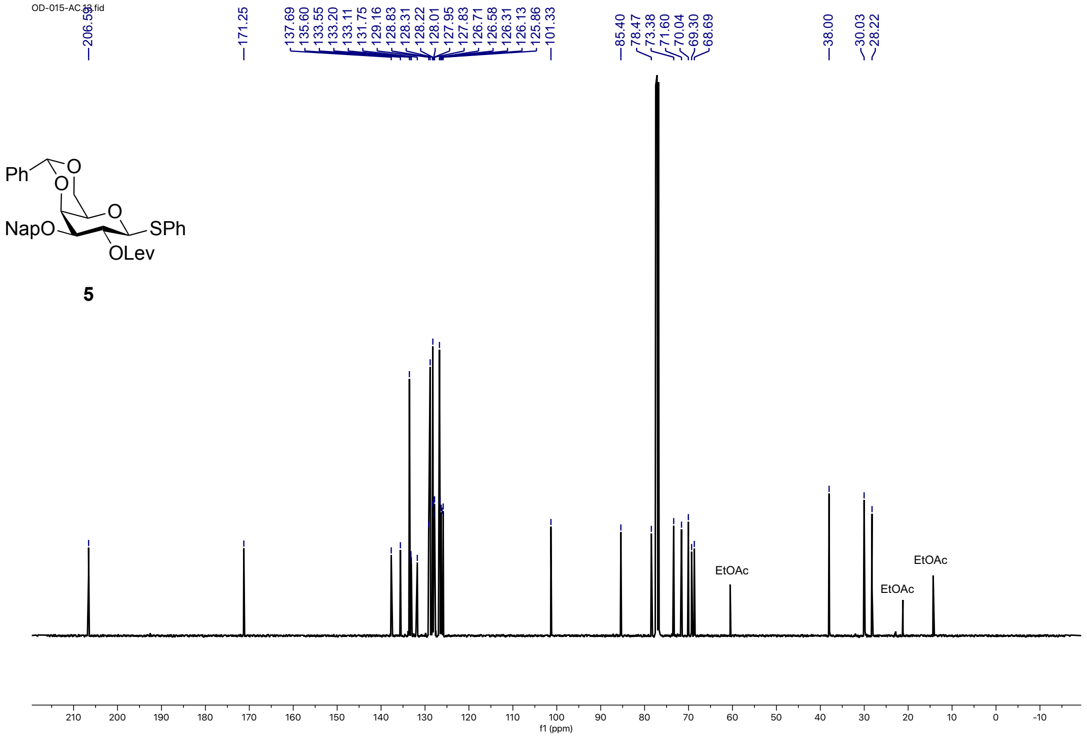

^{1}H-^{1}H COSY ($CDCl_3$, 400 MHz) of compound **5**

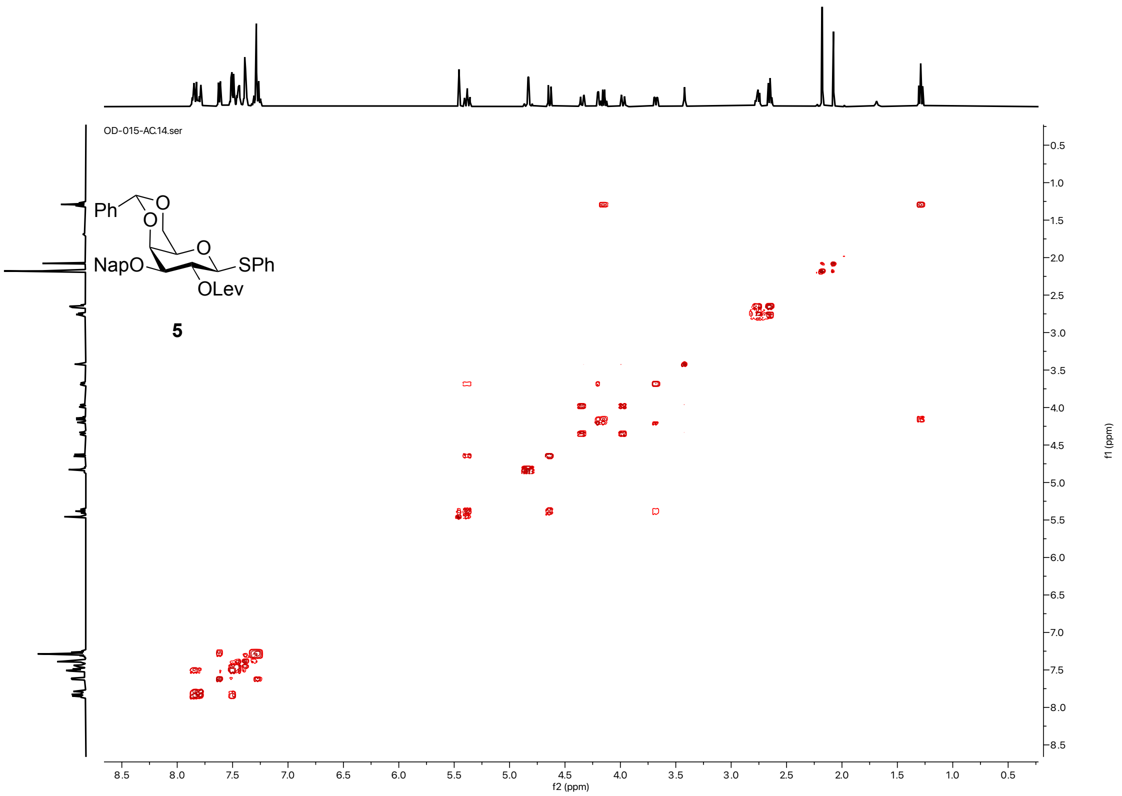

^{1}H-^{13}C HSQC (Decoupling, $CDCl_3$, 400 MHz) of compound **5**

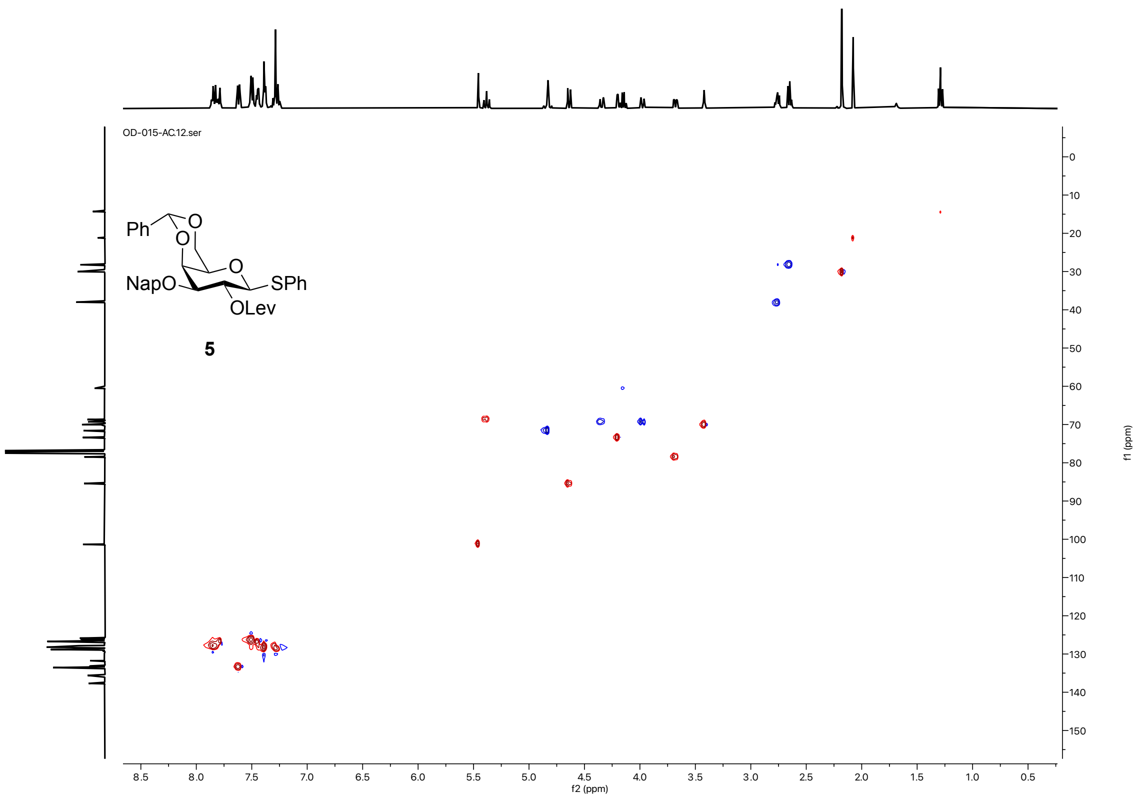

^{1}H-^{13}C HSQC (No decoupling, $CDCl_3$, 400 MHz) of compound **5**

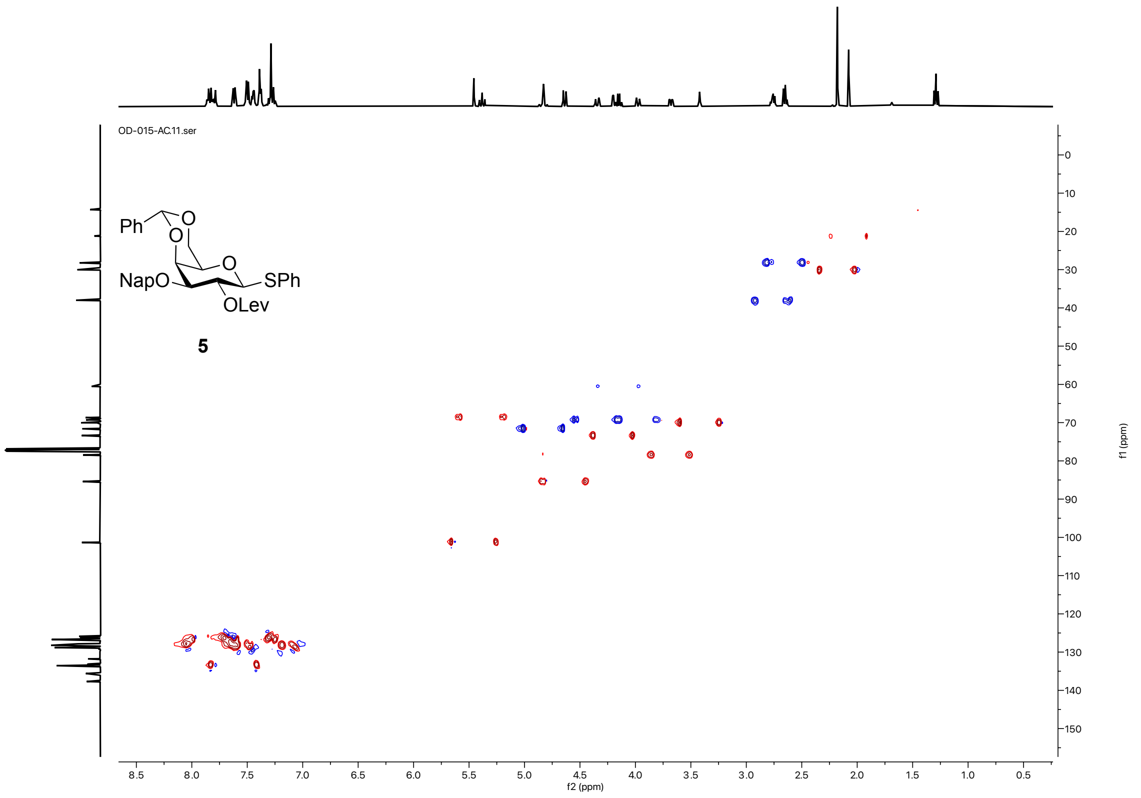

^{1}H NMR ($CDCl_3$, 400 MHz) of compound **7**

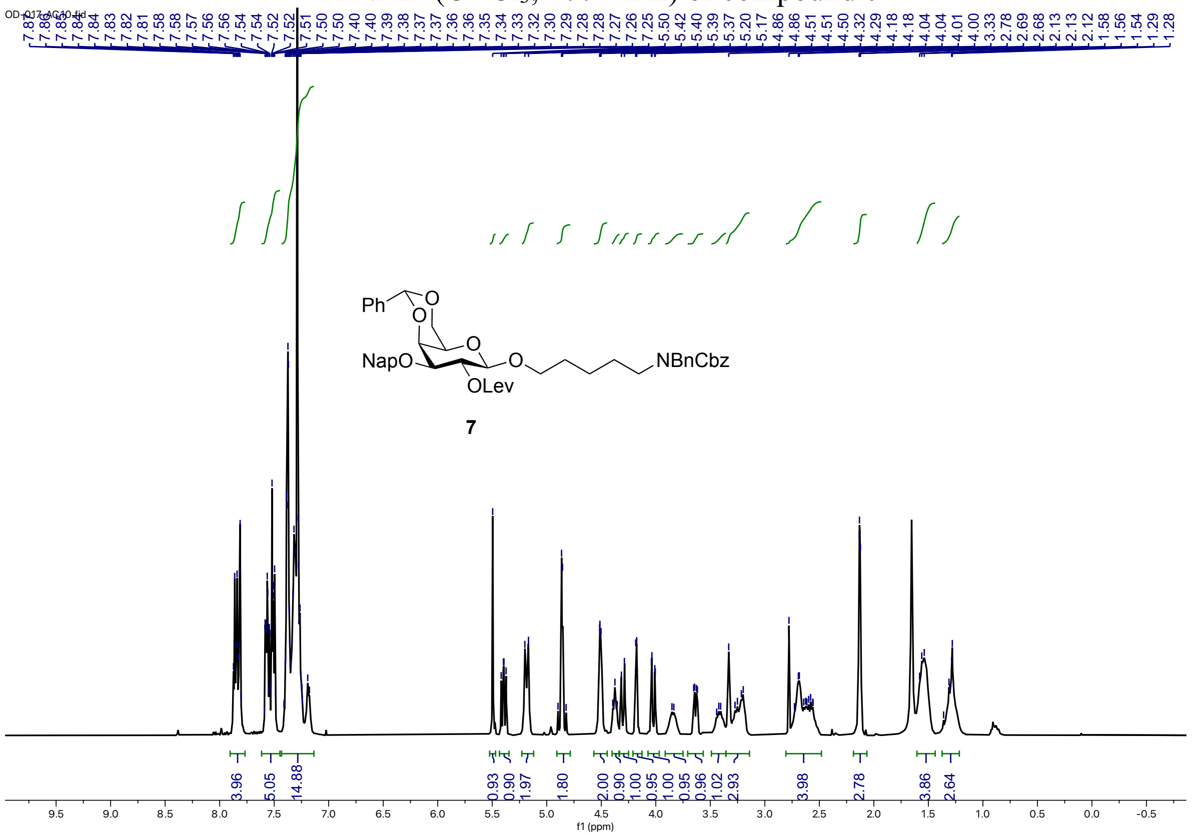

^{13}C NMR (CDCl3, 101 MHz) of compound **7**

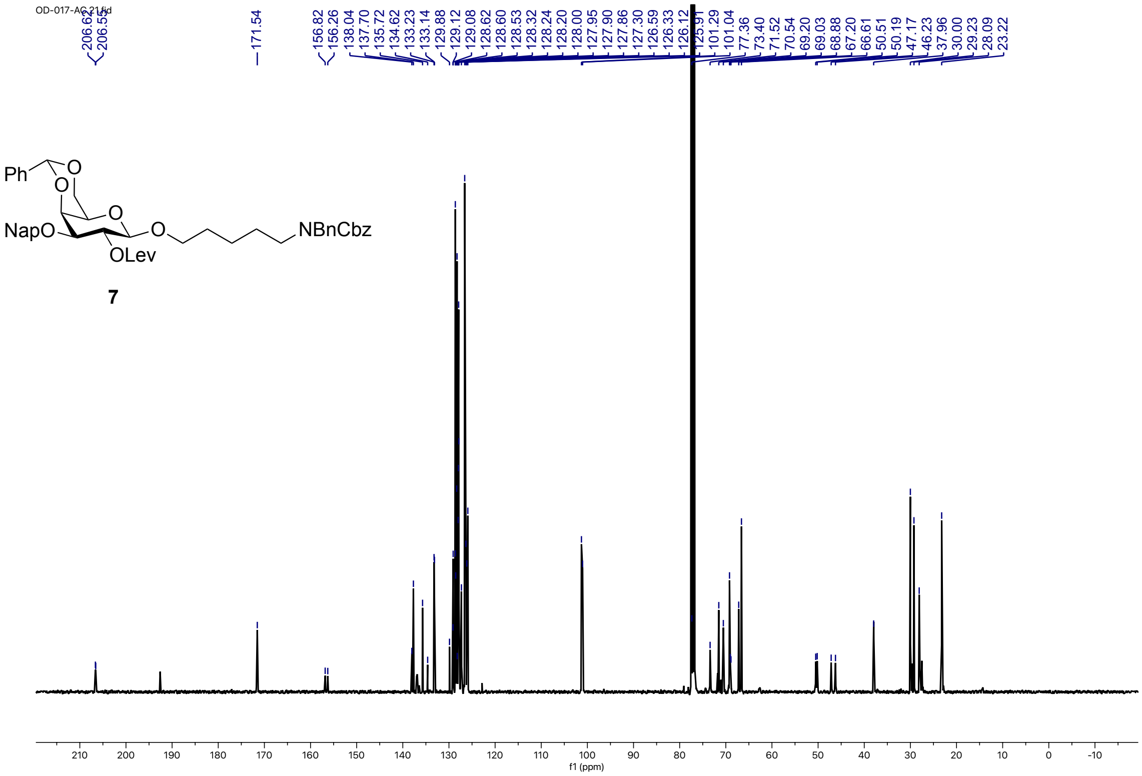

^{1}H-^{1}H COSY (CDCl3, 400 MHz) of compound **7**

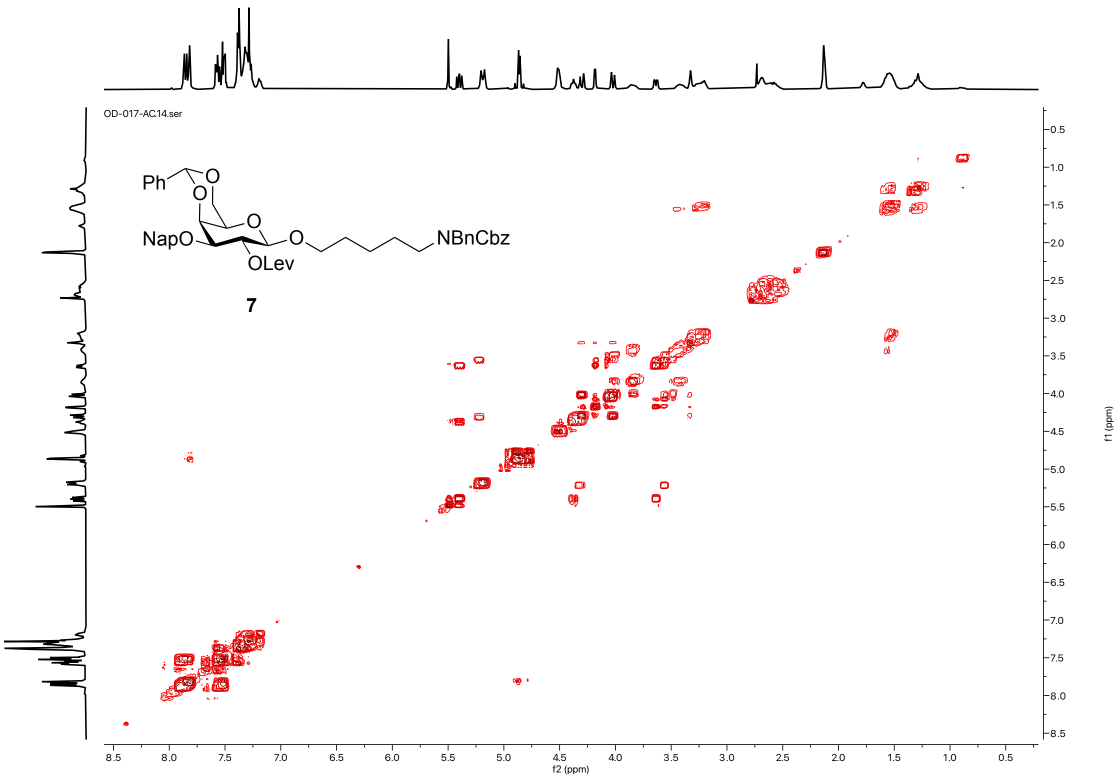

^{1}H-^{13}C HSQC (Decoupling, $CDCl_3$, 400 MHz) of compound **7**

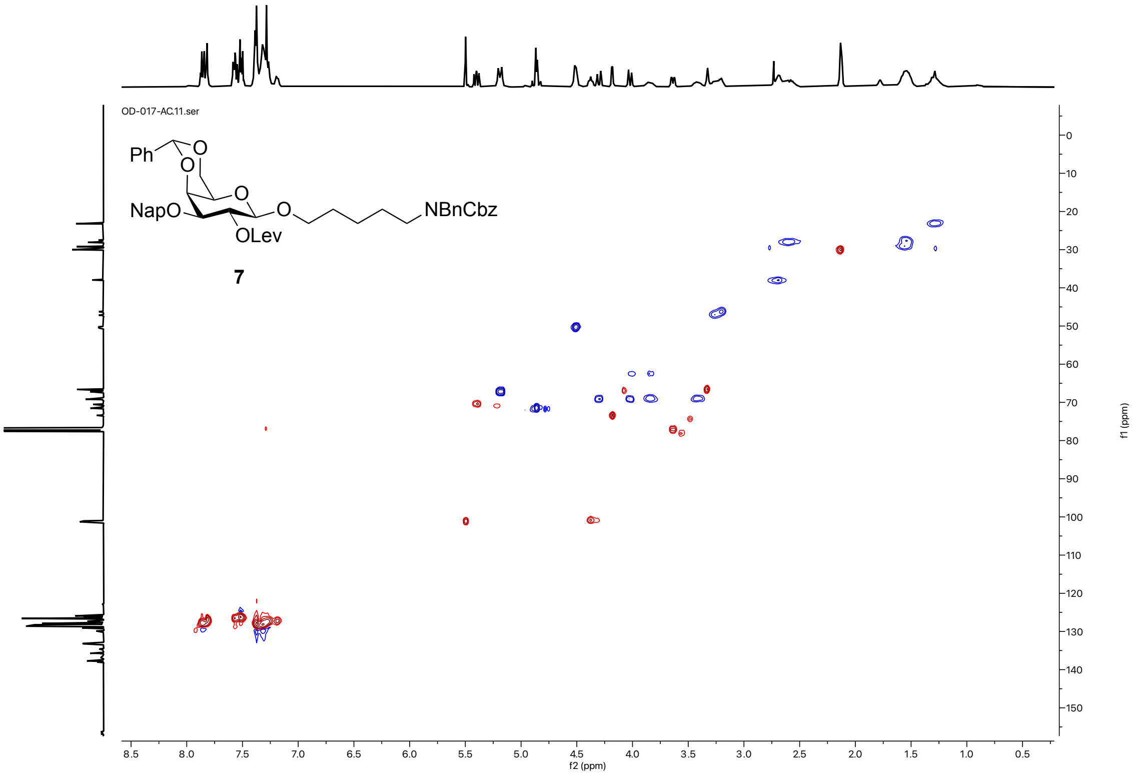

^{1}H-^{13}C HSQC (No decoupling, $CDCl_3$, 400 MHz) of compound **7**

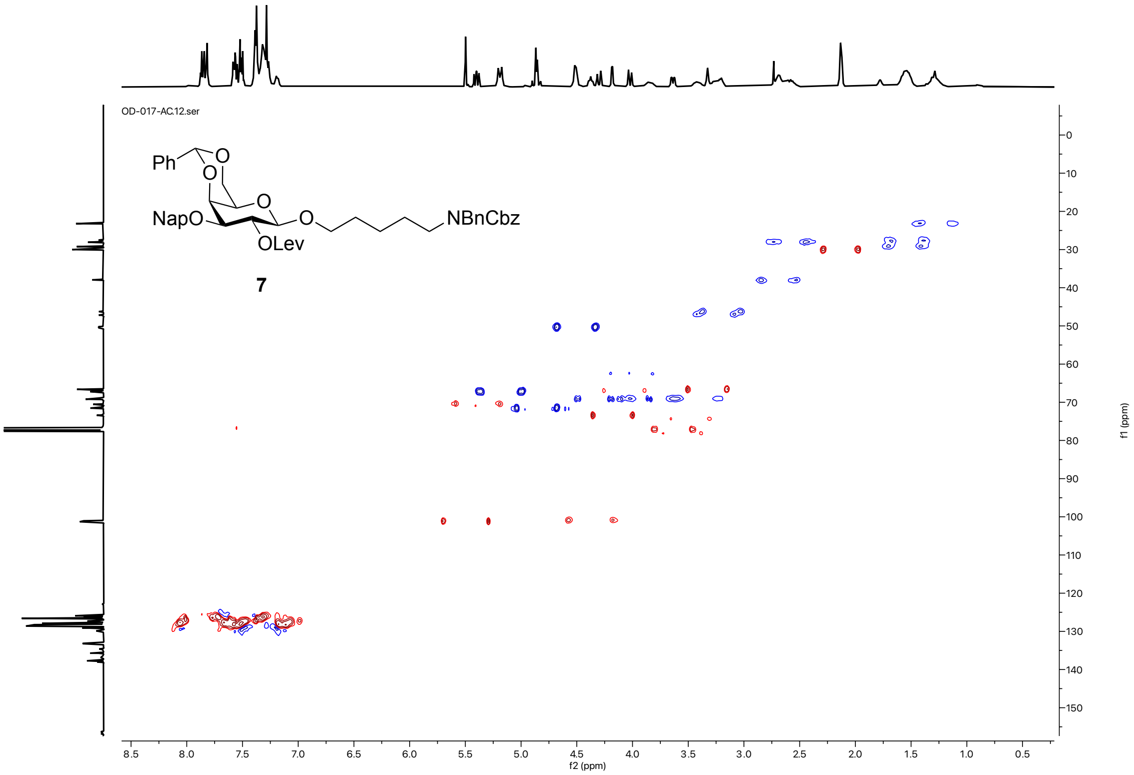

^{1}H NMR ($CDCl_3$, 400 MHz) of compound **1**

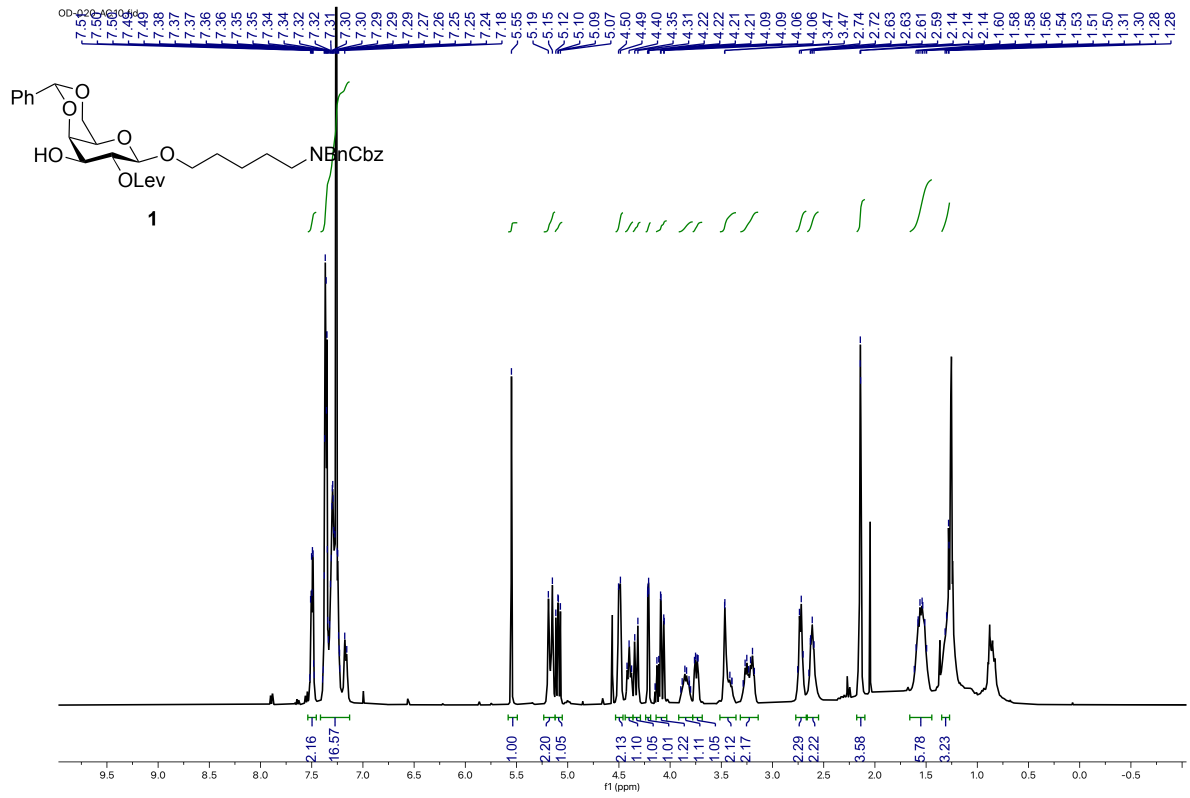

^{13}C NMR ($CDCl_3$, 101 MHz) of compound **1**

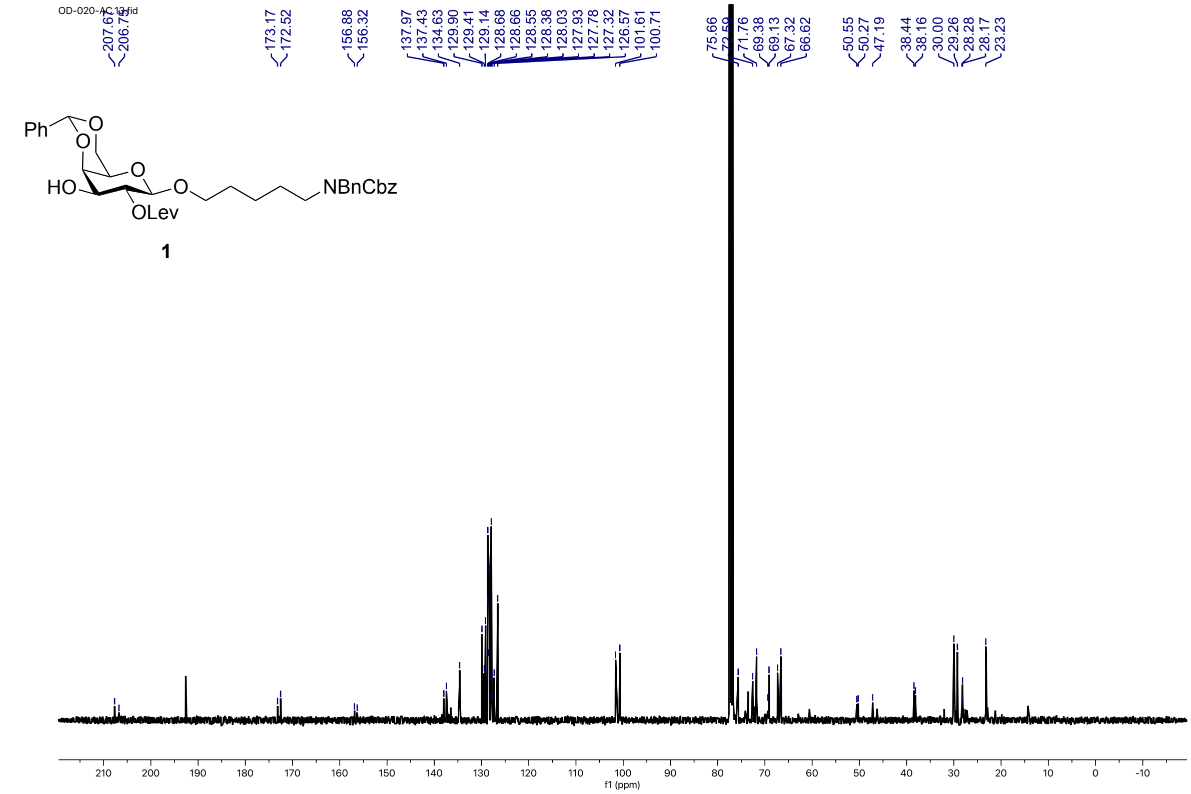

^1H-^1H COSY ($CDCl_3$, 400 MHz) of compound **1**

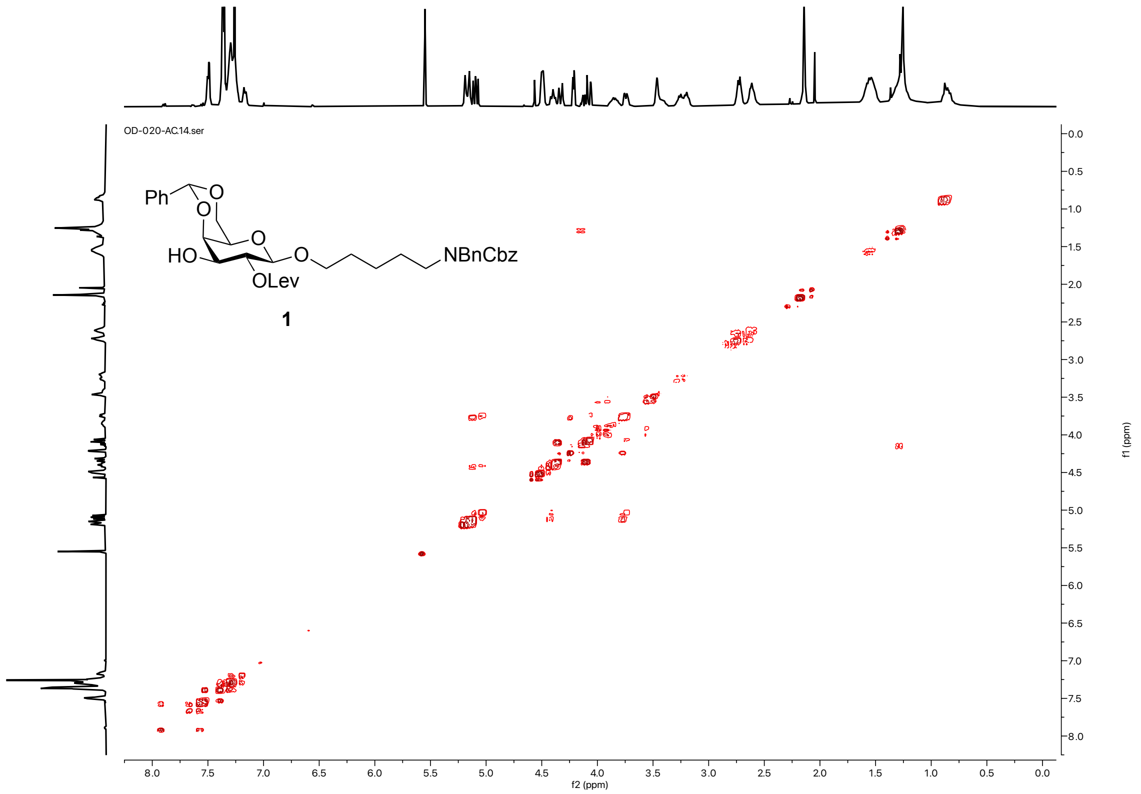

^1H-^{13}C HSQC (Decoupling, $CDCl_3$, 400 MHz) of compound **1**

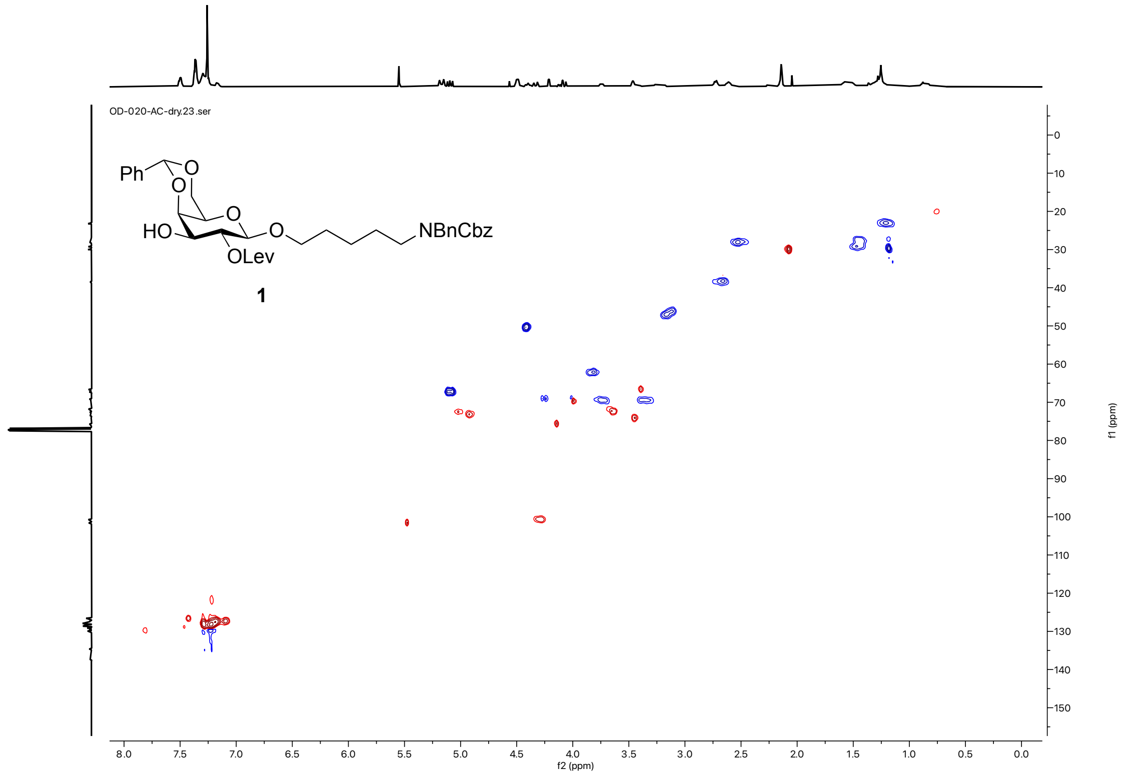

^{1}H-^{13}C HSQC (No decoupling, $CDCl_3$, 400 MHz) of compound **1**

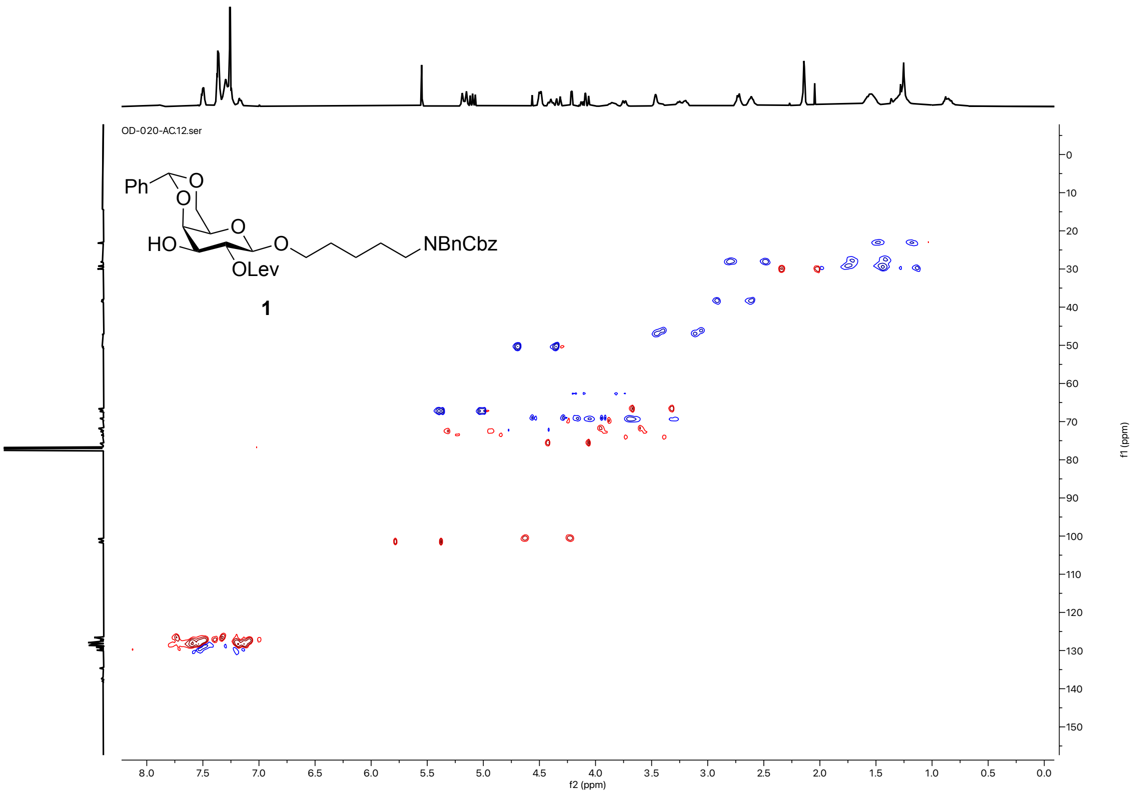

^{1}H NMR ($CDCl_3$, 400 MHz) of compound **9**

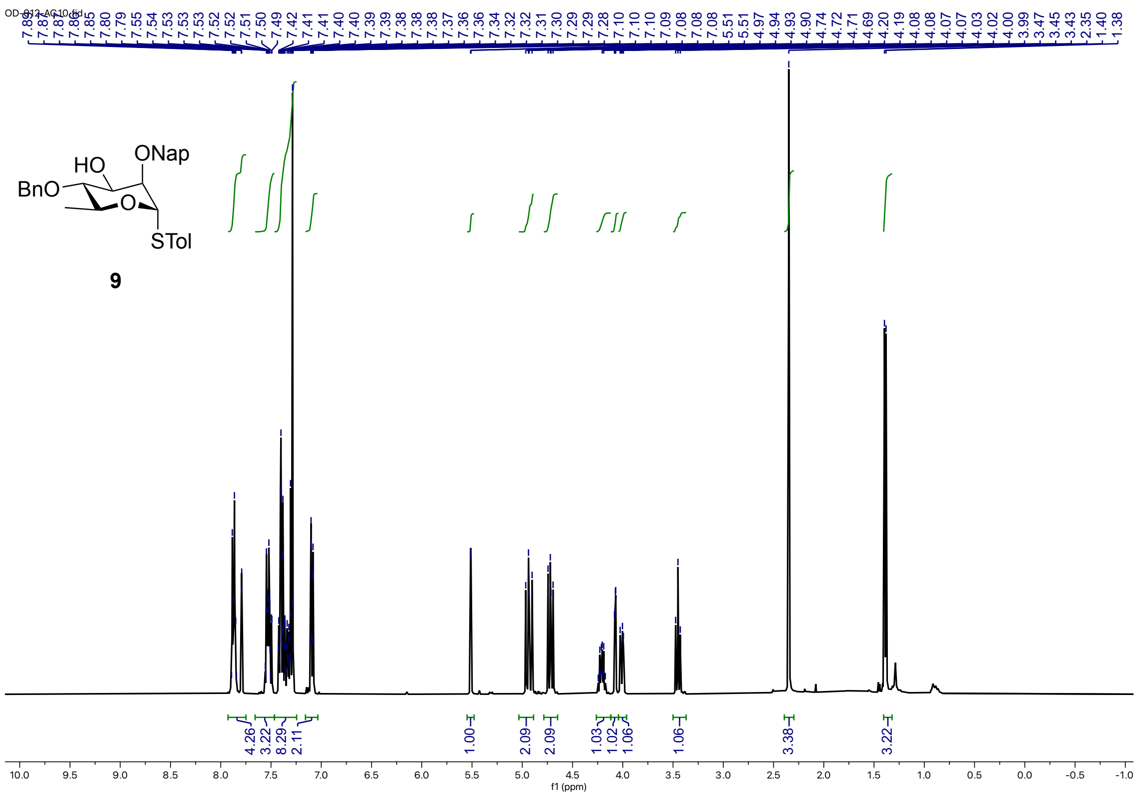

^{13}C NMR ($CDCl_3$, 101 MHz) of compound **9**

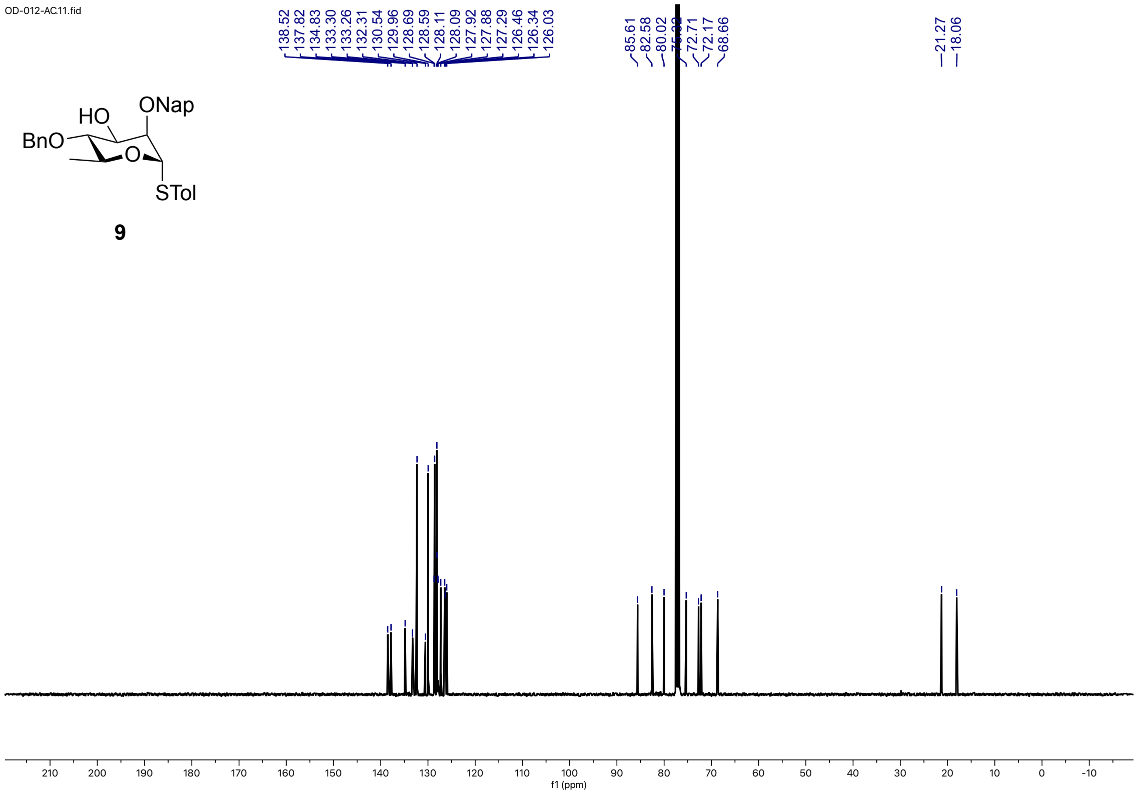

^{1}H-^{1}H COSY ($CDCl_3$, 400 MHz) of compound **9**

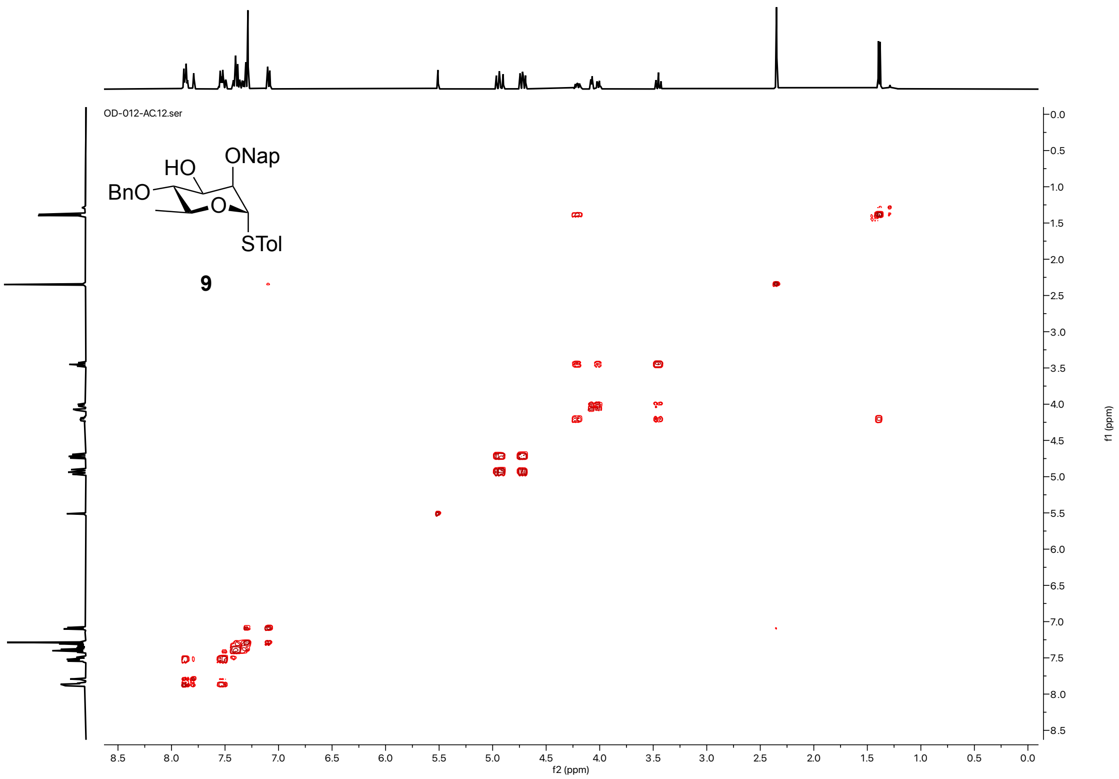

^{1}H-^{13}C HSQC (Decoupling, $CDCl_3$, 400 MHz) of compound **9**

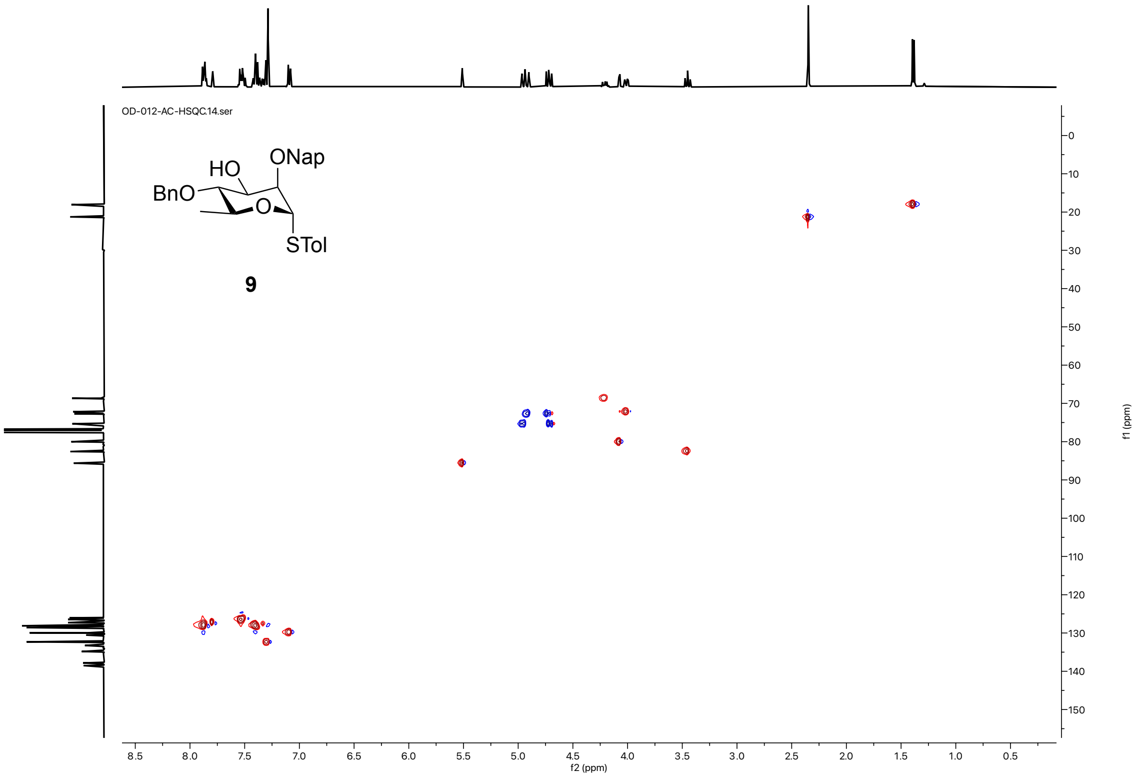

^{1}H-^{13}C HSQC (No decoupling, $CDCl_3$, 400 MHz) of compound **9**

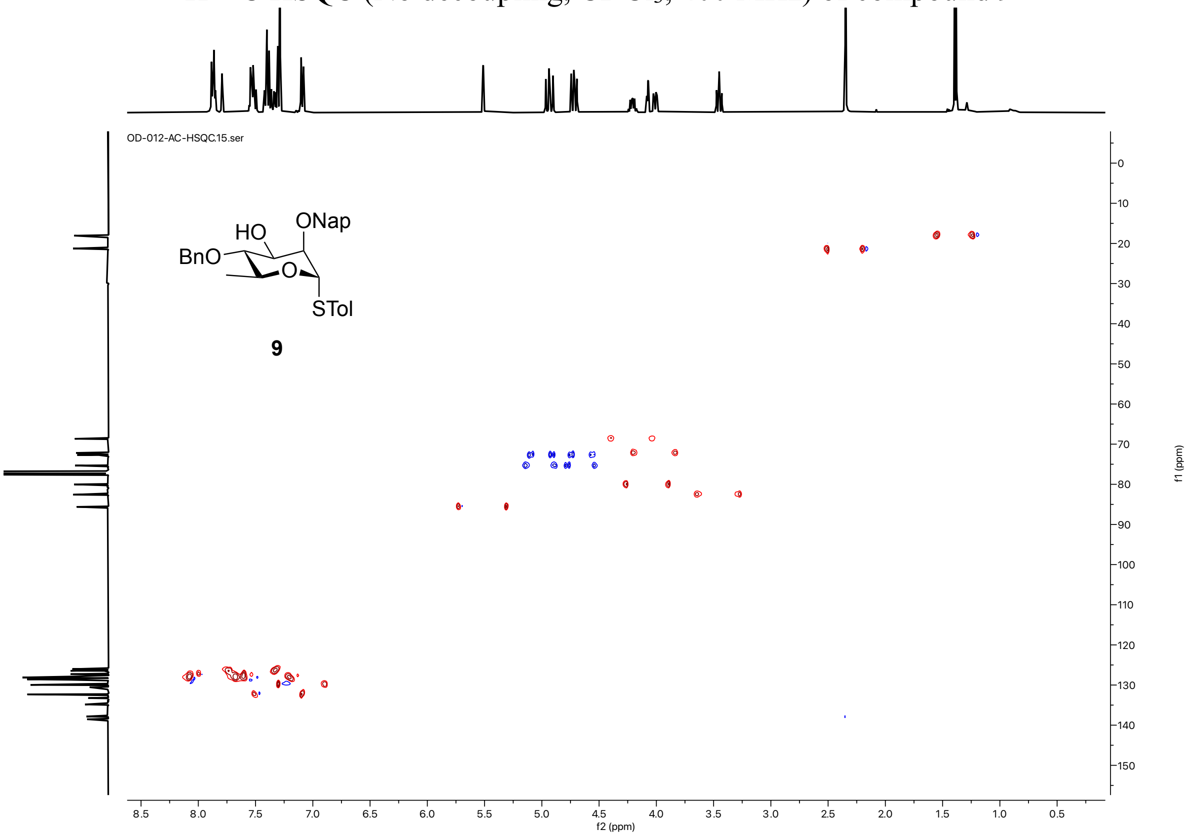

^{1}H NMR ($CDCl_3$, 400 MHz) of compound **4**

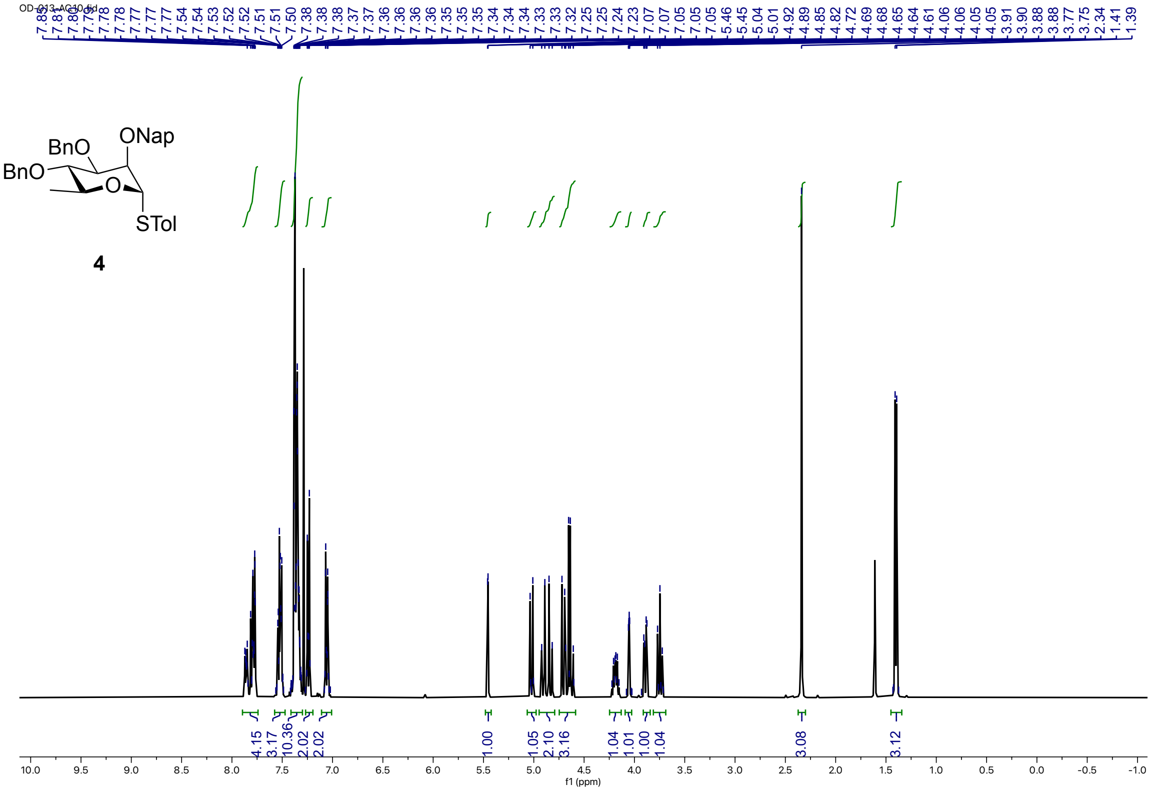

^{13}C NMR ($CDCl_3$, 101 MHz) of compound **4**

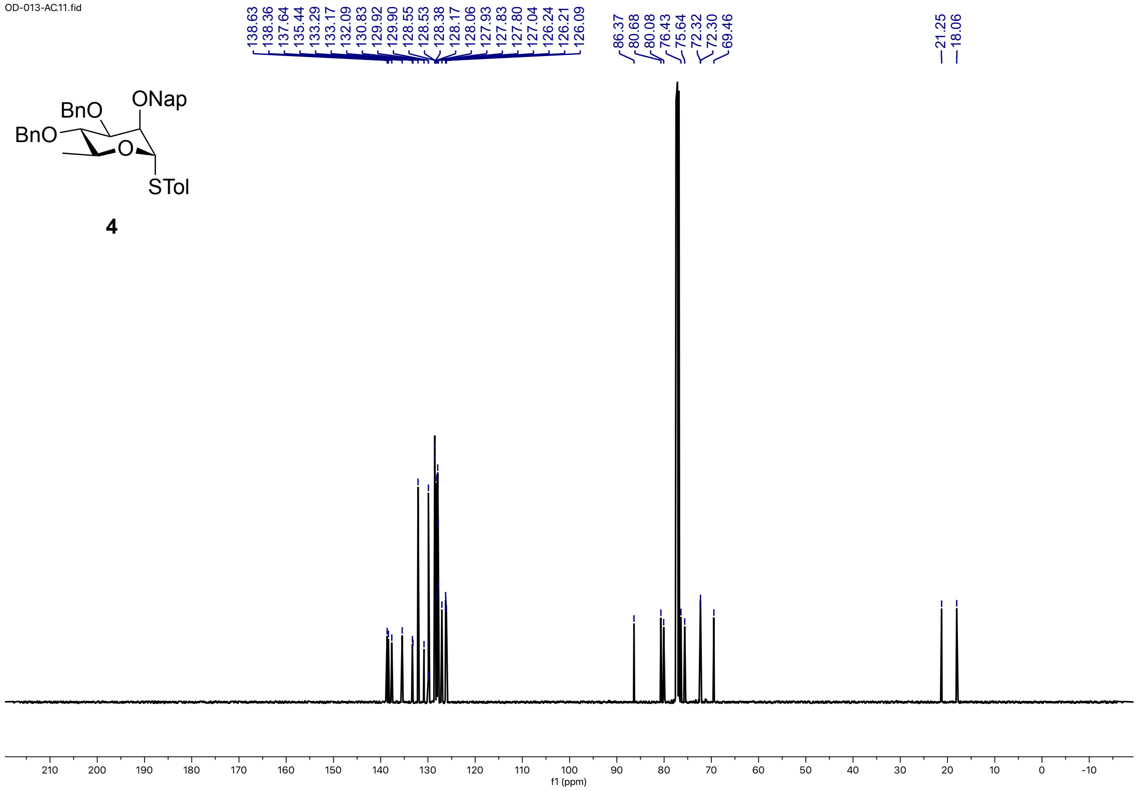

^{1}H-^{1}H COSY ($CDCl_3$, 400 MHz) of compound **4**

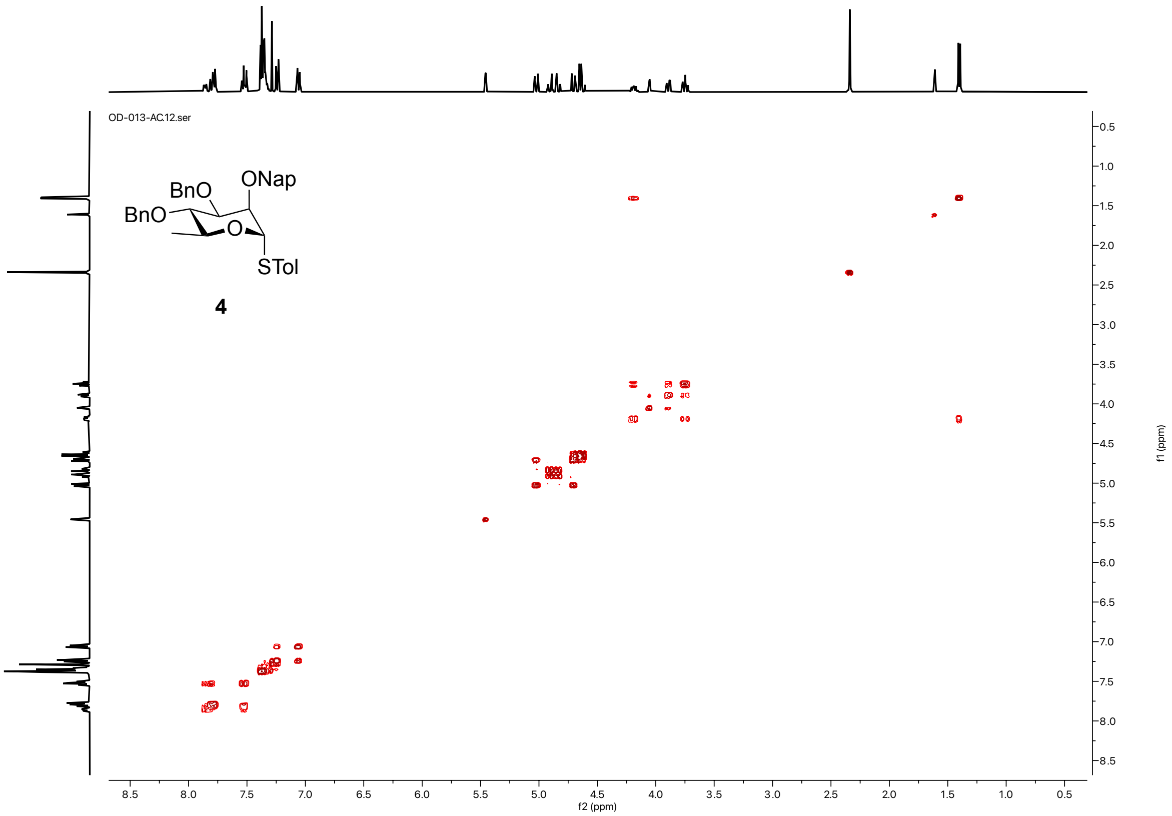

^{1}H-^{13}C HSQC (Decoupling, $CDCl_3$, 400 MHz) of compound **4**

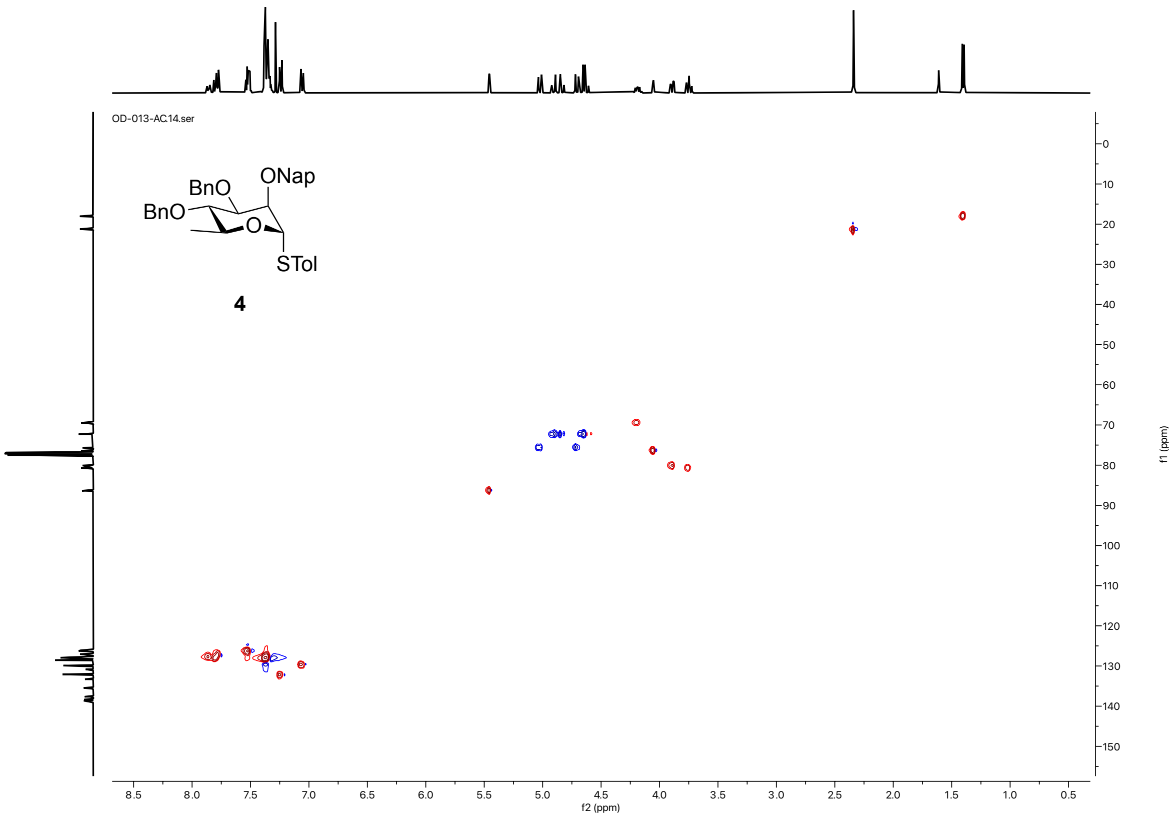

^{1}H-^{13}C HSQC (No decoupling, $CDCl_3$, 400 MHz) of compound **4**

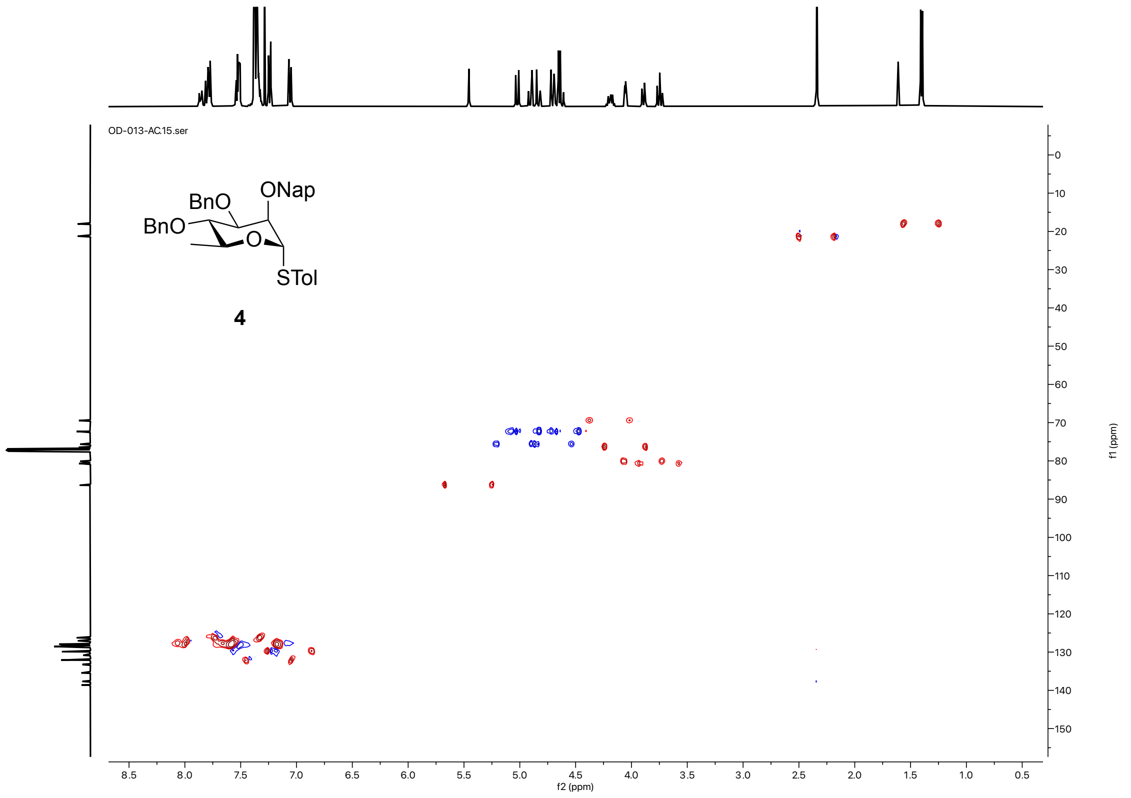

^{1}H NMR ($CDCl_3$, 400 MHz) of compound **10**

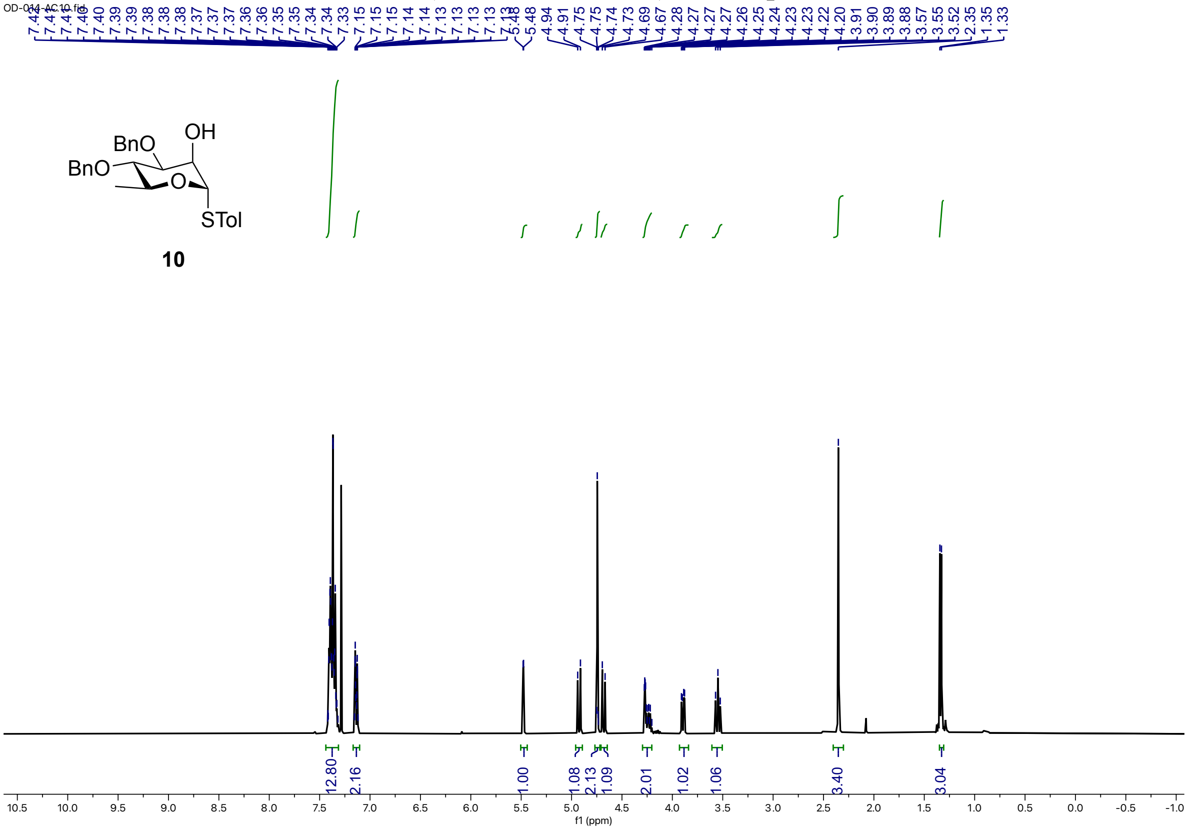

^{13}C NMR ($CDCl_3$, 101 MHz) of compound **10**

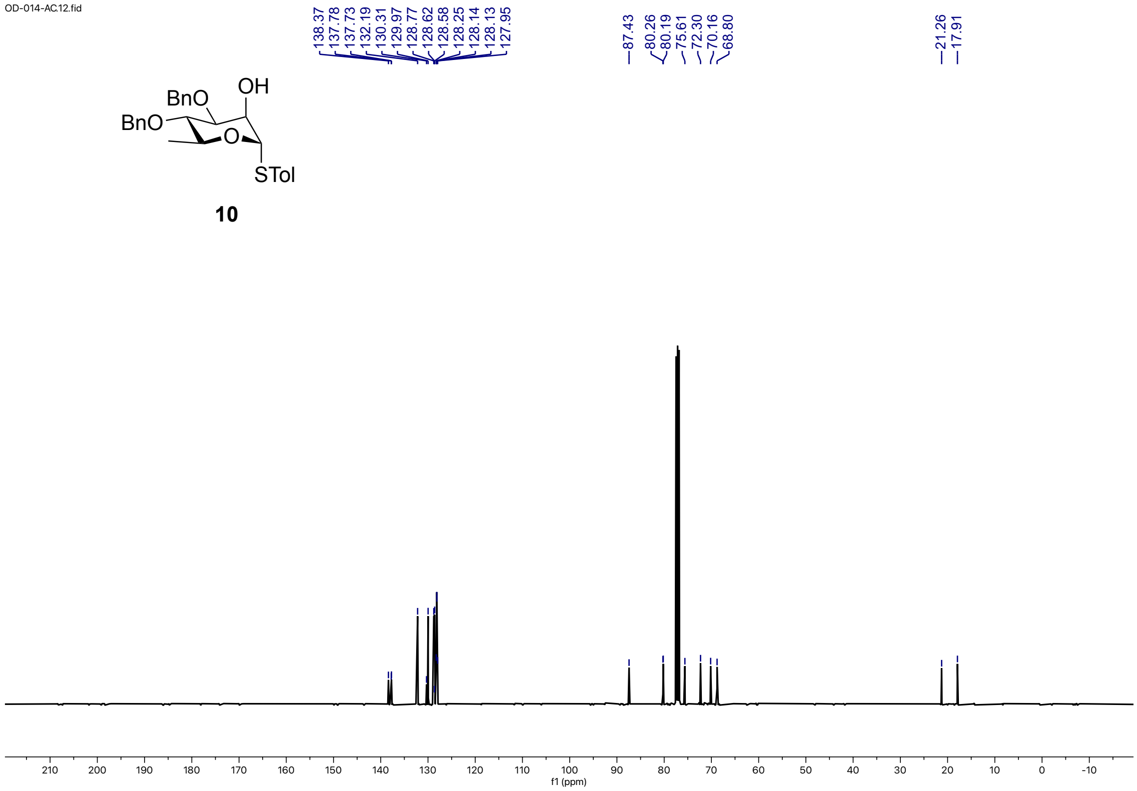

^1H-^1H COSY ($CDCl_3$, 400 MHz) of compound **10**

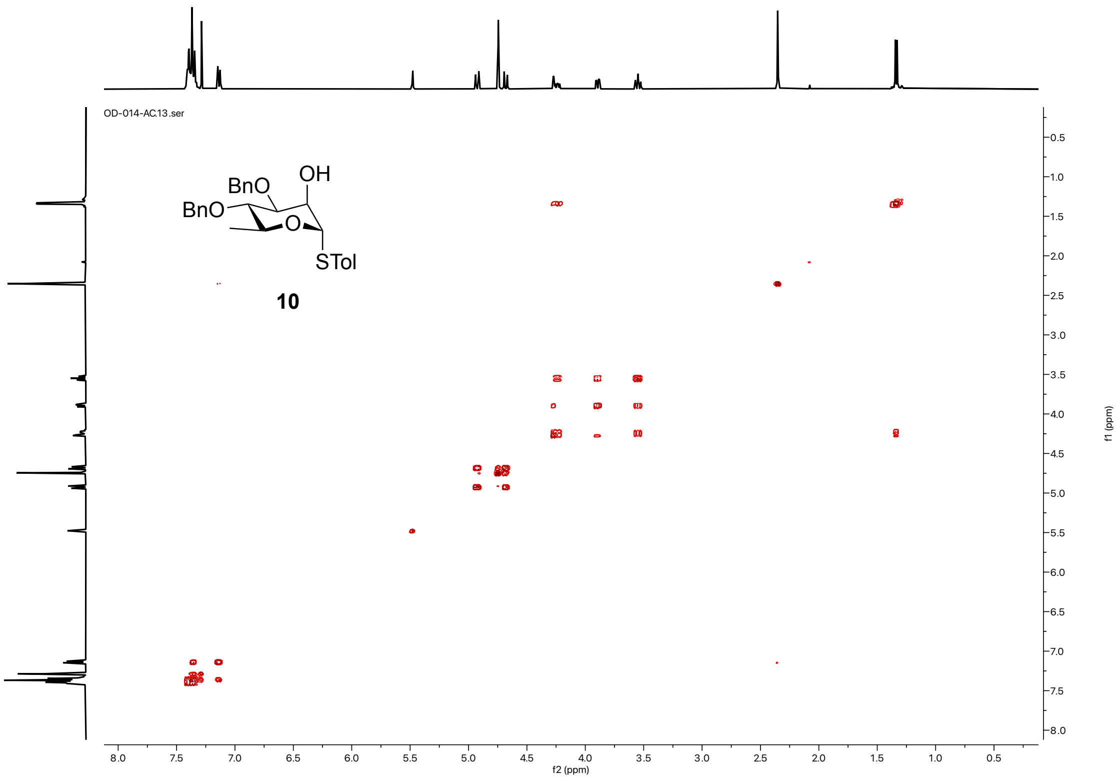

^{1}H-^{13}C HSQC (Decoupling, $CDCl_3$, 400 MHz) of compound **10**

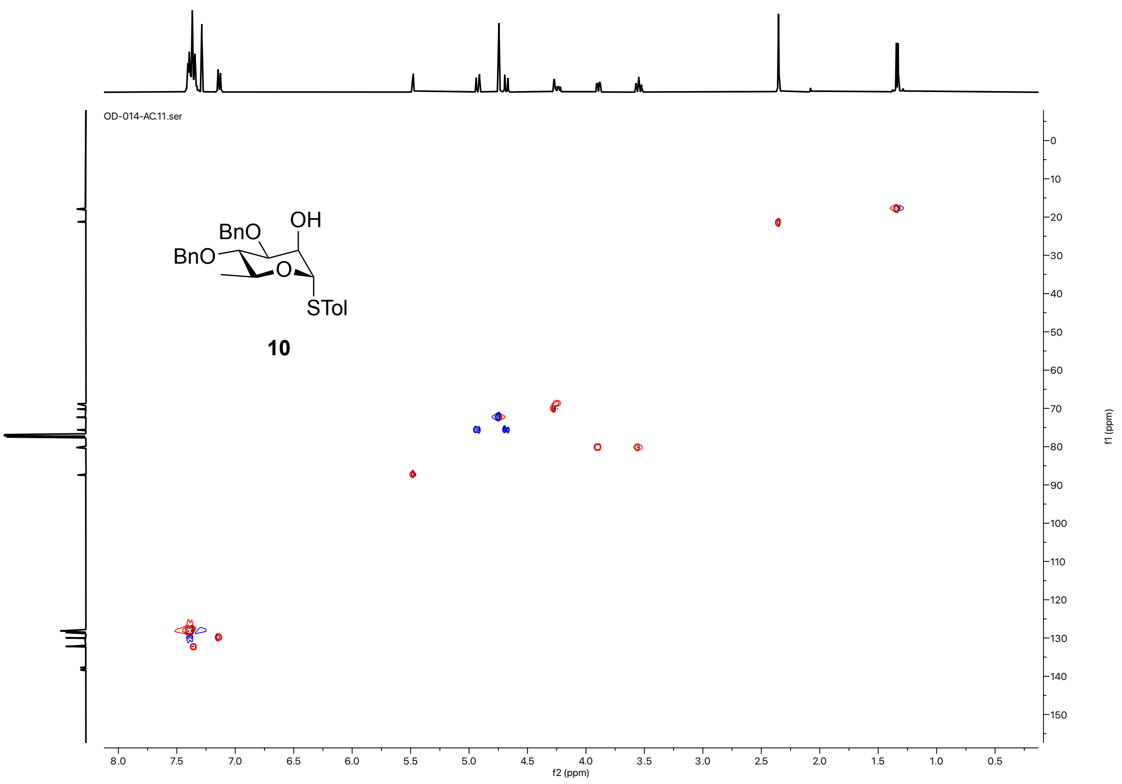

^{1}H NMR ($CDCl_3$, 400 MHz) of compound **2**

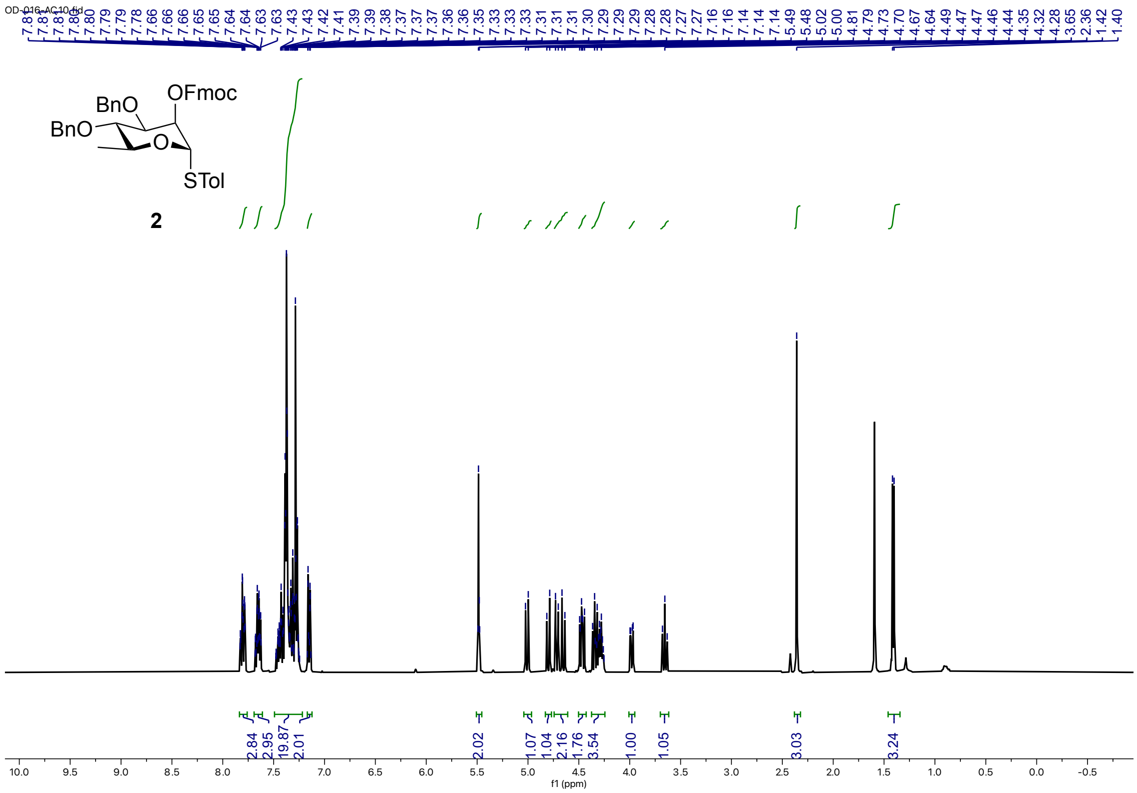

^{13}C NMR ($CDCl_3$, 101 MHz) of compound **2**

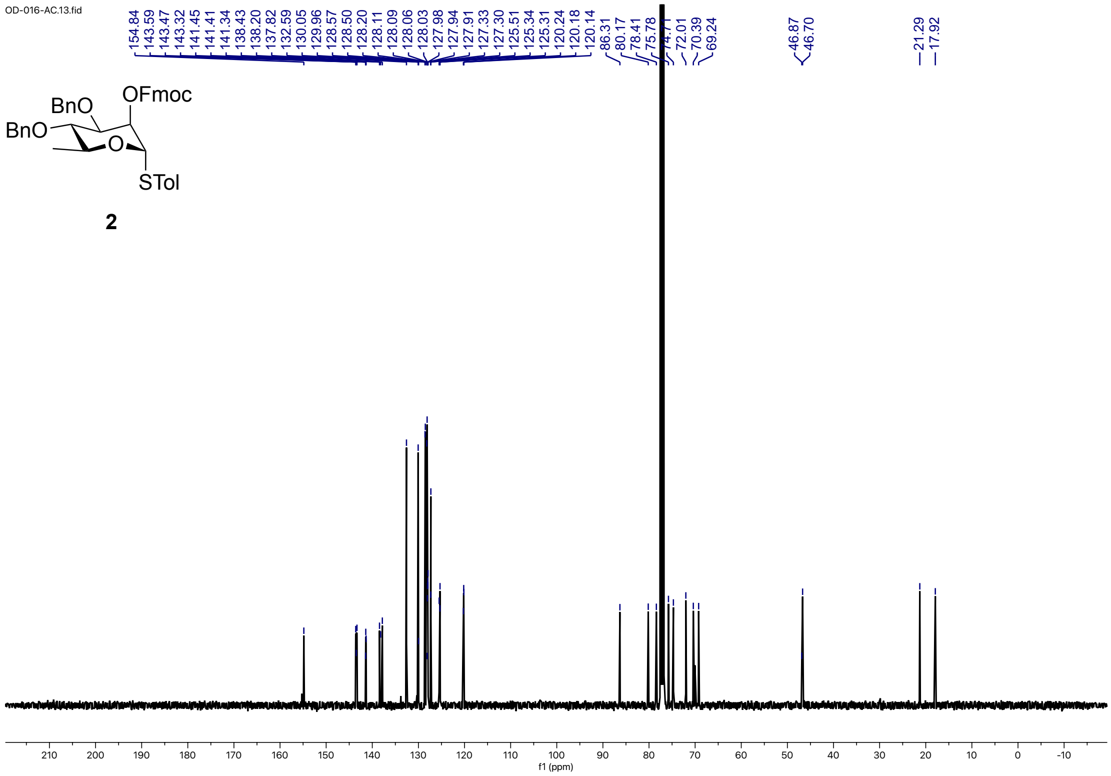

^{1}H-^{1}H COSY ($CDCl_3$, 400 MHz) of compound **2**

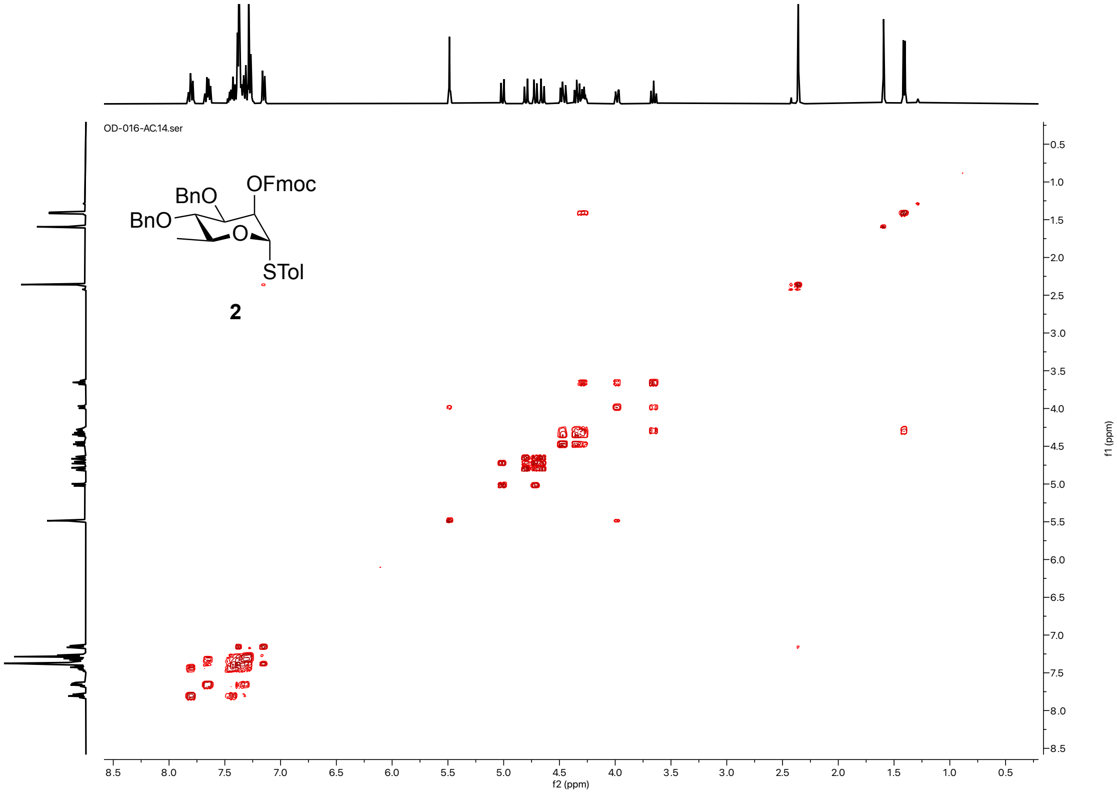

^{1}H-^{13}C HSQC (Decoupling, $CDCl_3$, 400 MHz) of compound **2**

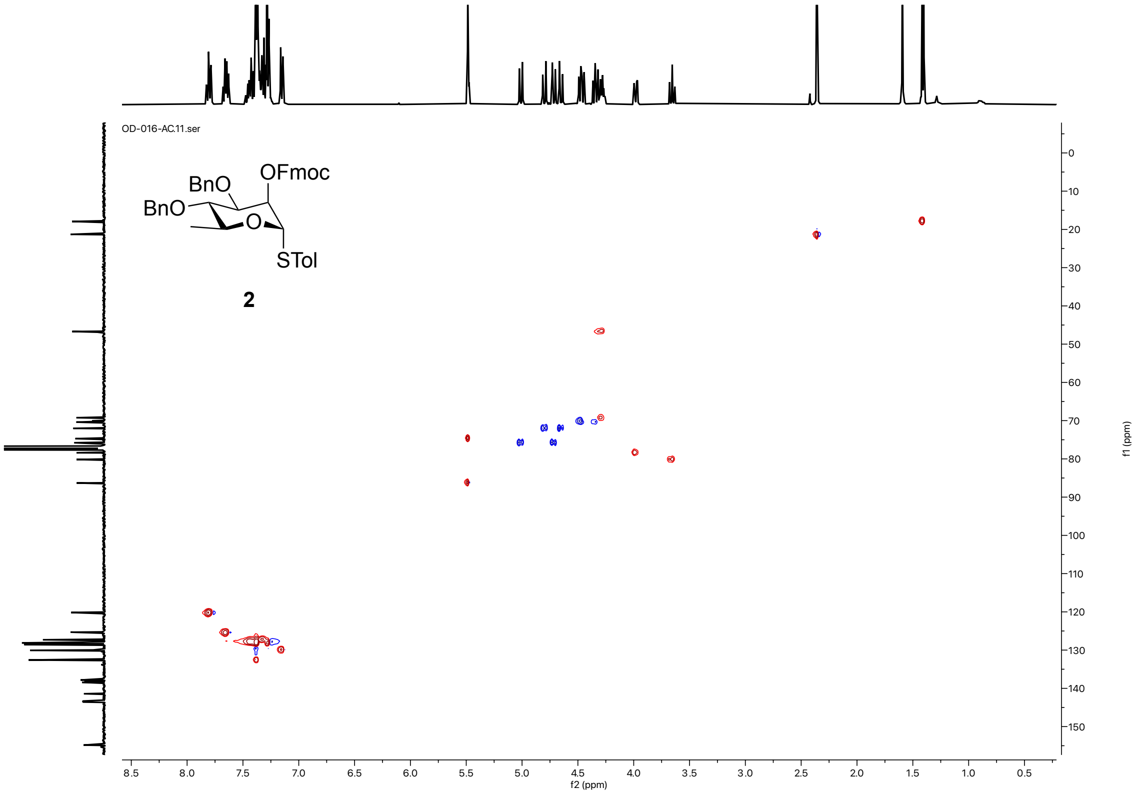

^{1}H-^{13}C HSQC (No decoupling, $CDCl_3$, 400 MHz) of compound **2**

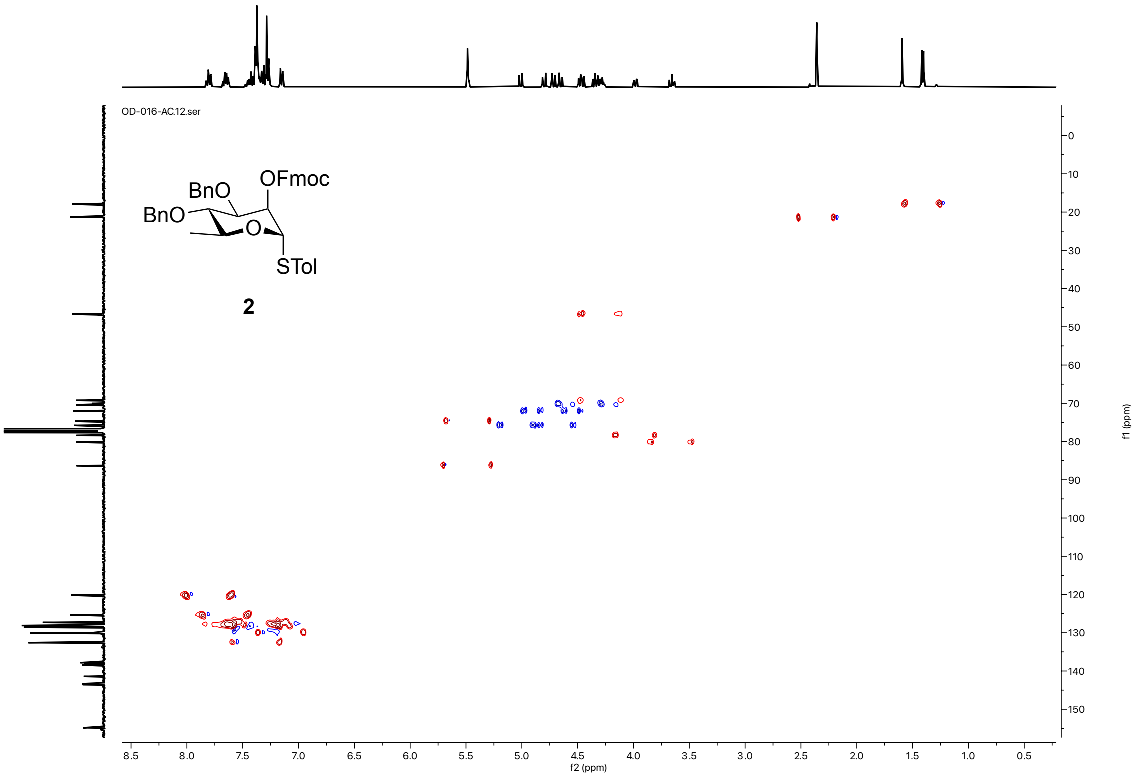

^{1}H NMR ($CDCl_3$, 400 MHz) of compound **12**

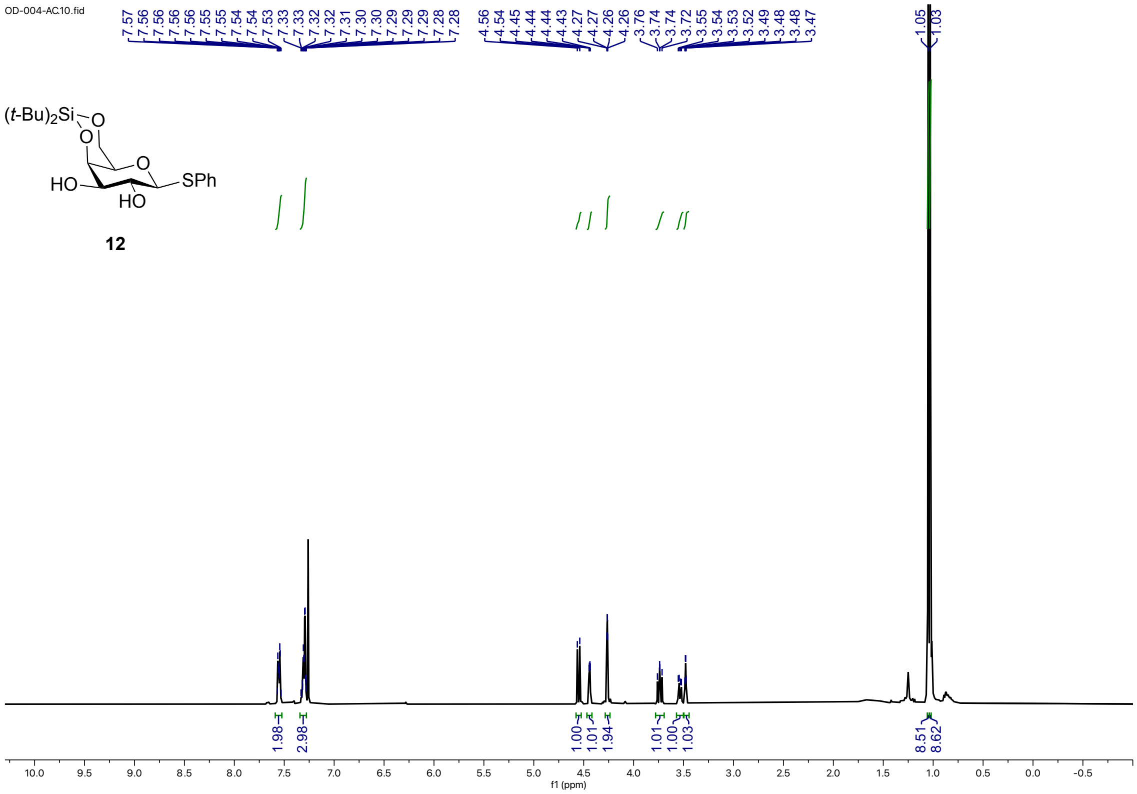

^{13}C NMR ($CDCl_3$, 101 MHz) of compound **12**

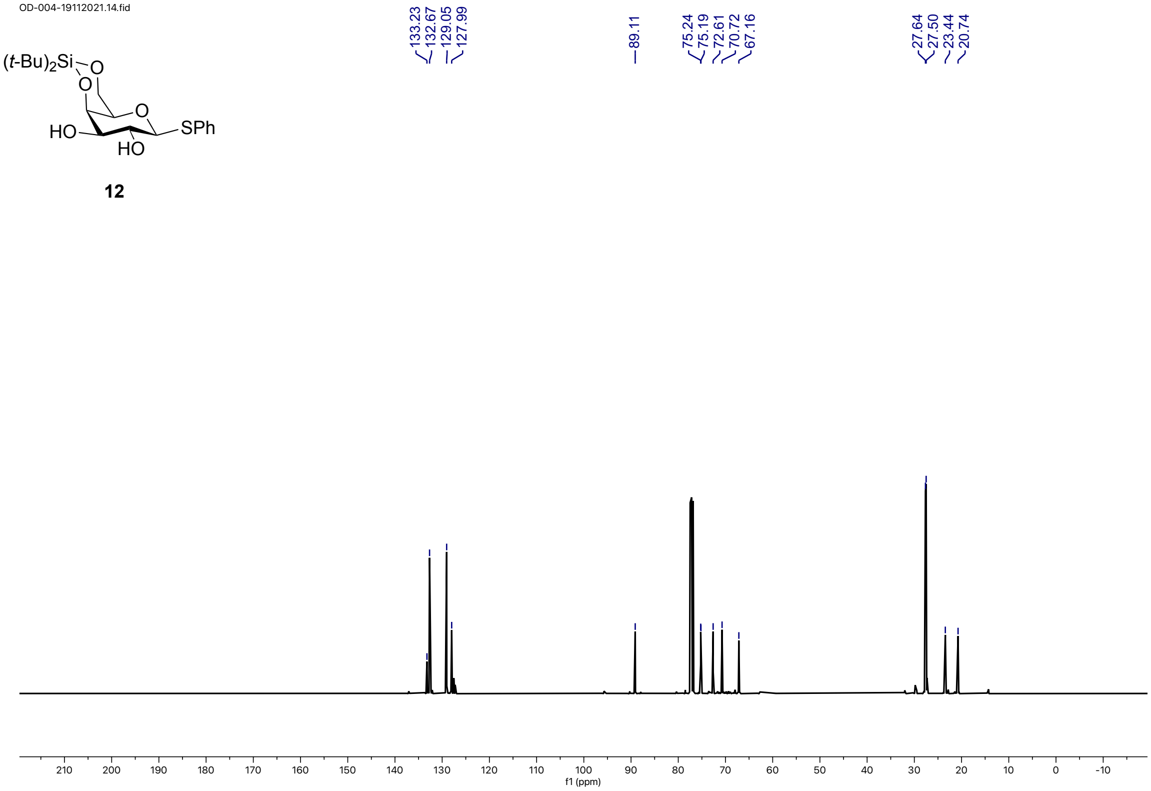

^{1}H-^{1}H COSY ($CDCl_3$, 400 MHz) of compound **12**

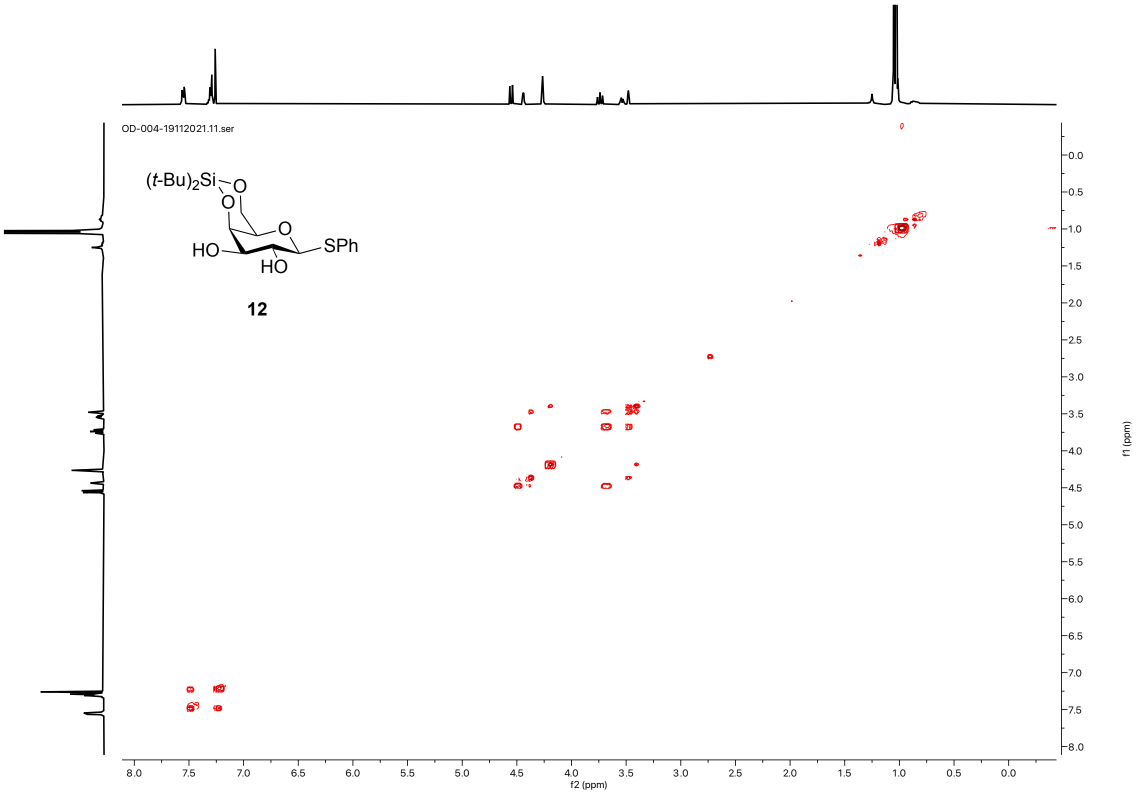

^{1}H-^{13}C HSQC (Decoupling, $CDCl_3$, 400 MHz) of compound **12**

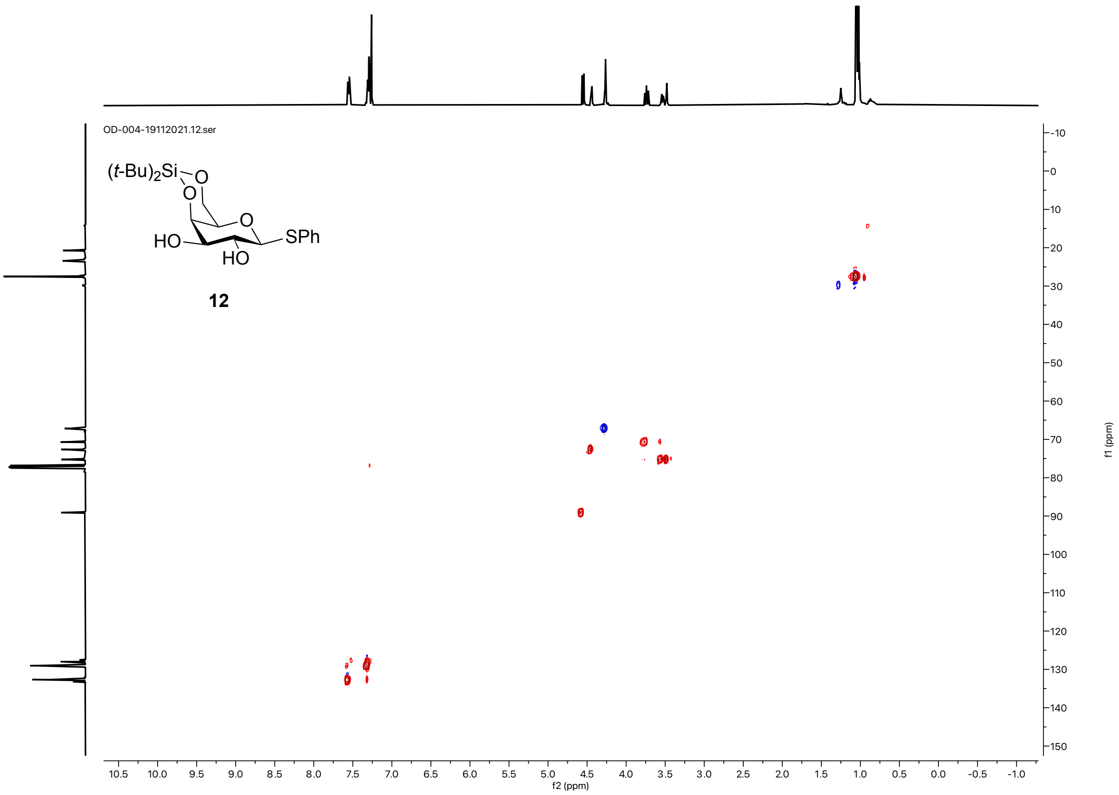

^1H-^{13}C HSQC (No decoupling, $CDCl_3$, 400 MHz) of compound **12**

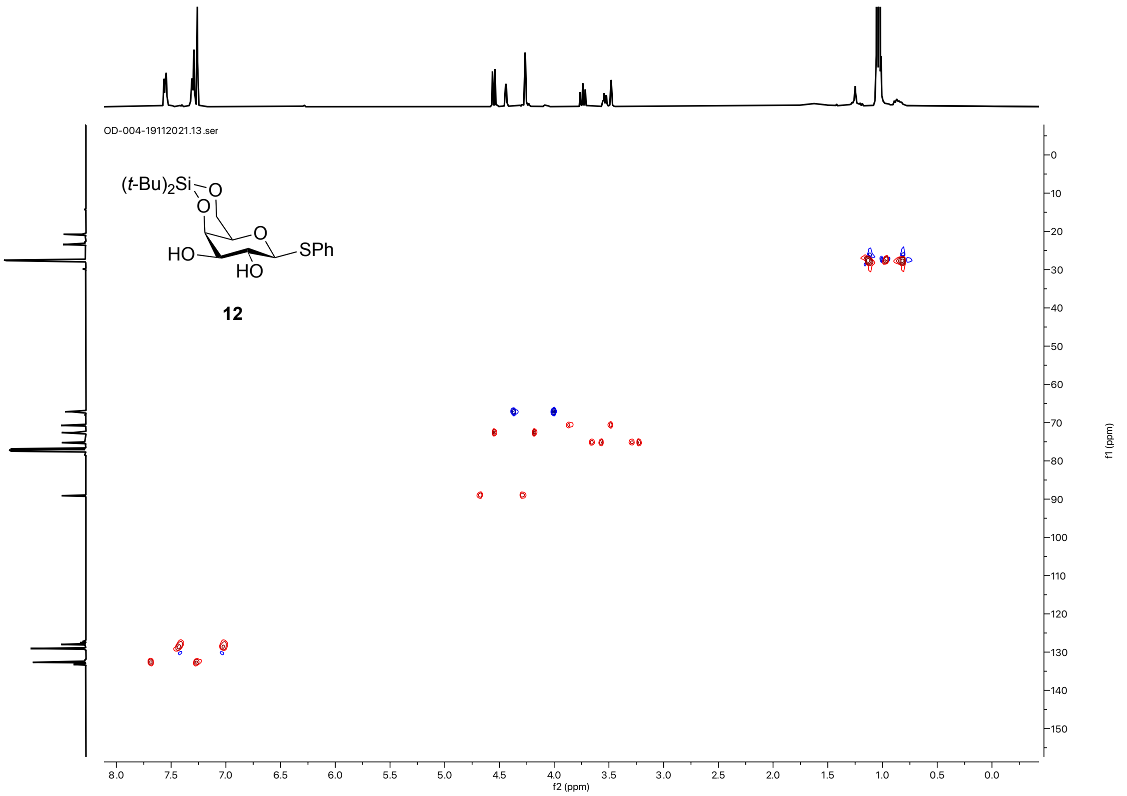

^1H NMR ($CDCl_3$, 400 MHz) of compound **3**

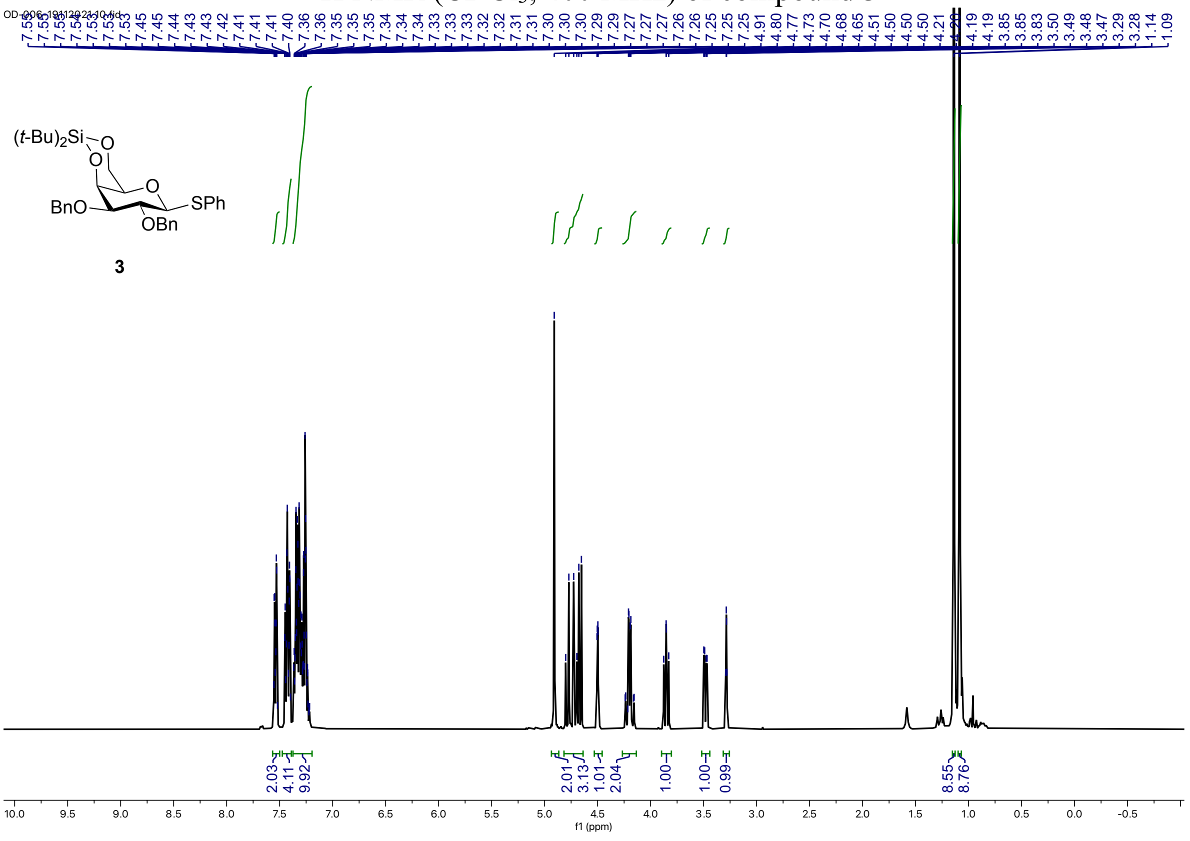

^{13}C NMR ($CDCl_3$, 101 MHz) of compound **3**

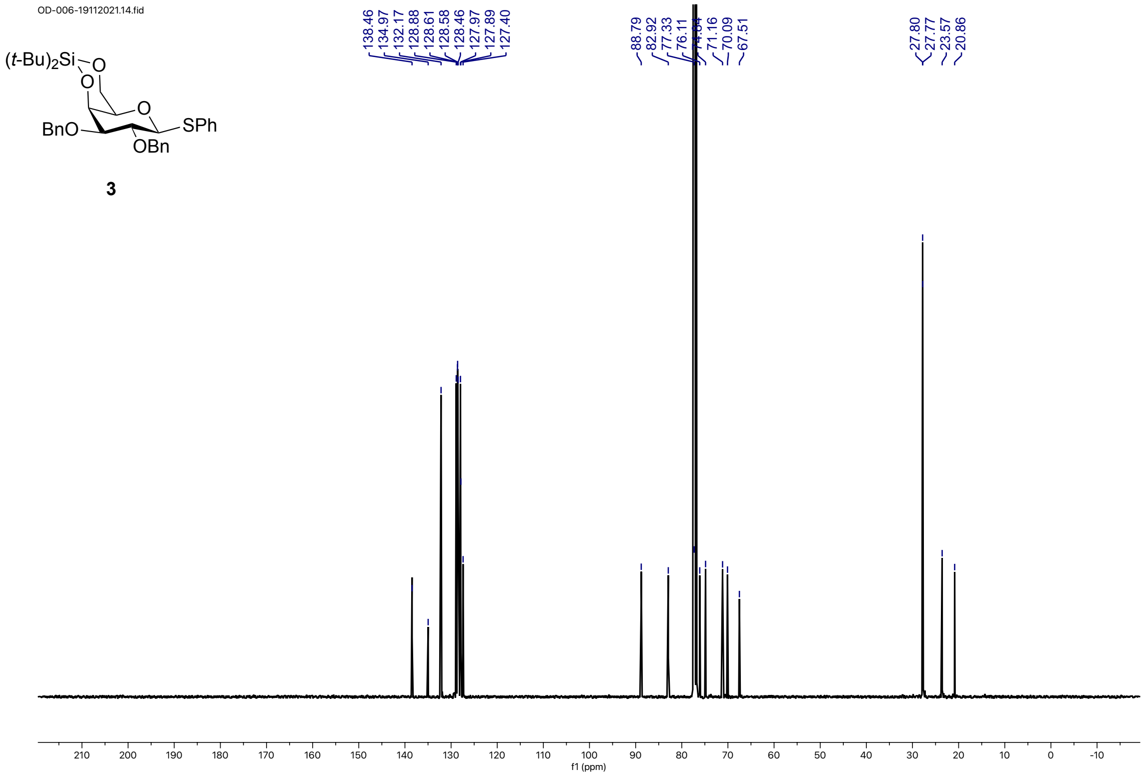

^1H-^1H COSY ($CDCl_3$, 400 MHz) of compound **3**

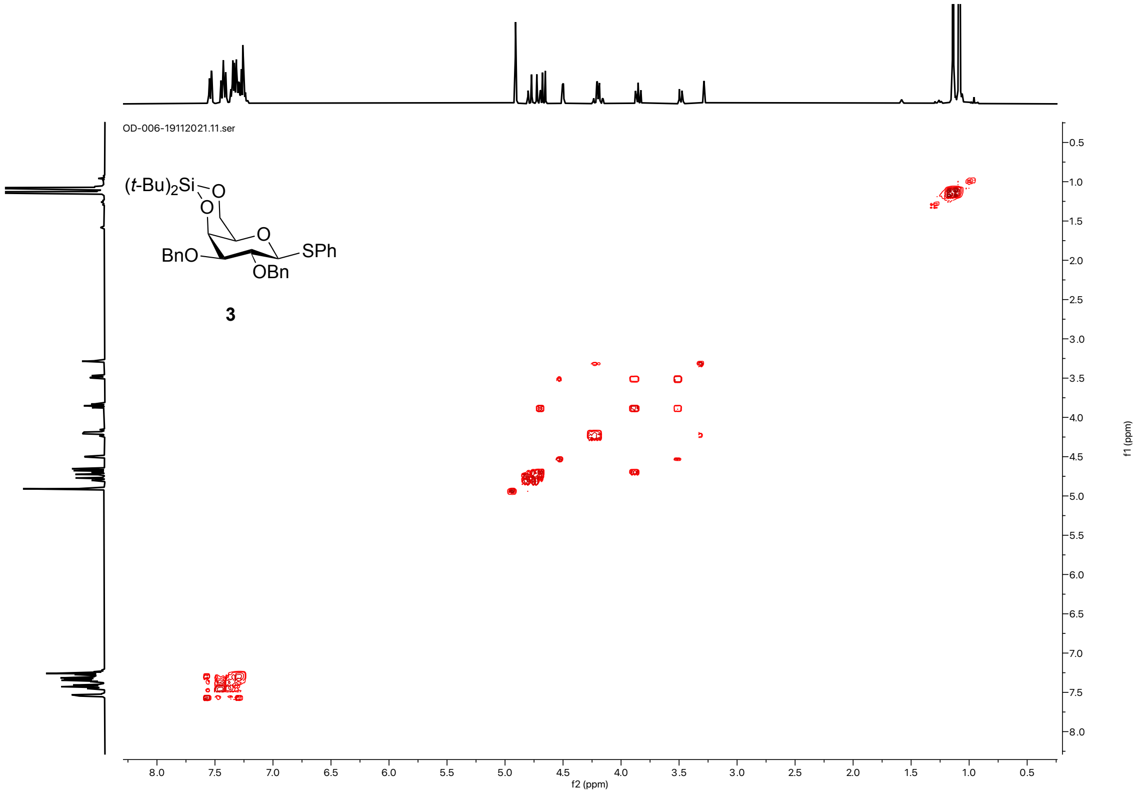

^{1}H-^{13}C HSQC (Decoupling, $CDCl_3$, 400 MHz) of compound **3**

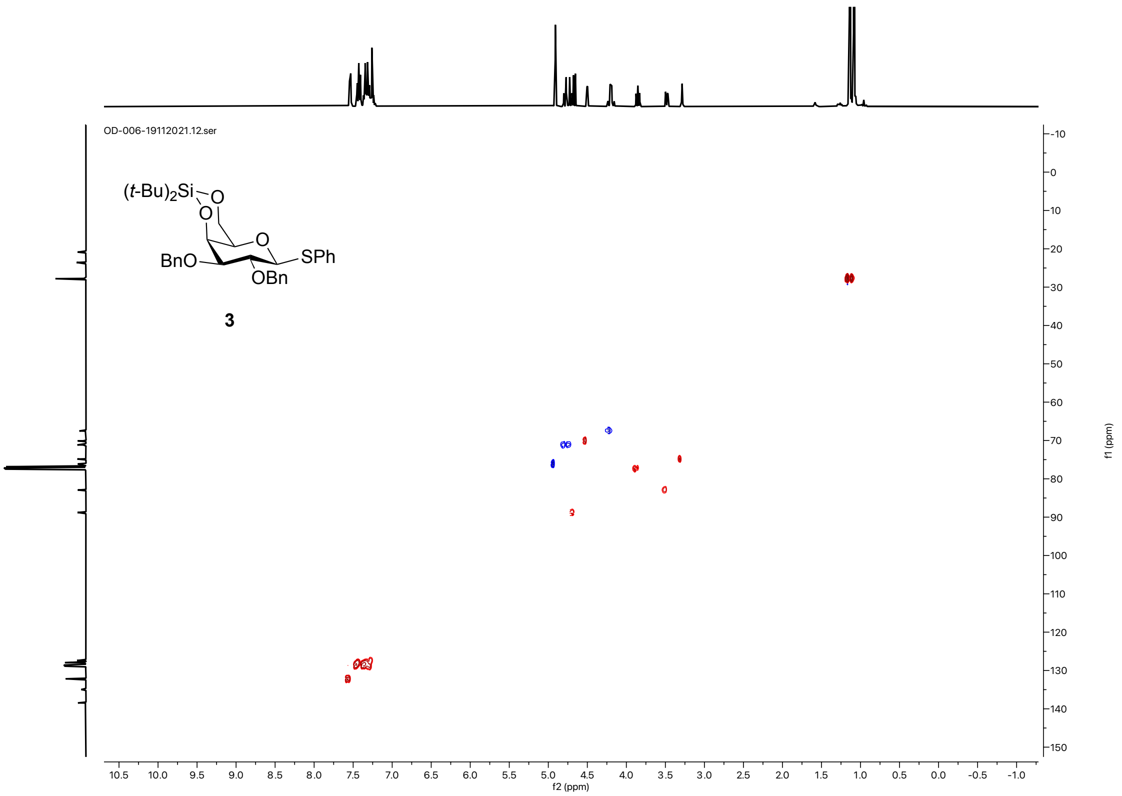

^{1}H-^{13}C HSQC (No decoupling, $CDCl_3$, 400 MHz) of compound **3**

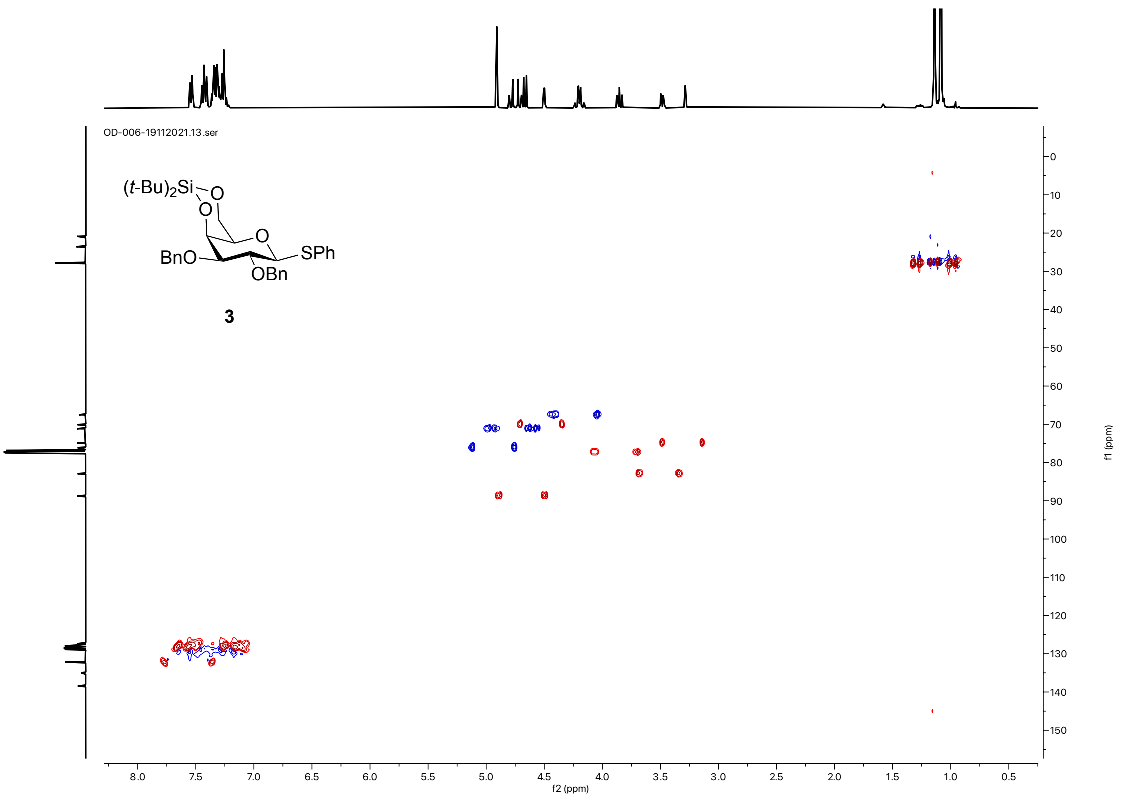

^{1}H NMR ($CDCl_3$, 400 MHz) of compound **13**

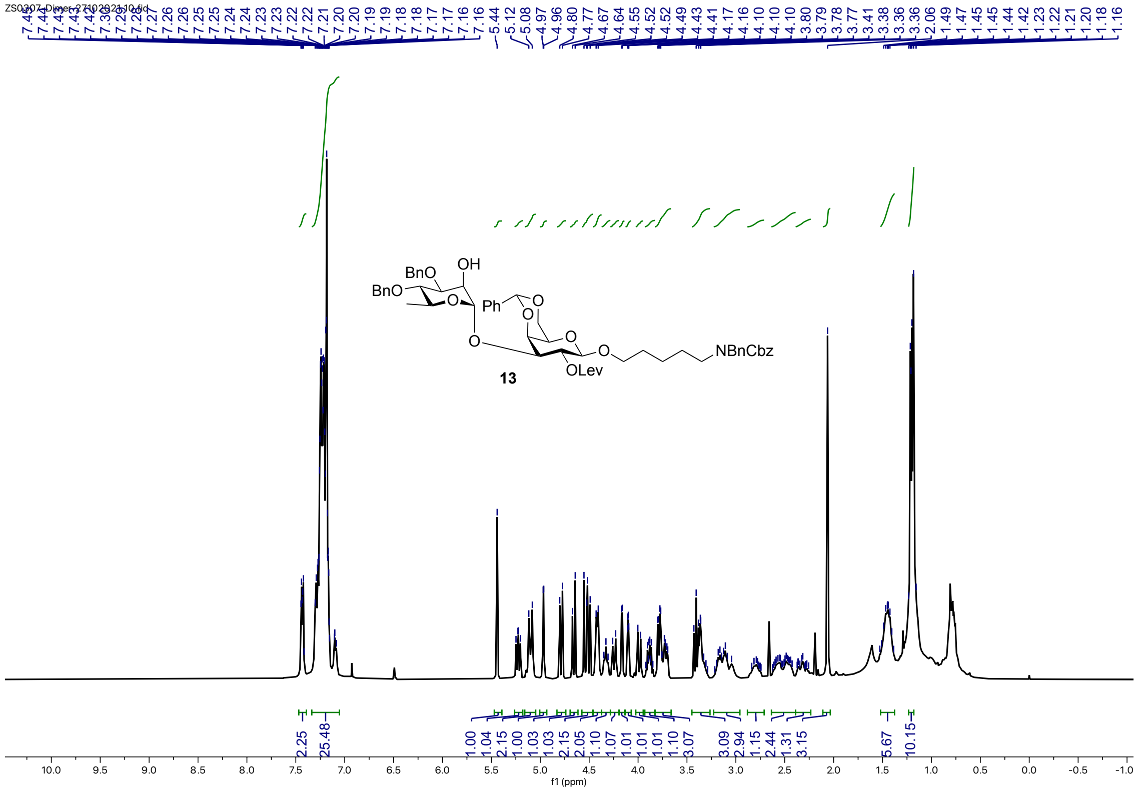

^{13}C NMR ($CDCl_3$, 101 MHz) of compound **13**

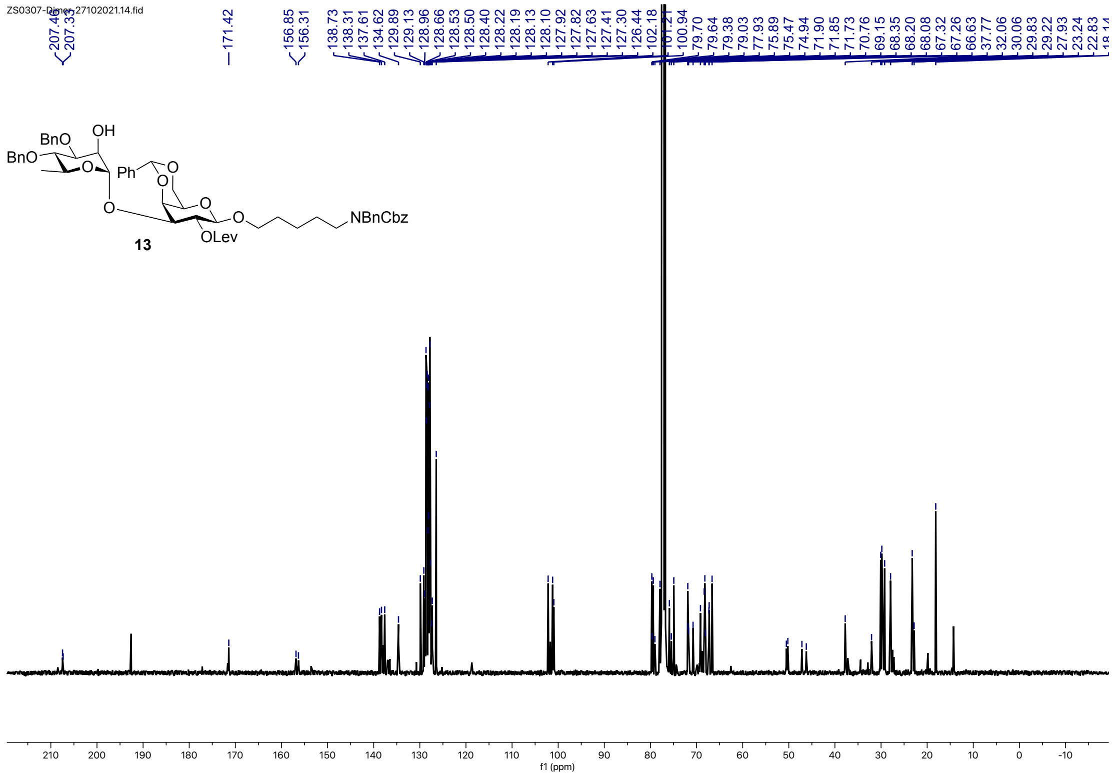

^{1}H-^{1}H COSY ($CDCl_3$, 400 MHz) of compound **13**

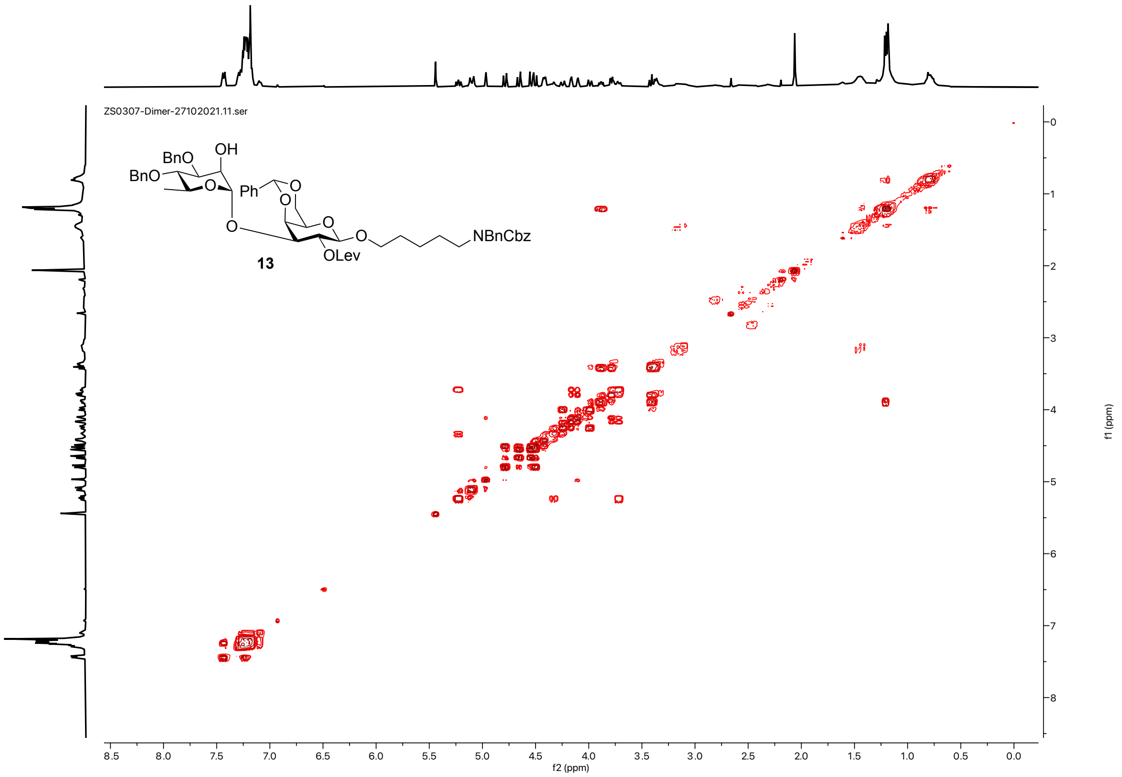

^{1}H-^{13}C HSQC (Decoupling, $CDCl_3$, 400 MHz) of compound **13**

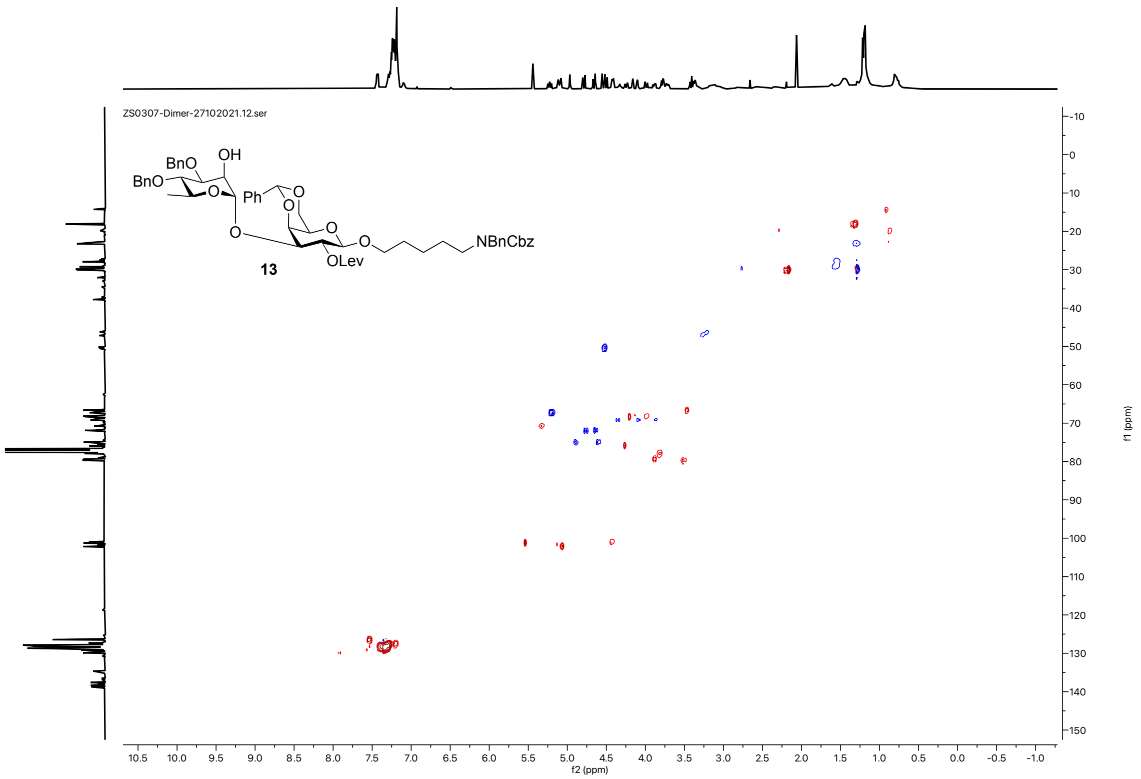

^{1}H-^{13}C HSQC (No decoupling, $CDCl_3$, 400 MHz) of compound **13**

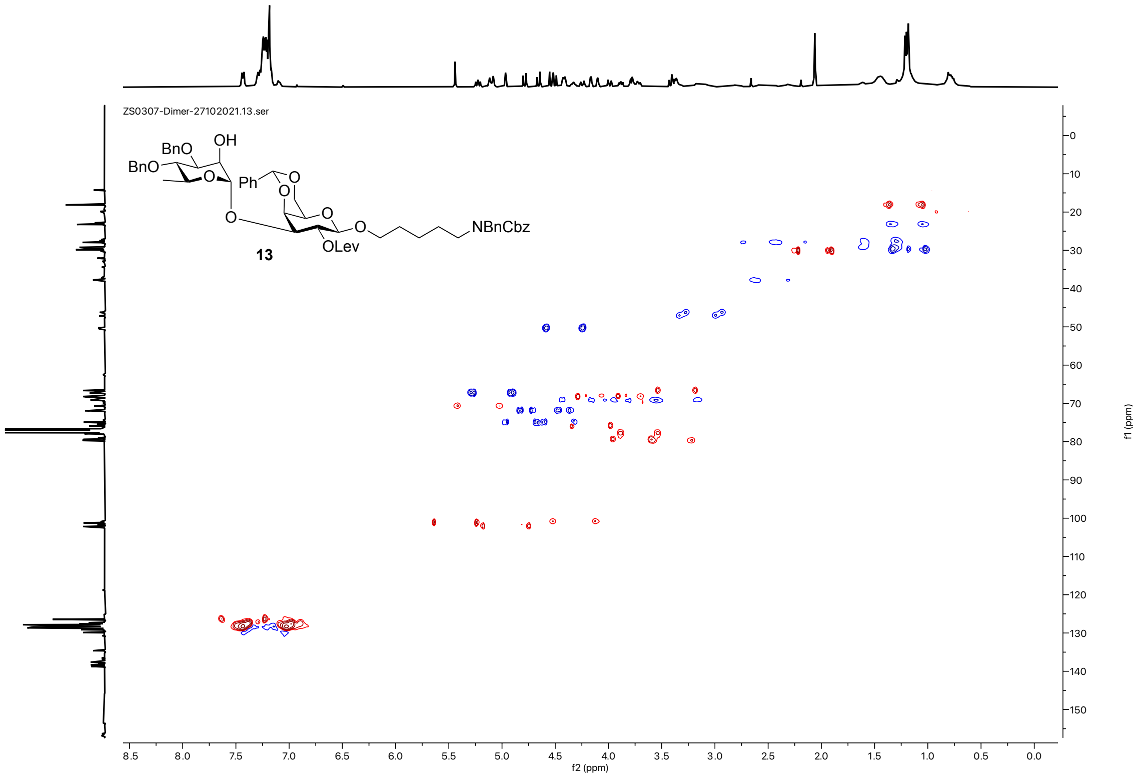

^{1}H NMR ($CDCl_3$, 600 MHz) of compound **14**

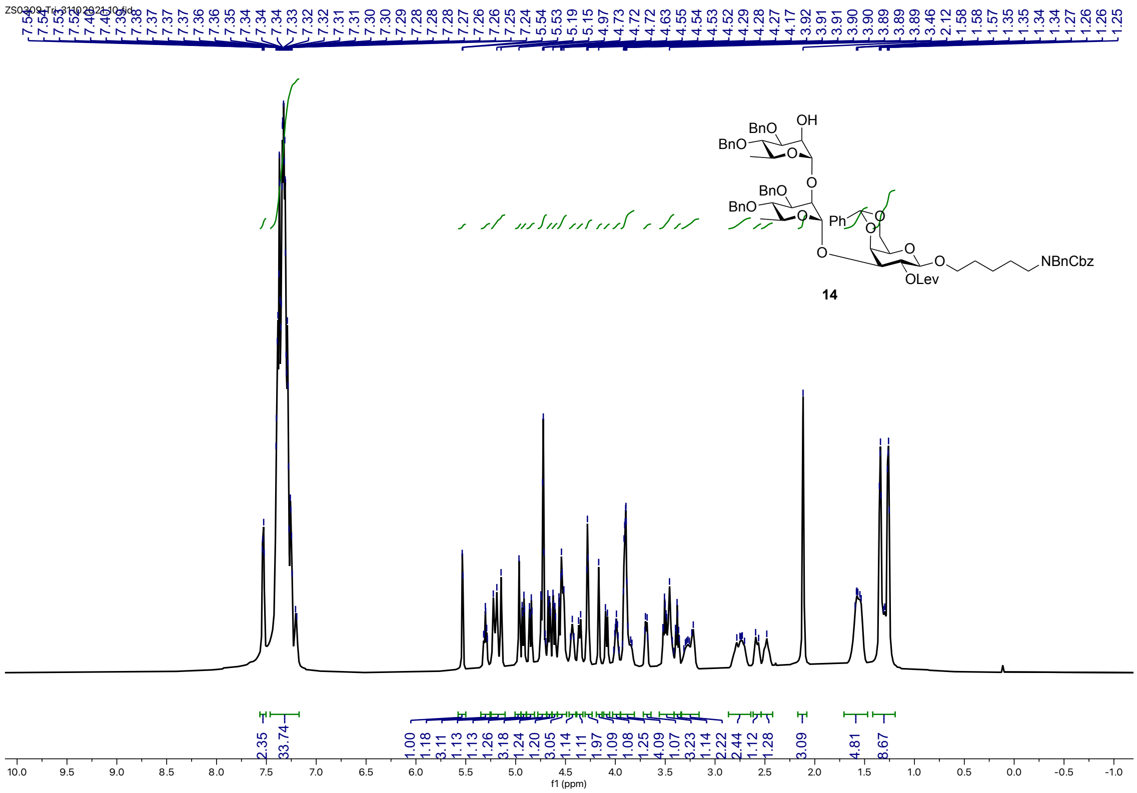

^{13}C NMR ($CDCl_3$, 151 MHz) of compound **14**

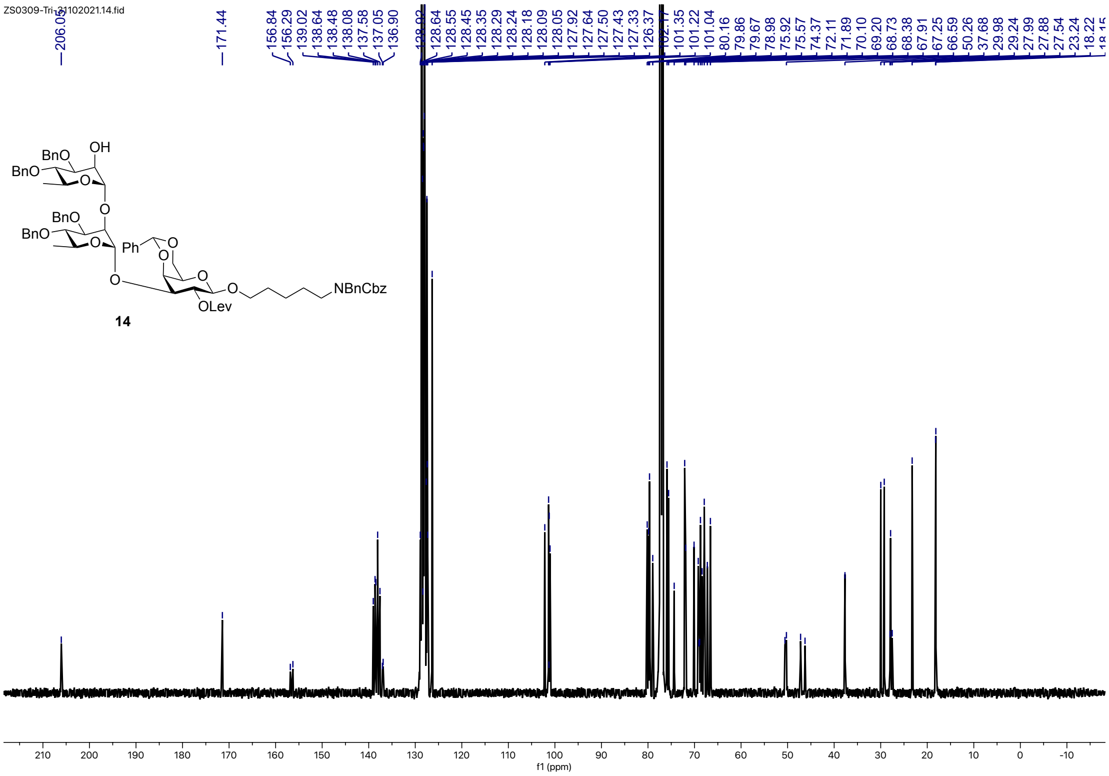

^{1}H-^{1}H COSY ($CDCl_3$, 600 MHz) of compound **14**

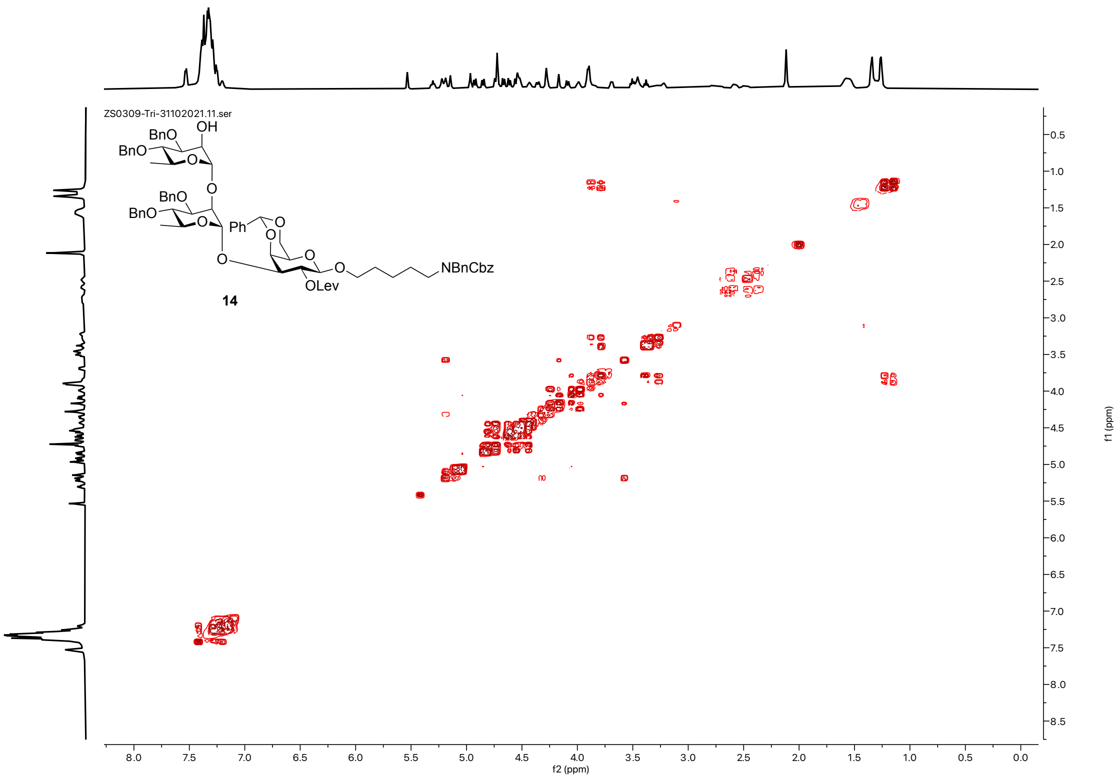

^{1}H-^{13}C HSQC (Decoupling, $CDCl_3$, 600 MHz) of compound **14**

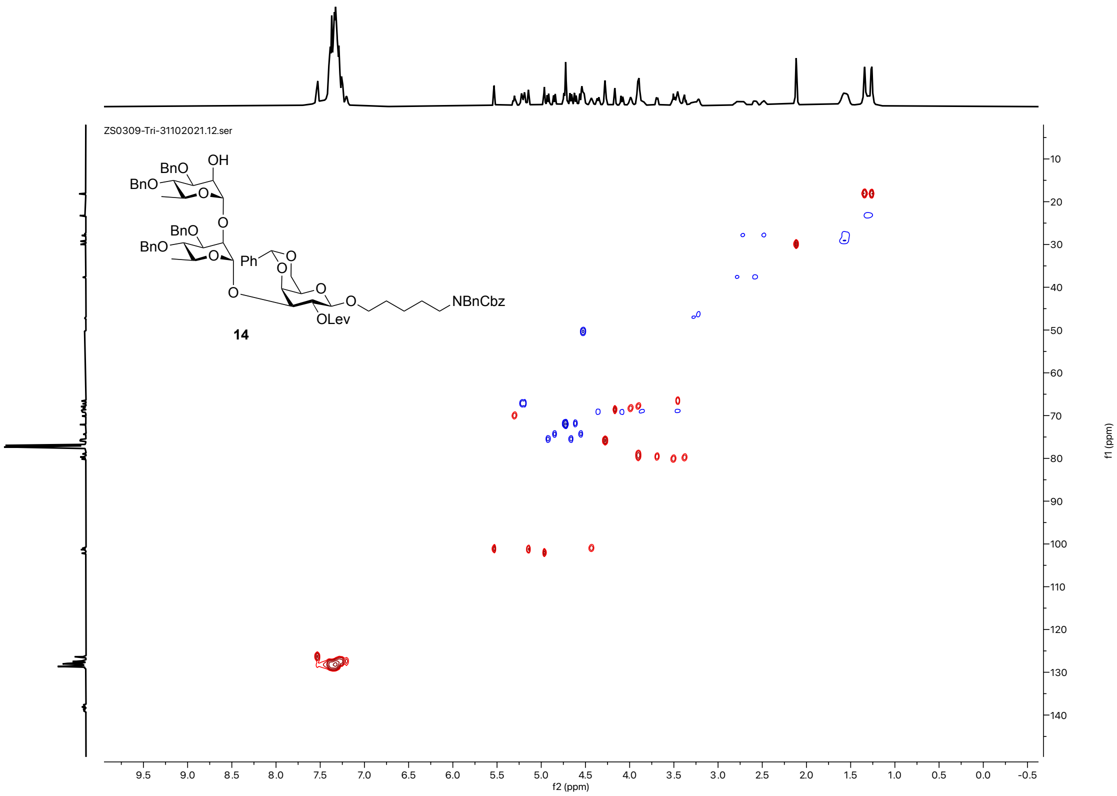

^{1}H-^{13}C HSQC (No decoupling, $CDCl_3$, 600 MHz) of compound **14**

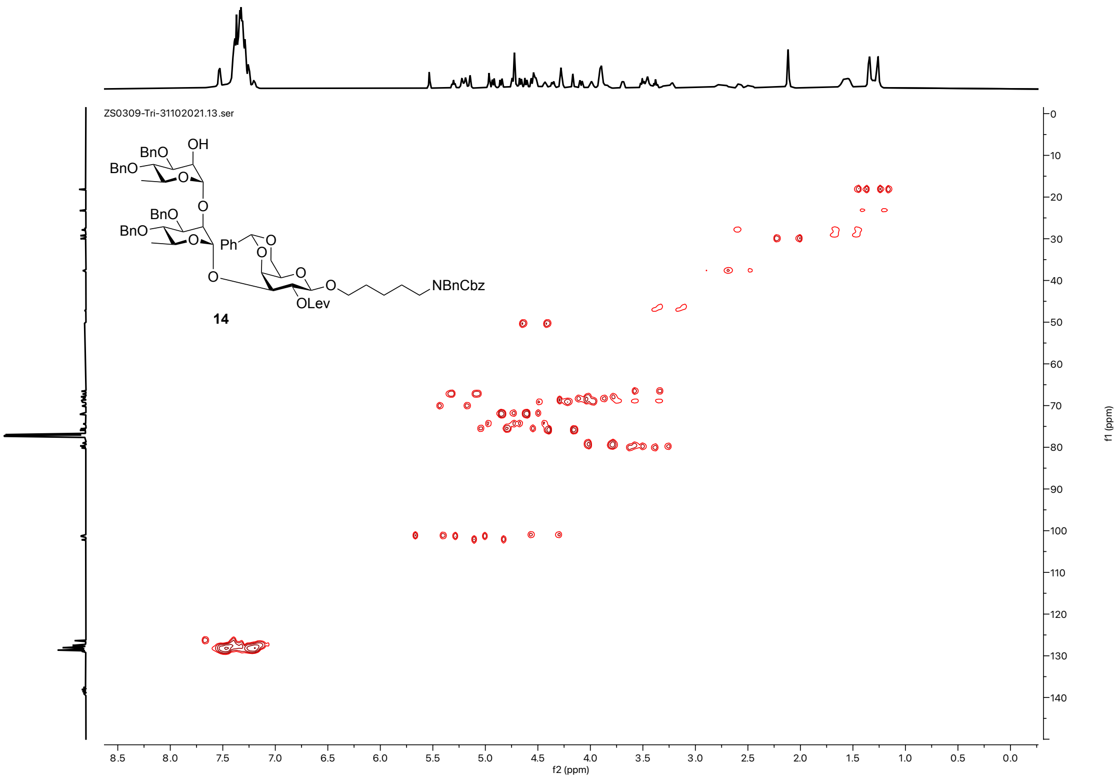

^{1}H NMR ($CDCl_3$, 600 MHz) of compound **15**

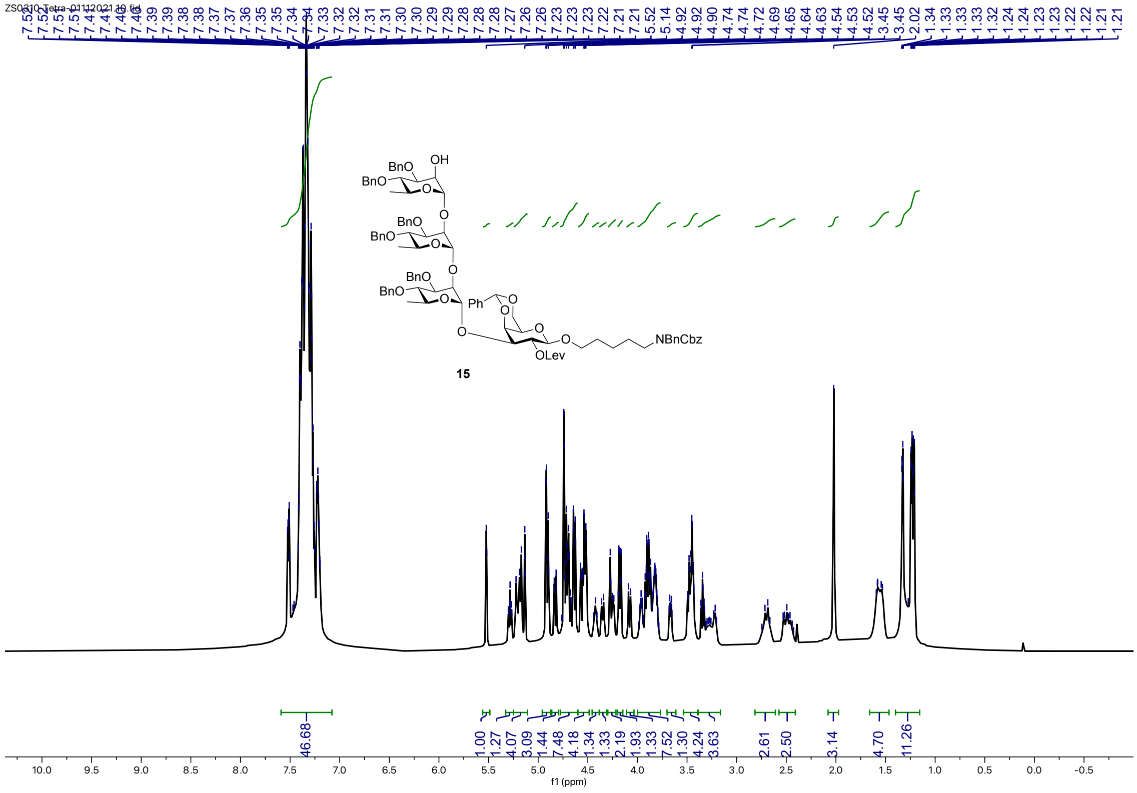

^{13}C NMR ($CDCl_3$, 151 MHz) of compound **15**

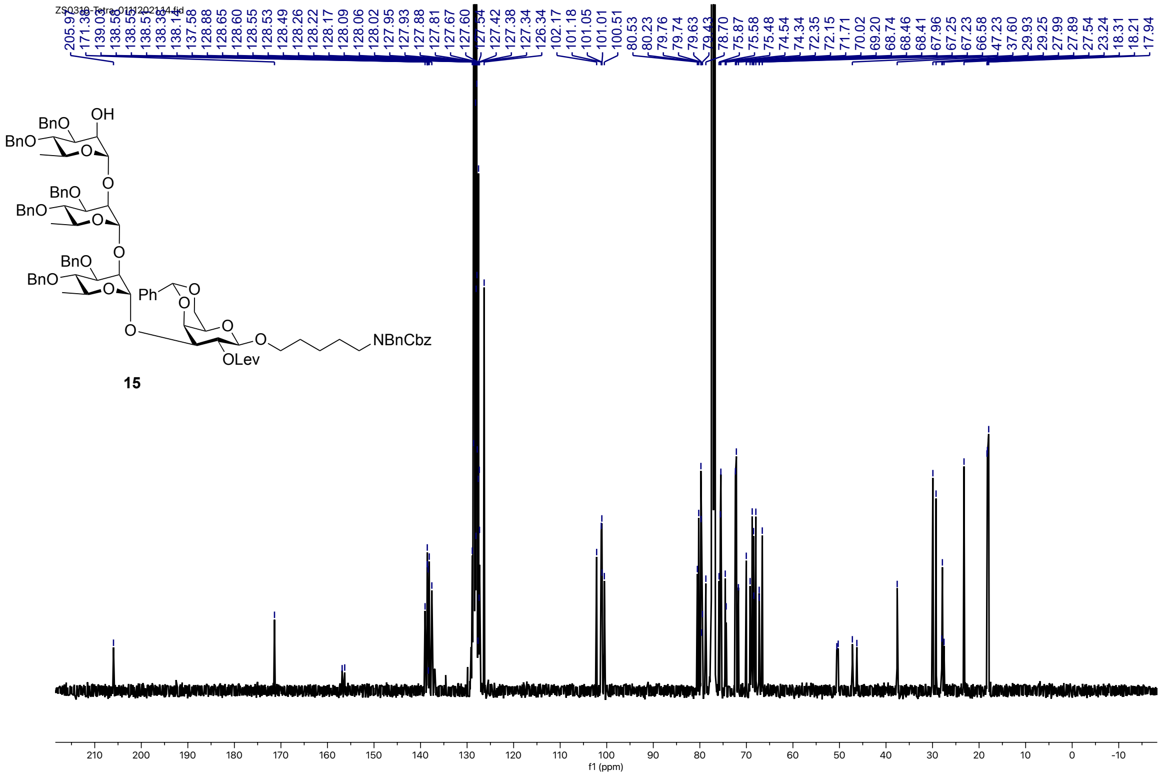

^{1}H-^{1}H COSY ($CDCl_3$, 600 MHz) of compound **15**

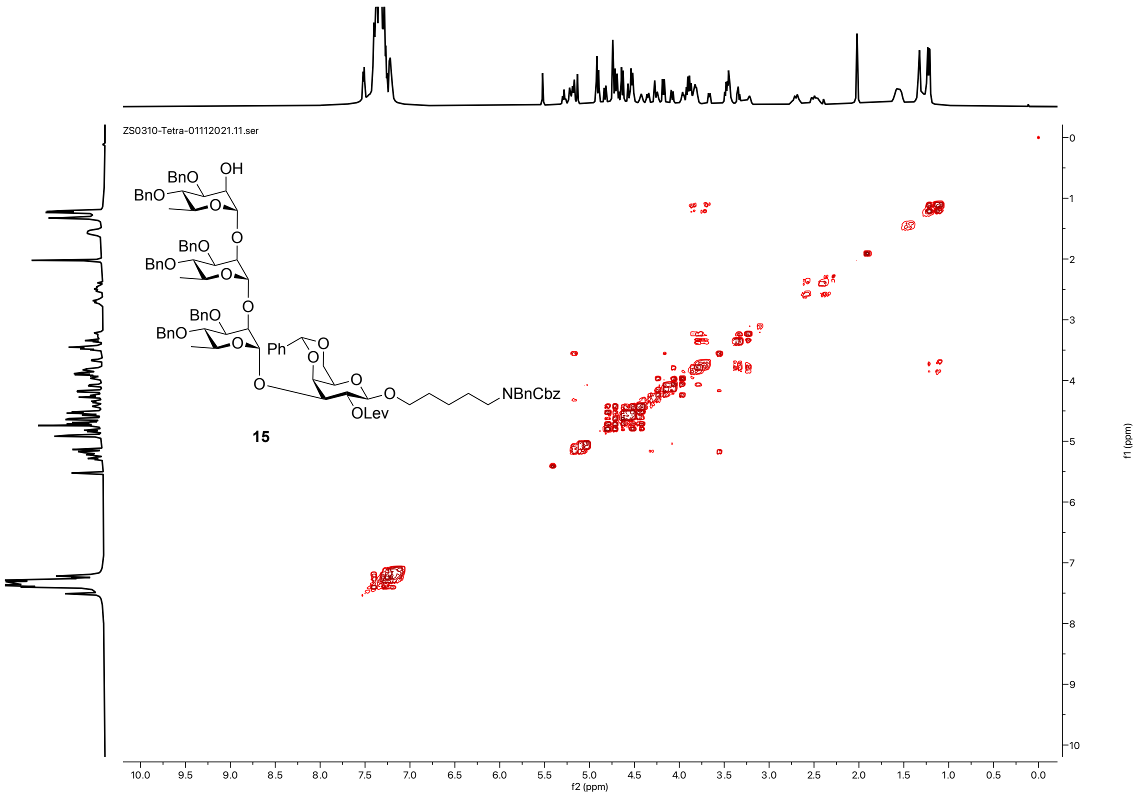

^{1}H-^{13}C HSQC (Decoupling, $CDCl_3$, 600 MHz) of compound **15**

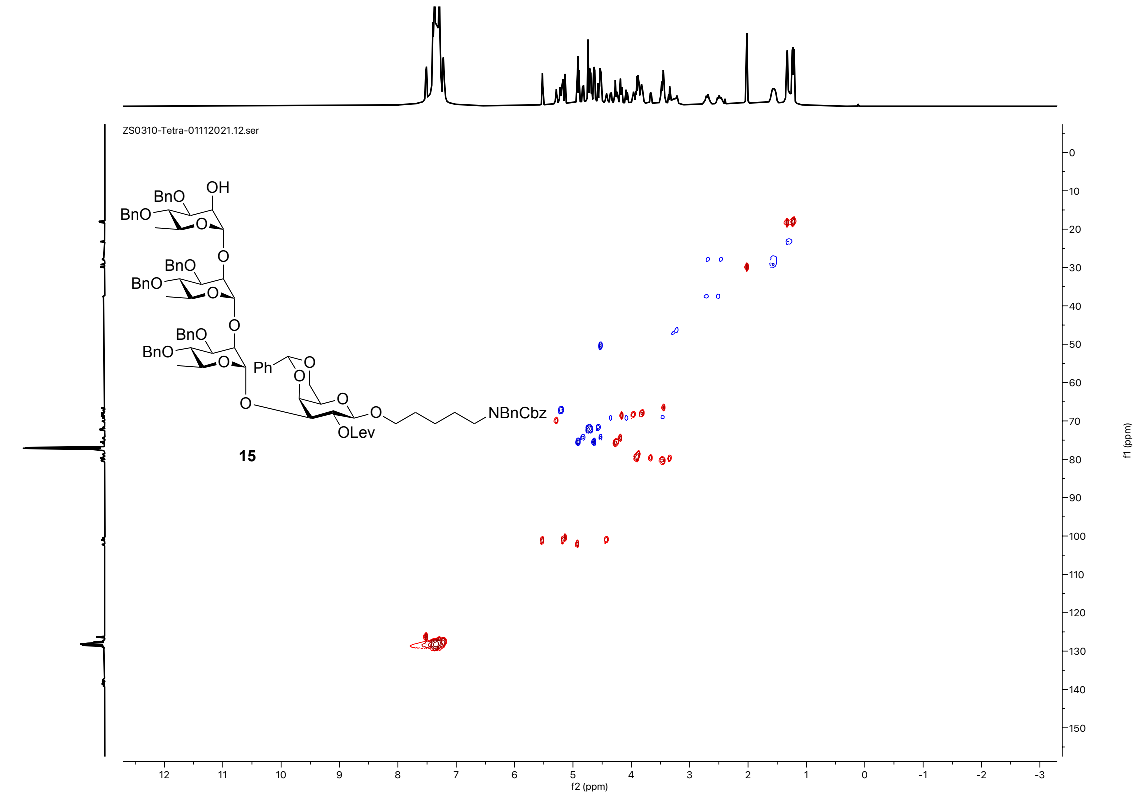

^{1}H-^{13}C HSQC (No decoupling, $CDCl_3$, 600 MHz) of compound **15**

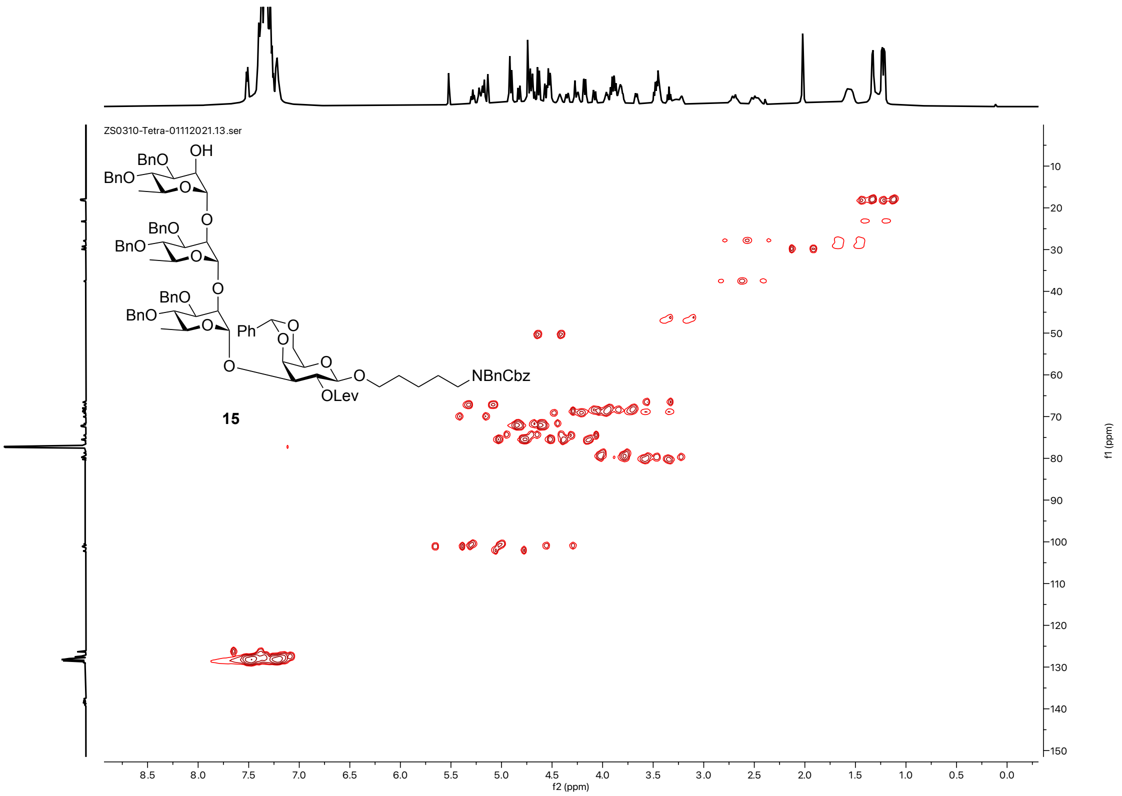

^{1}H NMR ($CDCl_3$, 600 MHz) of compound **16**

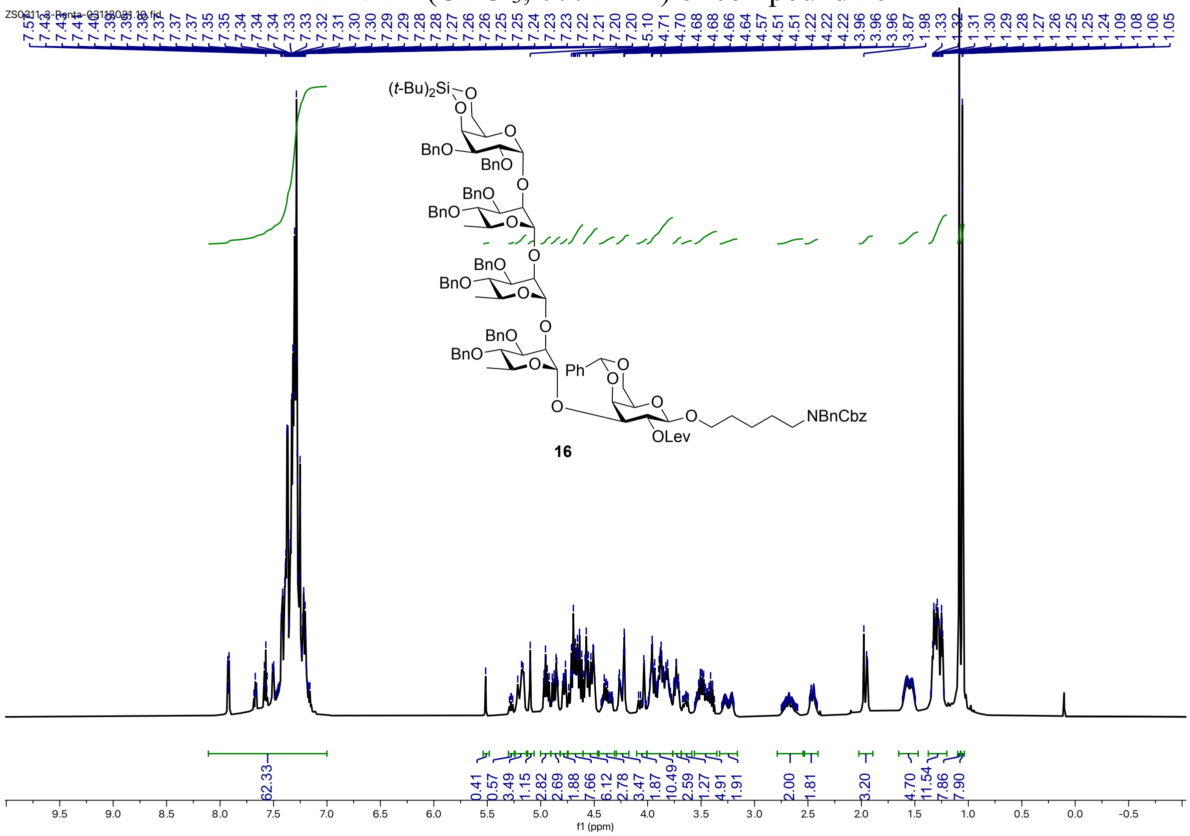

^{13}C NMR (CDCl$_3$, 151 MHz) of compound **16**

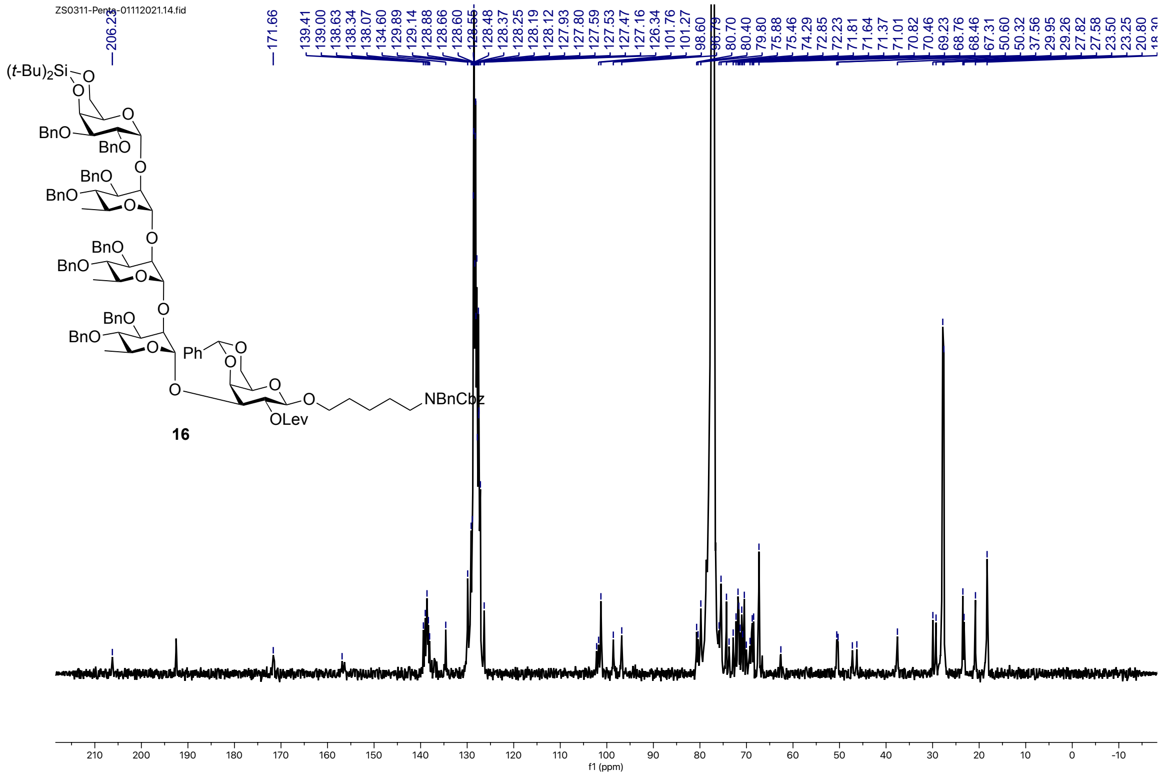

^{1}H-^{1}H COSY (CDCl$_3$, 600 MHz) of compound **16**

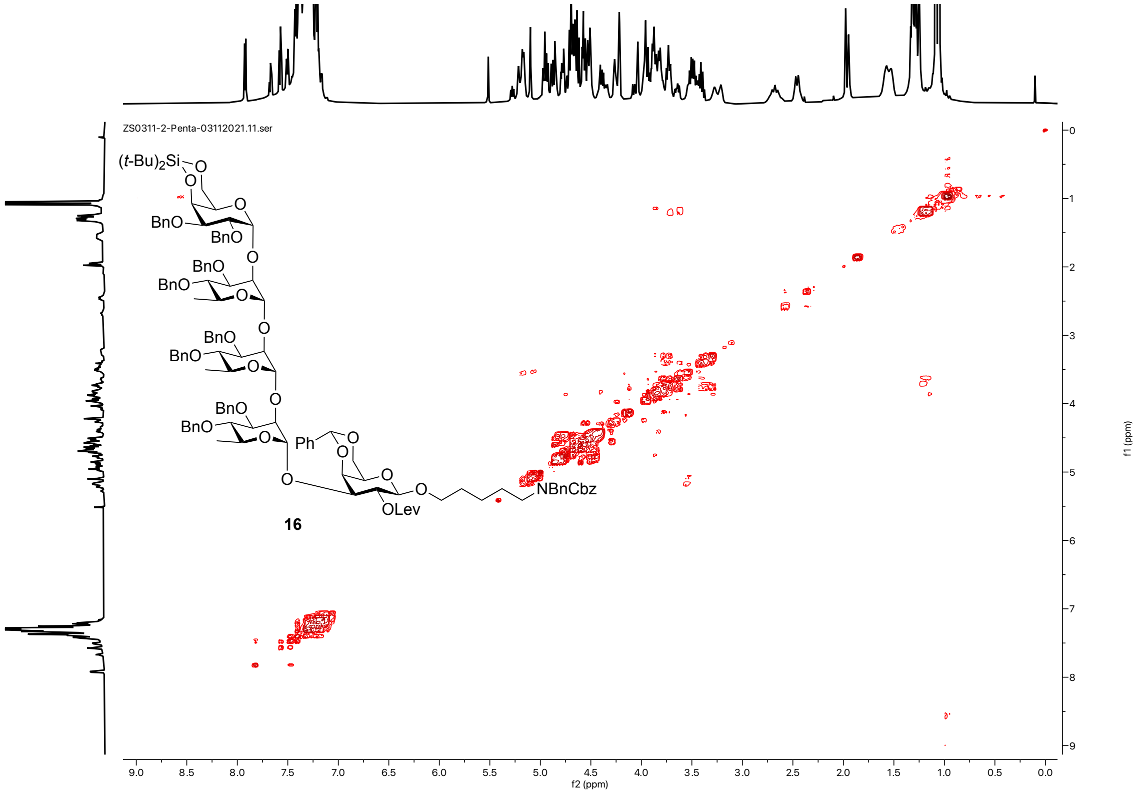

^{1}H-^{13}C HSQC (Decoupling, $CDCl_3$, 600 MHz) of compound **16**

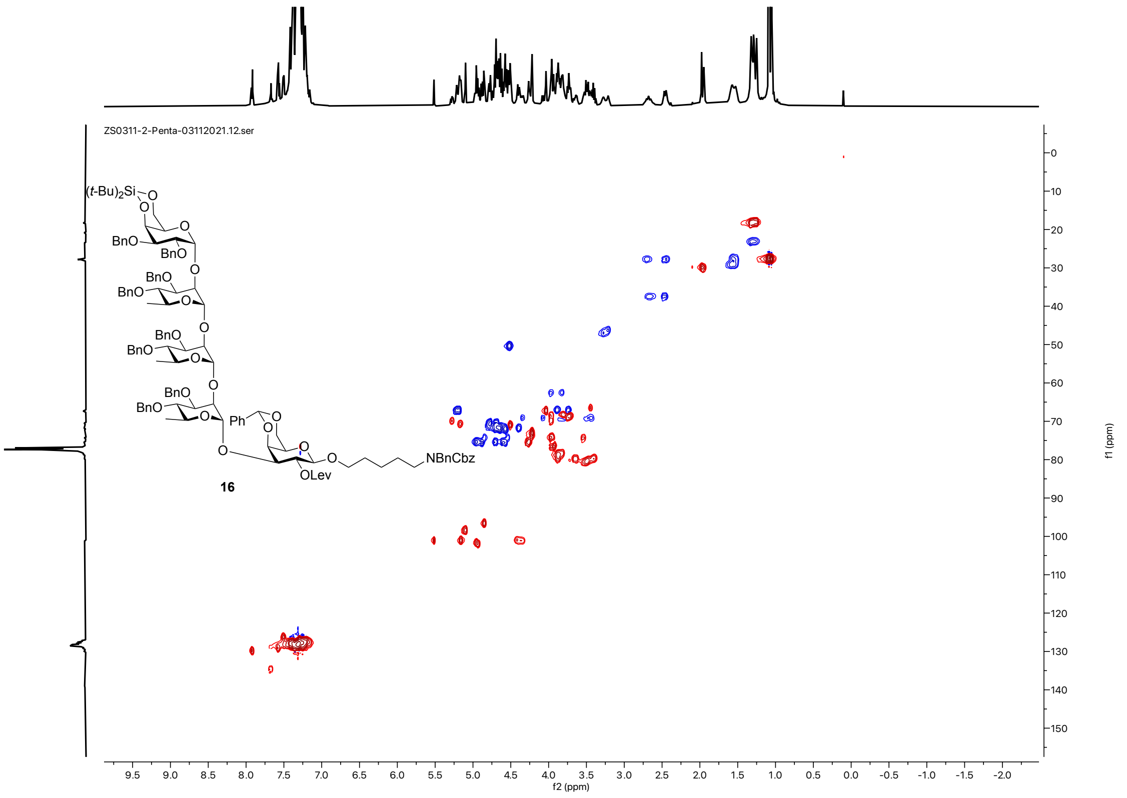

^{1}H-^{13}C HSQC (No decoupling, $CDCl_3$, 600 MHz) of compound **16**

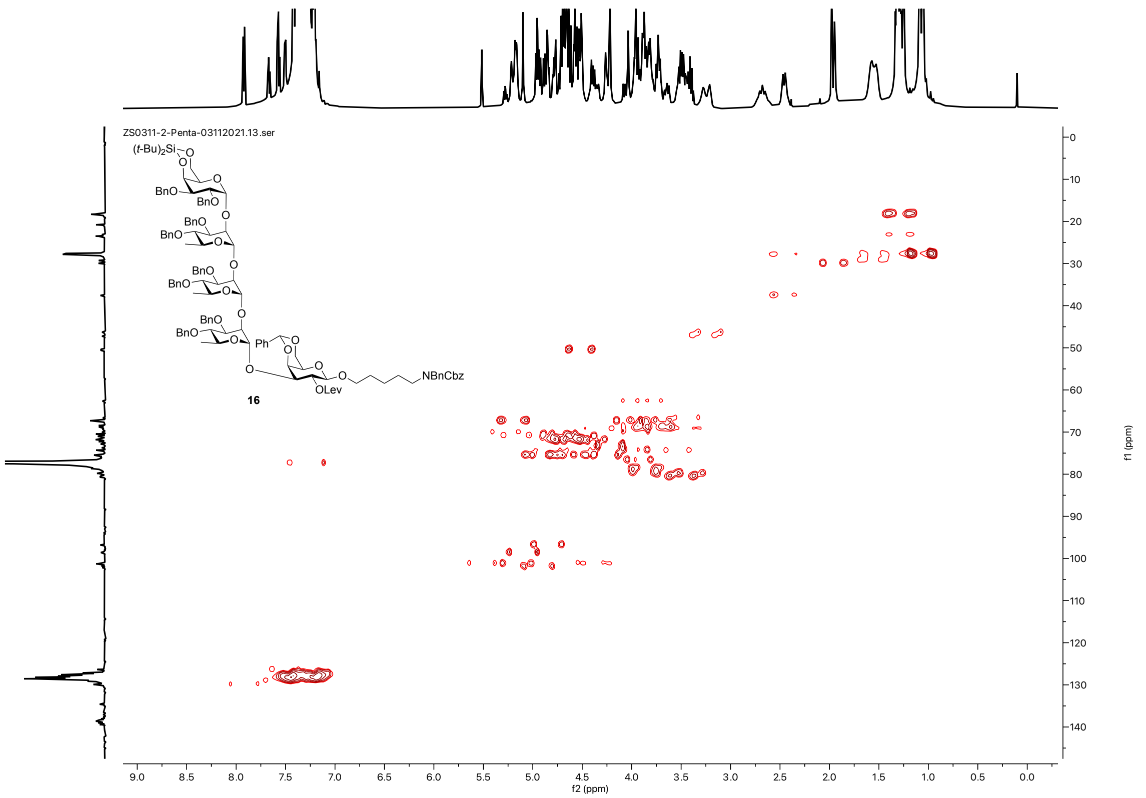

^{1}H NMR ($CDCl_3$, 600 MHz) of compound **17**

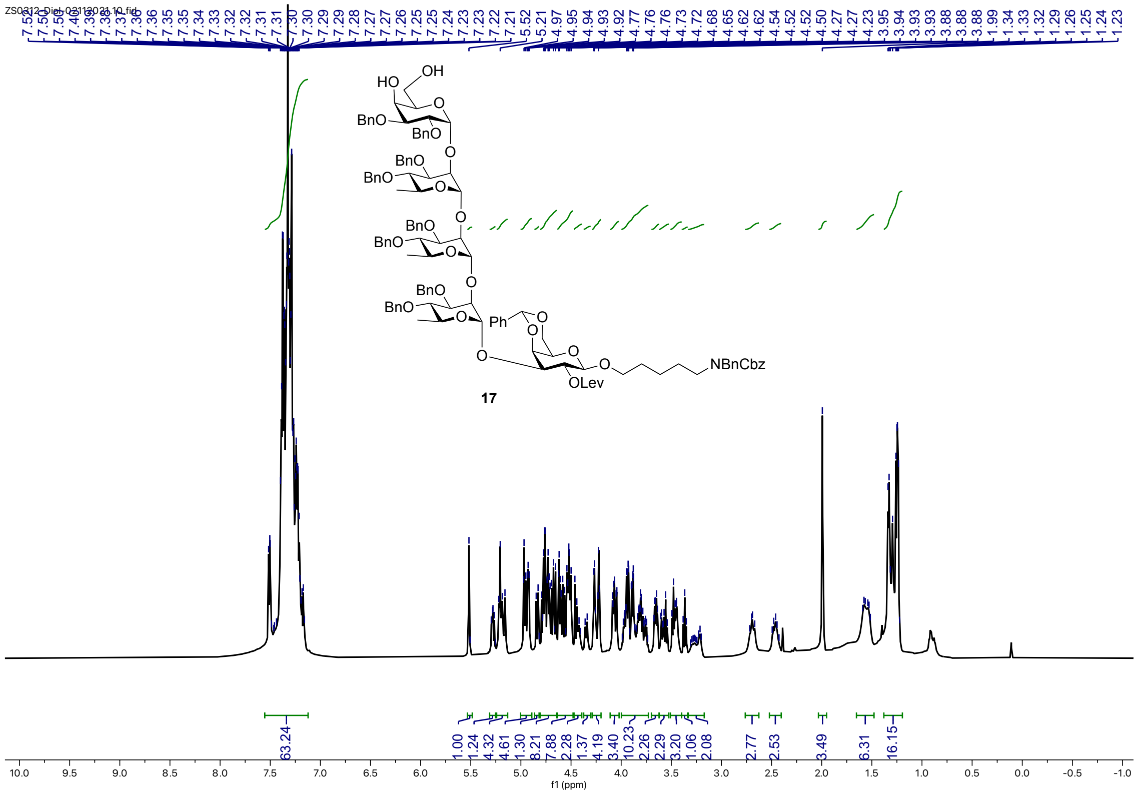

^{13}C NMR ($CDCl_3$, 151 MHz) of compound **17**

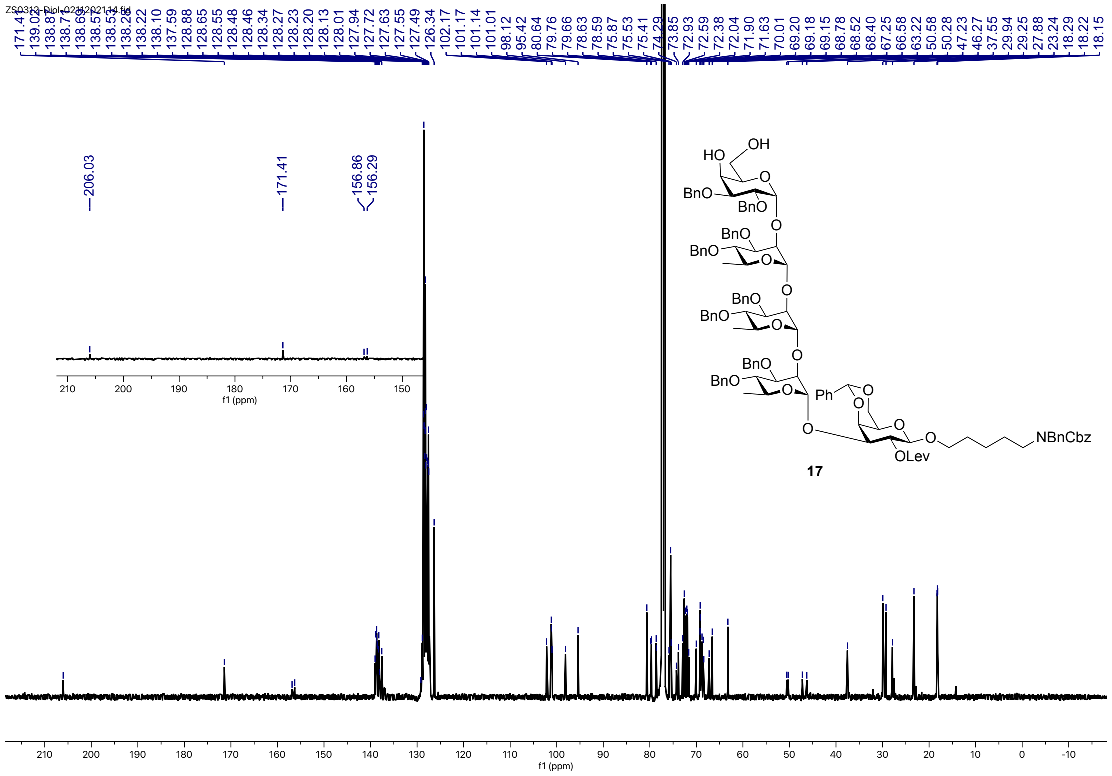

^{1}H-^{1}H COSY ($CDCl_3$, 600 MHz) of compound **17**

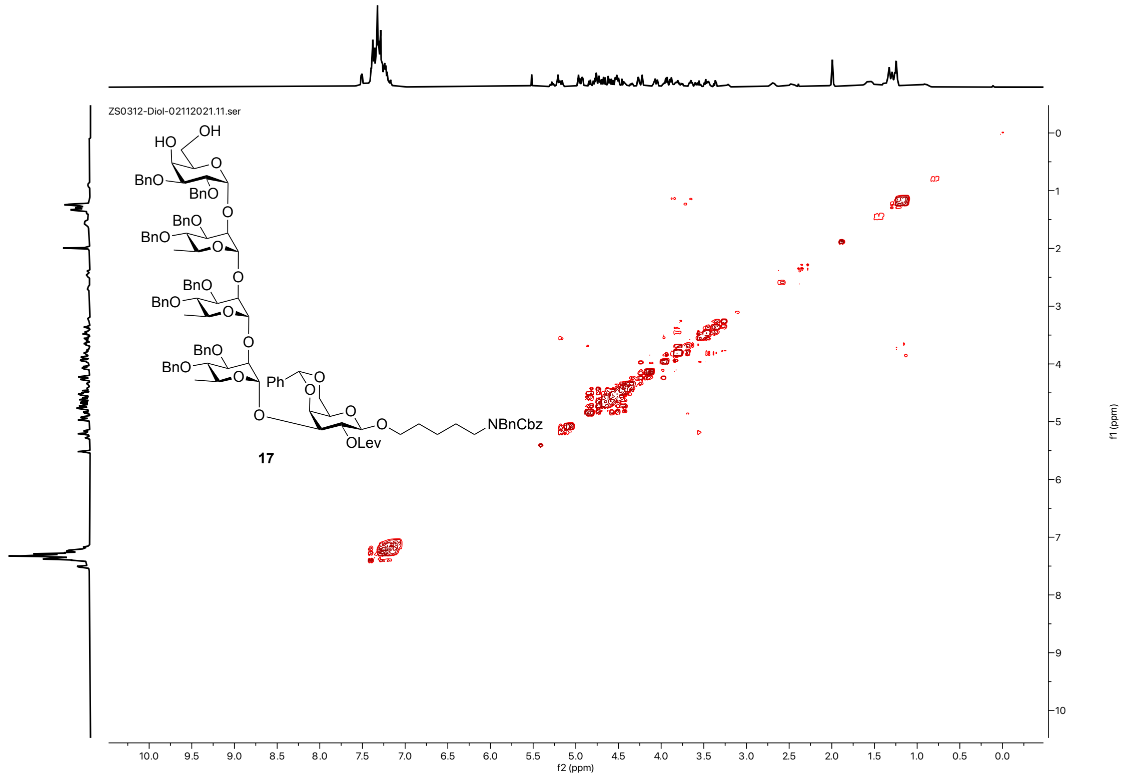

^{1}H-^{13}C HSQC (Decoupling, $CDCl_3$, 600 MHz) of compound **17**

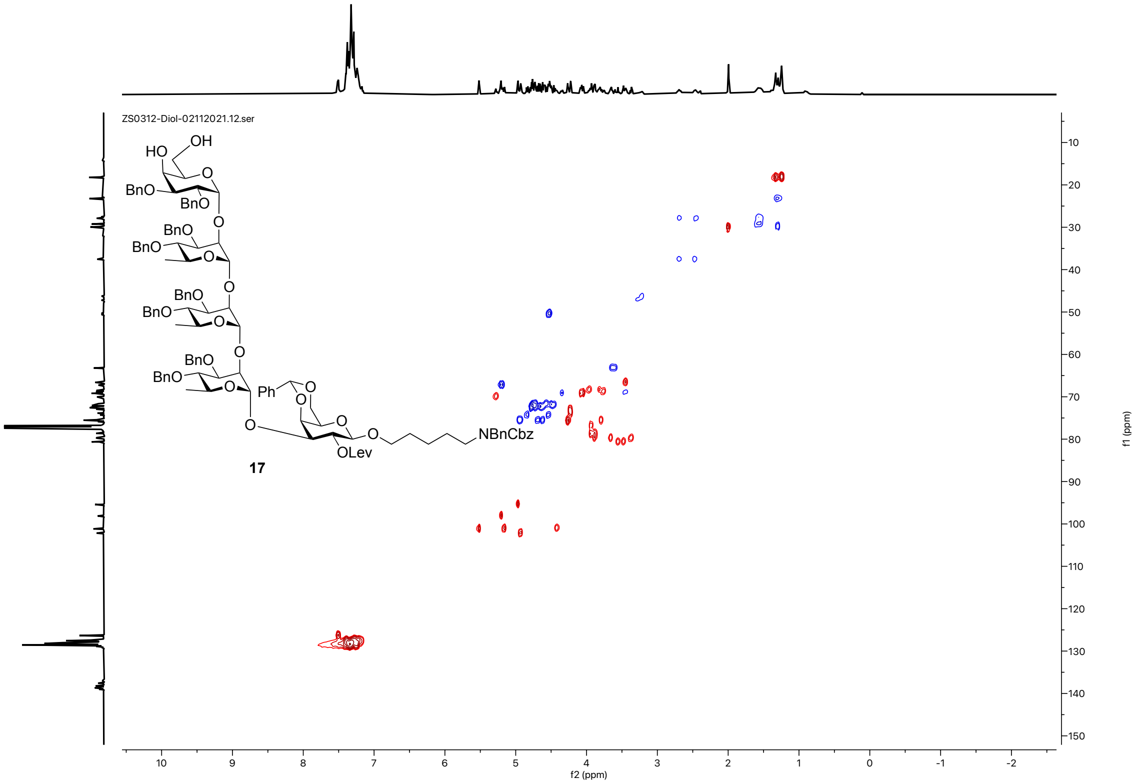

^{1}H-^{13}C HSQC (No decoupling, $CDCl_3$, 600 MHz) of compound **17**

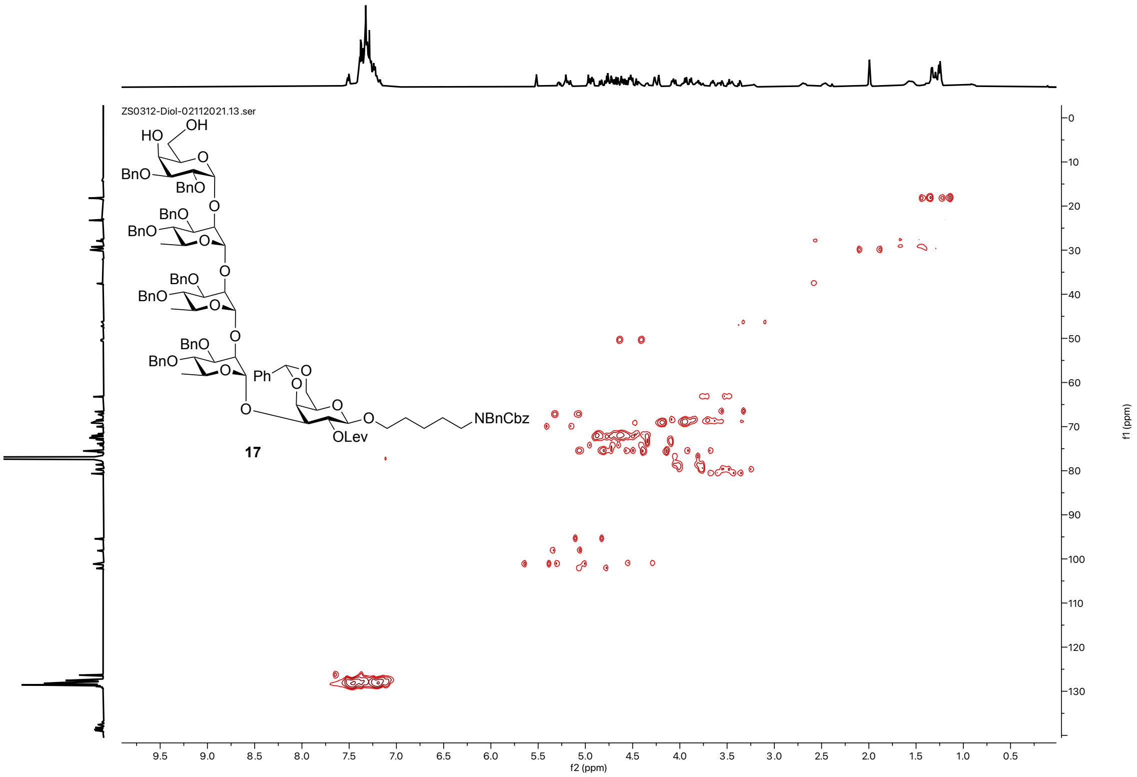

^{1}H NMR ($CDCl_3$, 400 MHz) of compound **18**

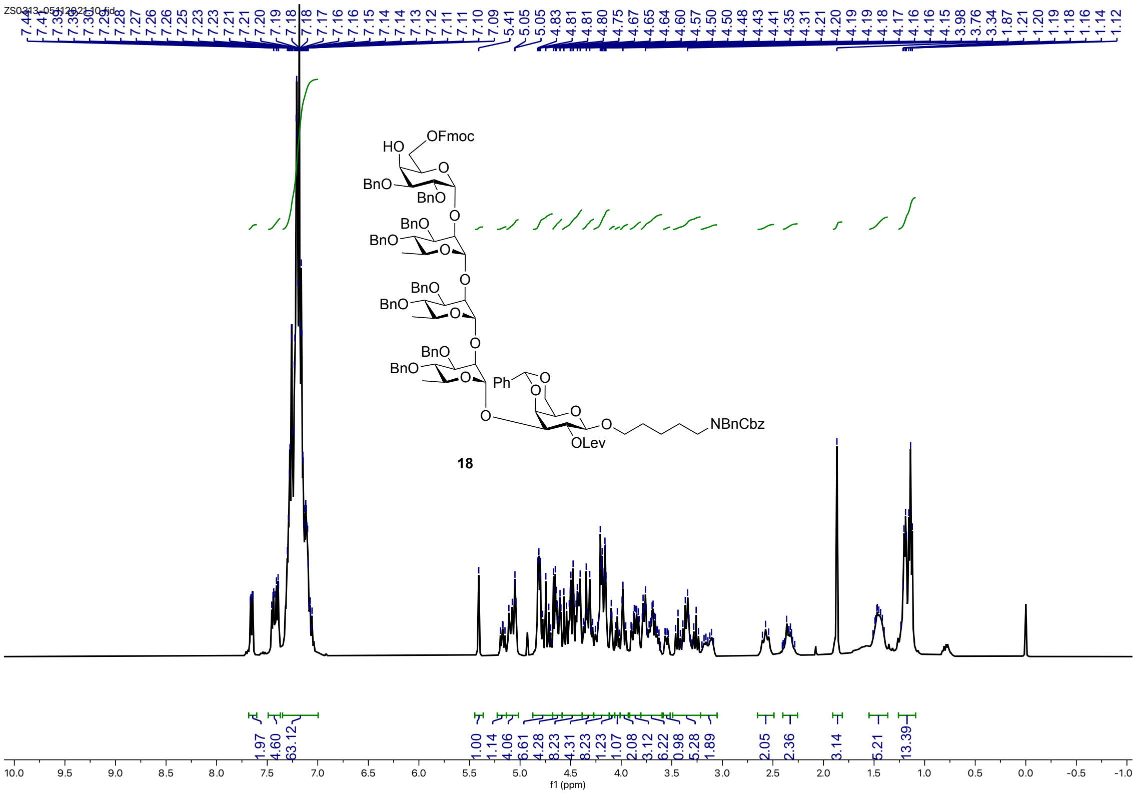

^{13}C NMR (CDCl$_3$, 101 MHz) of compound **18**

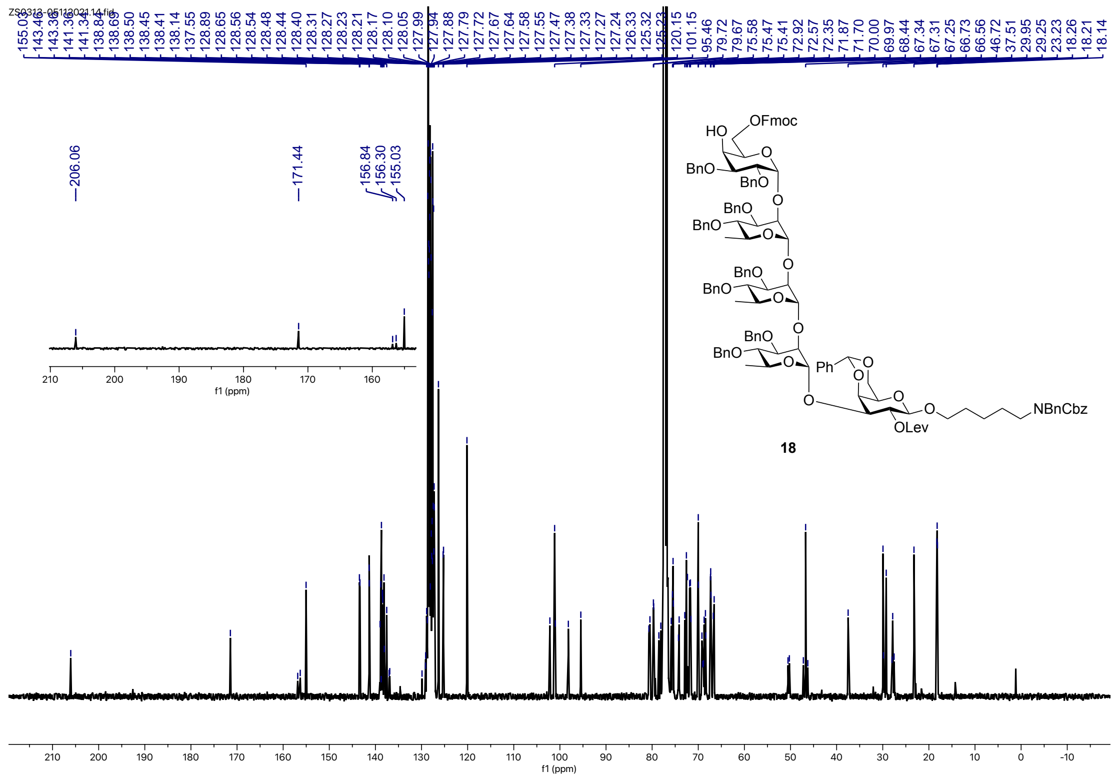

^{1}H-^{1}H COSY (CDCl$_3$, 400 MHz) of compound **18**

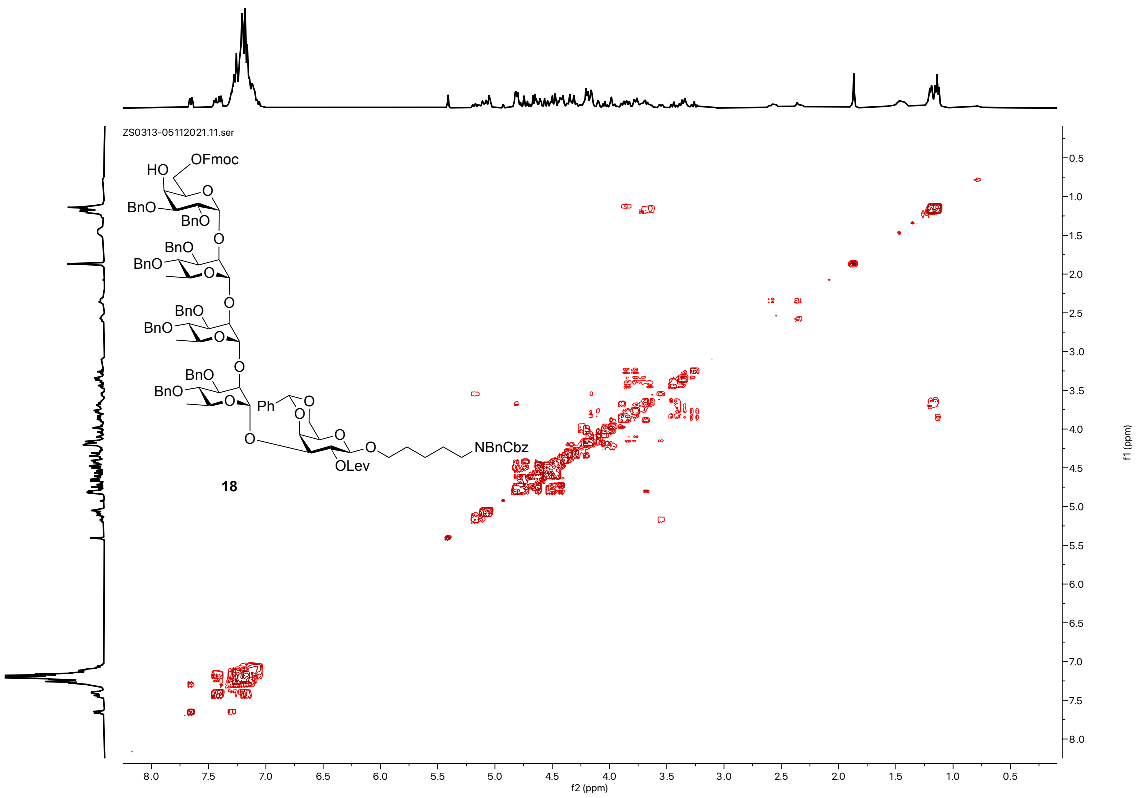

^{1}H-^{13}C HSQC (Decoupling, $CDCl_3$, 400 MHz) of compound **18**

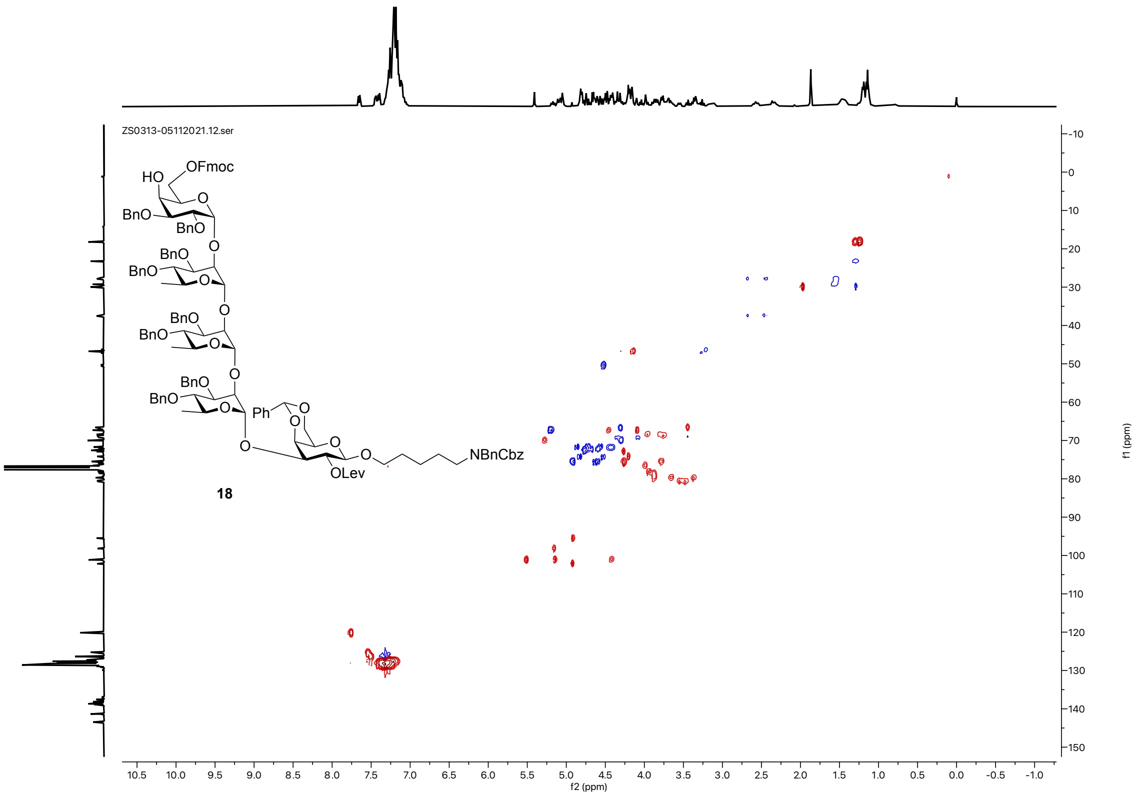

^{1}H-^{13}C HSQC (No decoupling, $CDCl_3$, 400 MHz) of compound **18**

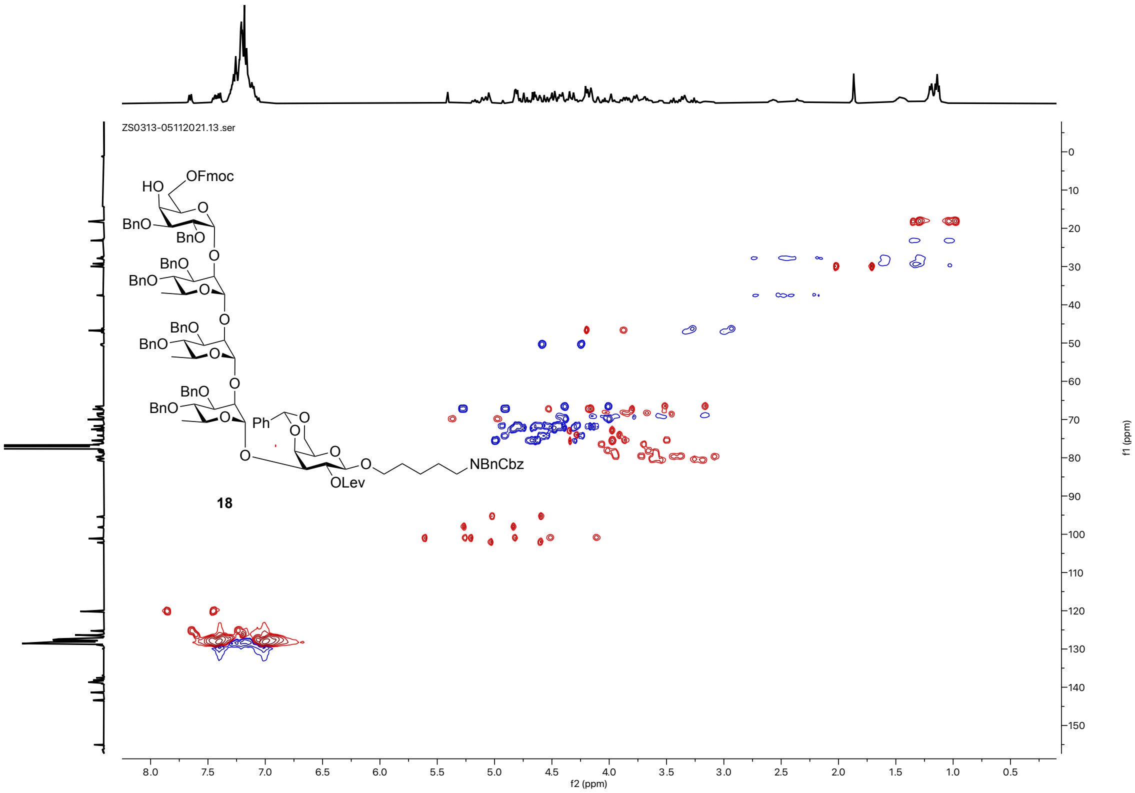

^{1}H NMR ($CDCl_3$, 700 MHz) of compound **19**

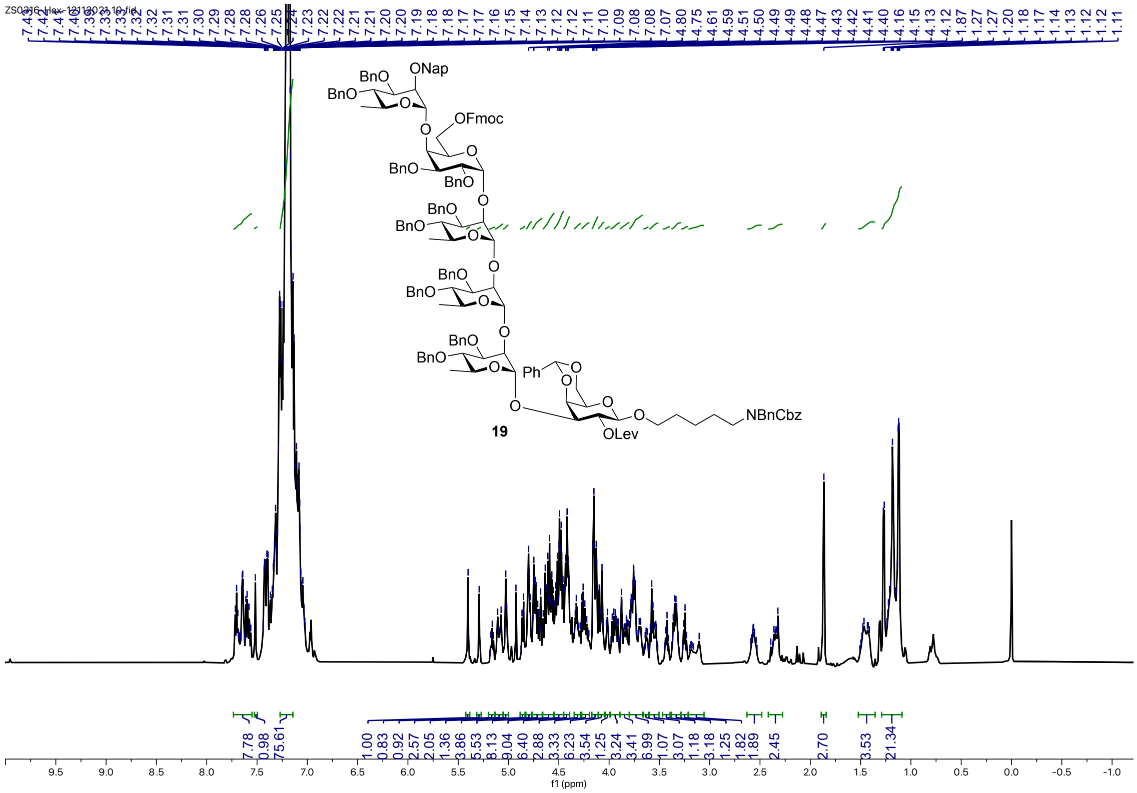

^{13}C NMR ($CDCl_3$, 176 MHz) of compound **19**

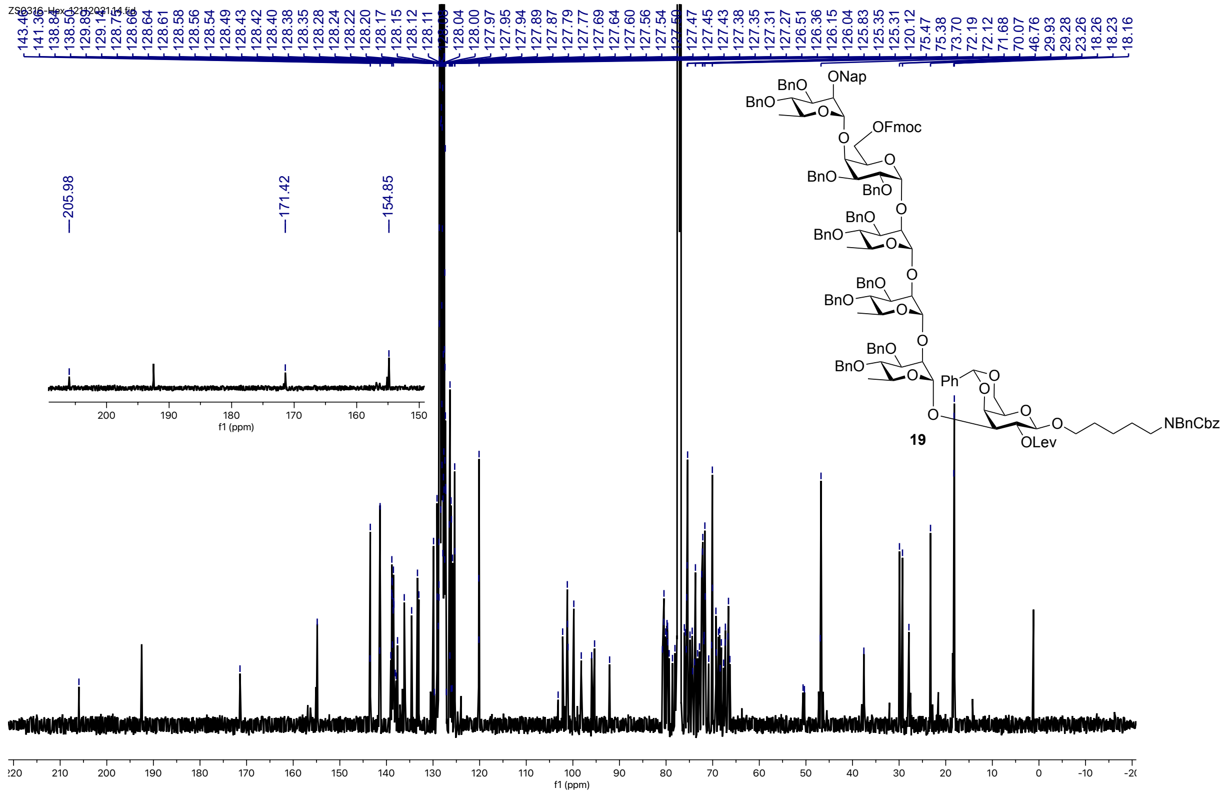

^{1}H-^{1}H COSY ($CDCl_3$, 700 MHz) of compound **19**

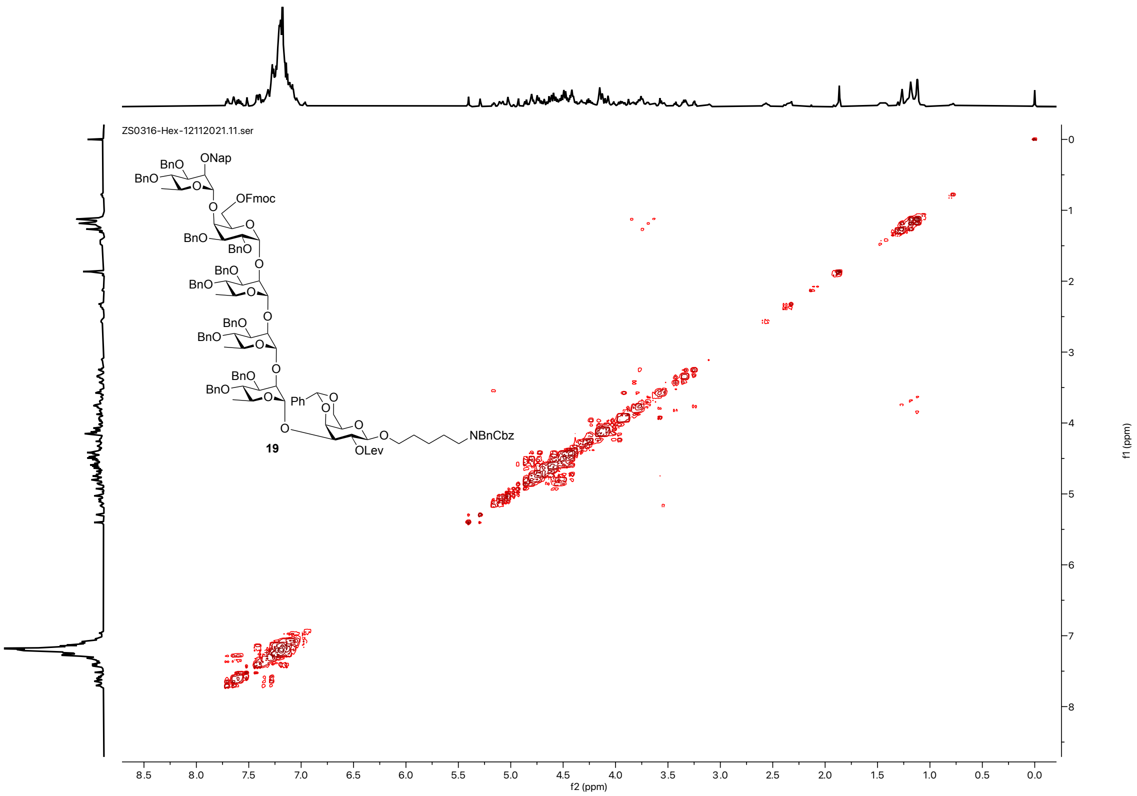

^{1}H-^{13}C HSQC (Decoupling, $CDCl_3$, 700 MHz) of compound **19**

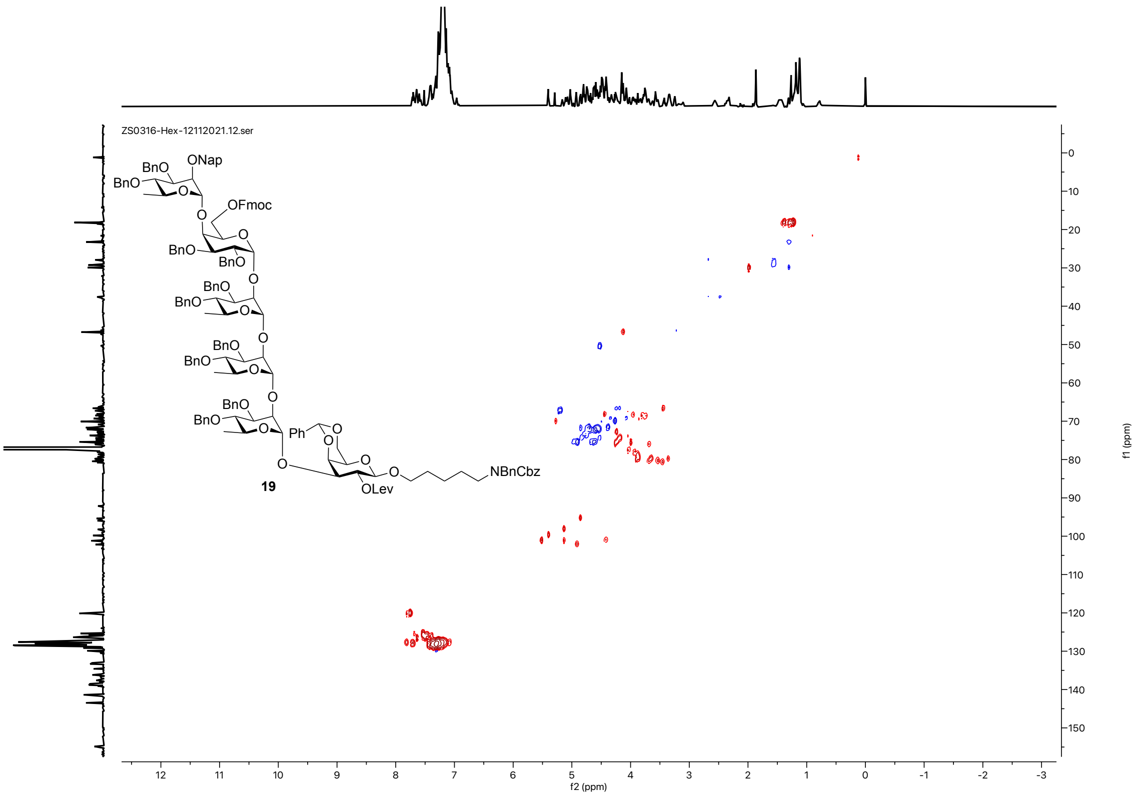

^{1}H-^{13}C HSQC (No decoupling, $CDCl_3$, 700 MHz) of compound **19**

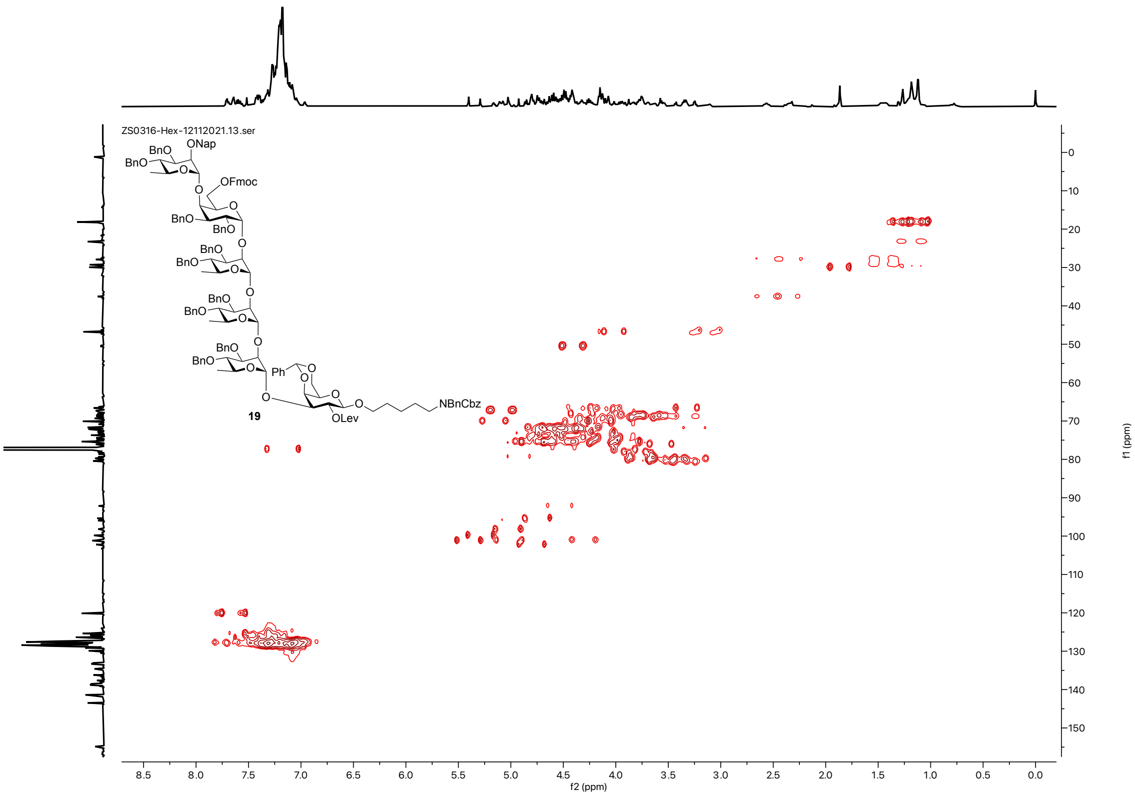

^{1}H NMR (D_2O, 600 MHz) of compound **20**

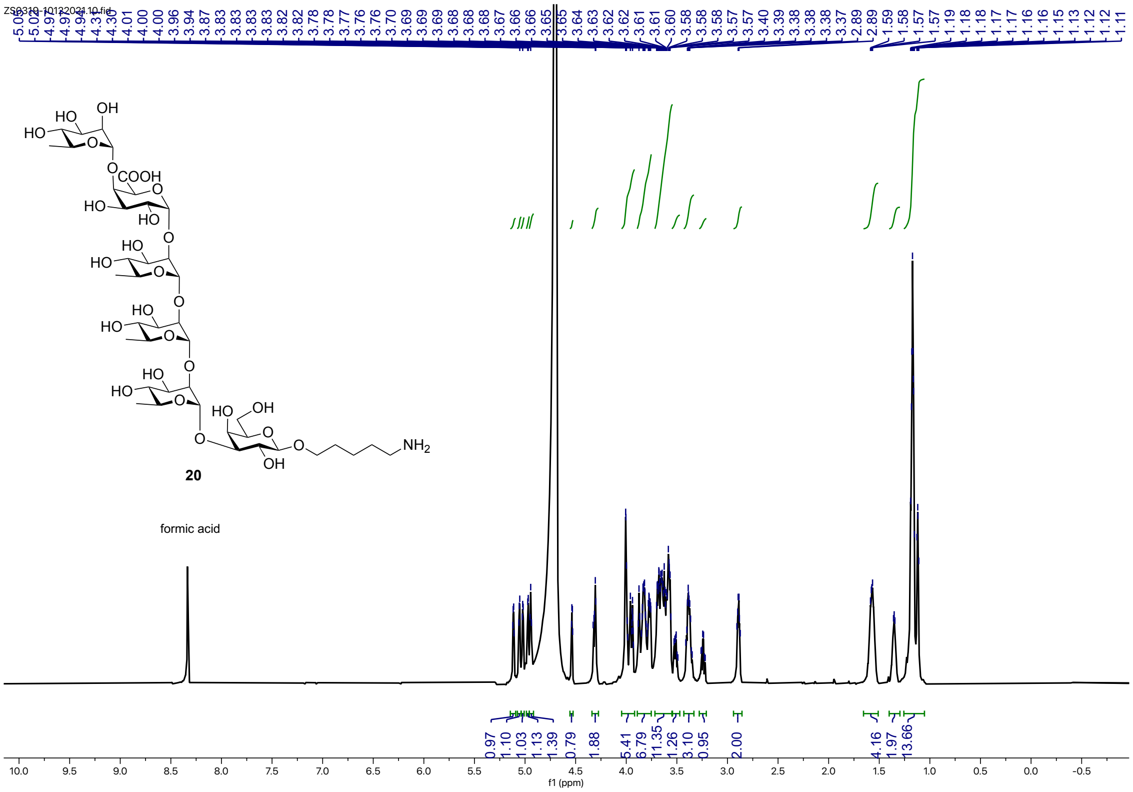

^{13}C NMR (D_2O, 151 MHz) of compound **20**

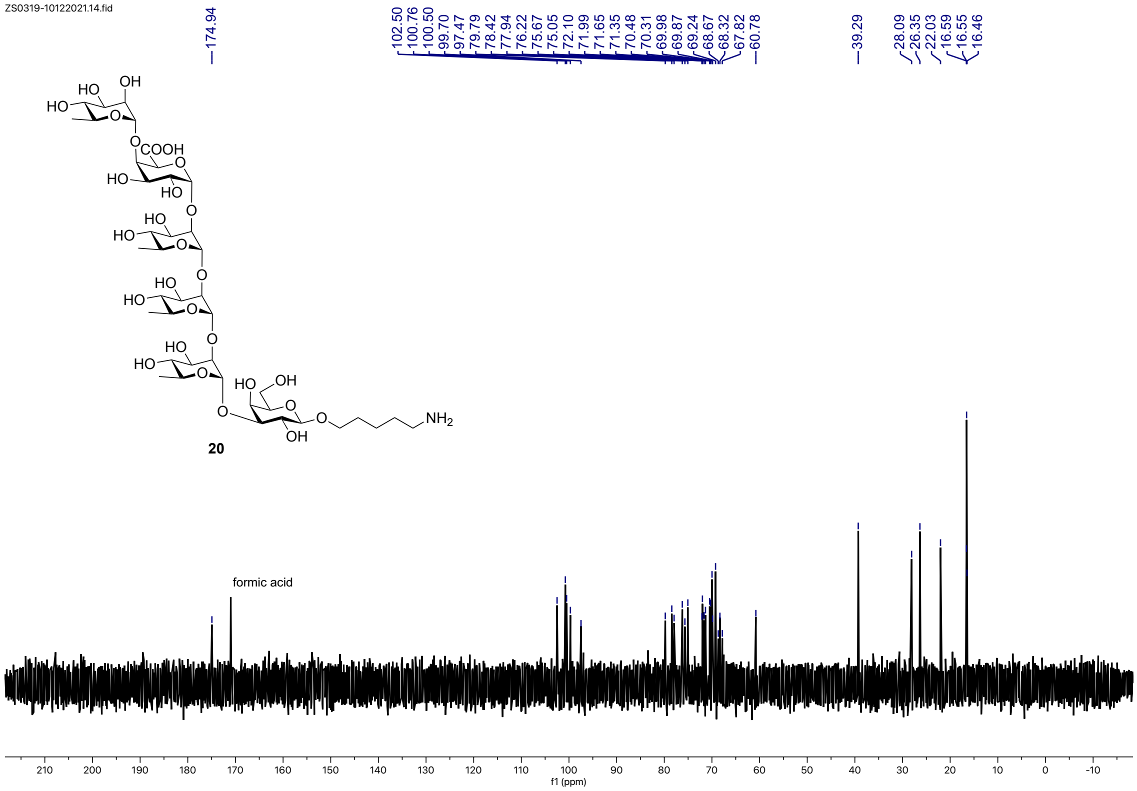

^{1}H-^{1}H COSY (D_2O, 600 MHz) of compound **20**

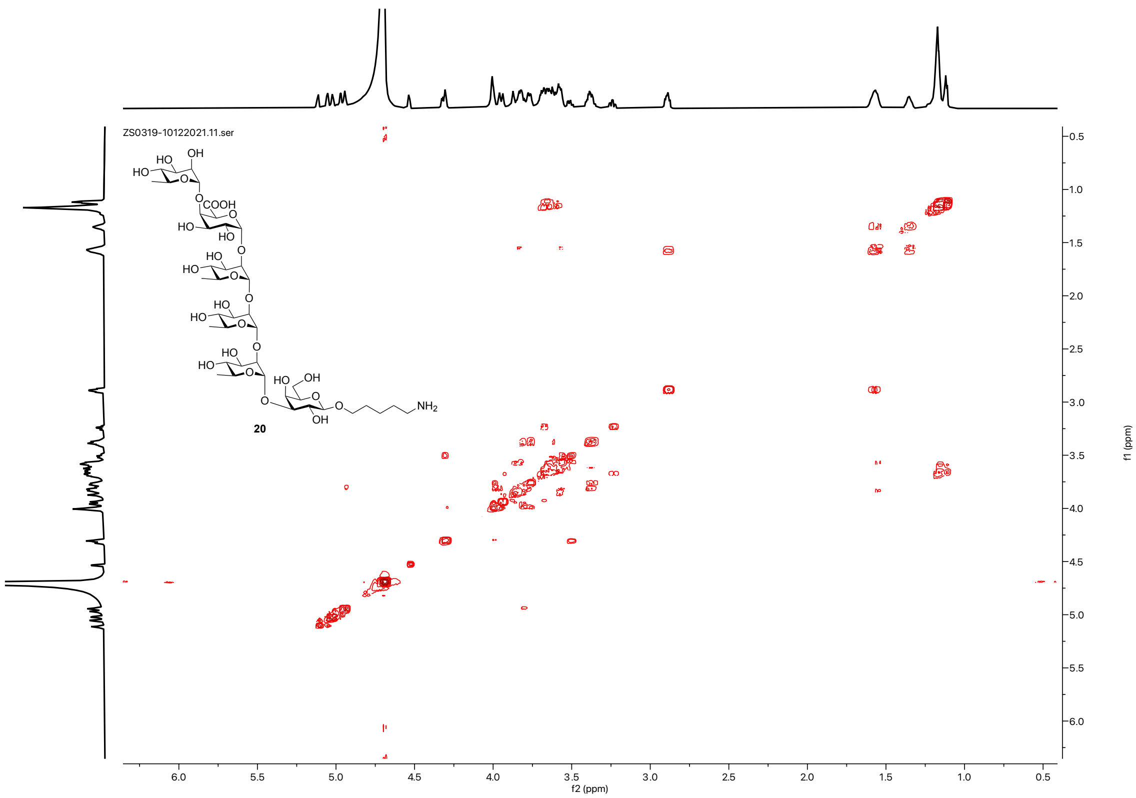

^{1}H-^{13}C HSQC (Decoupling, D_2O, 600 MHz) of compound **20**

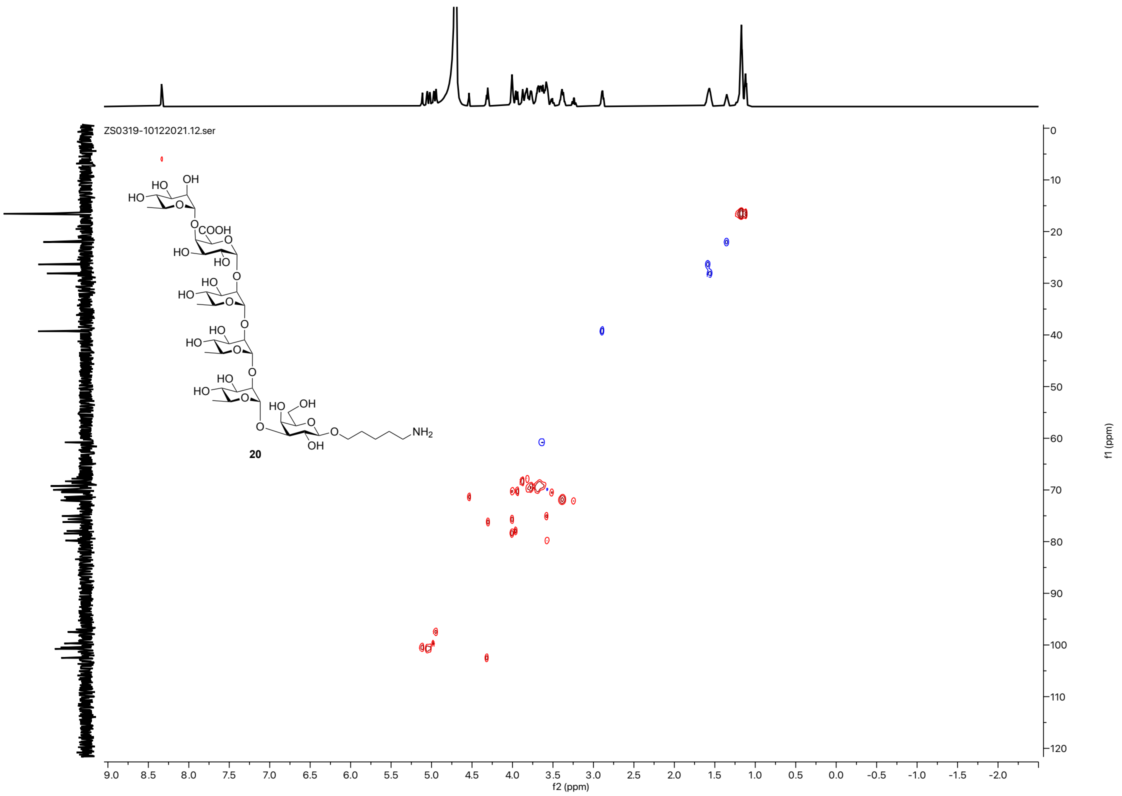

^{1}H-^{13}C HSQC (No decoupling, D_2O, 600 MHz) of compound **20**

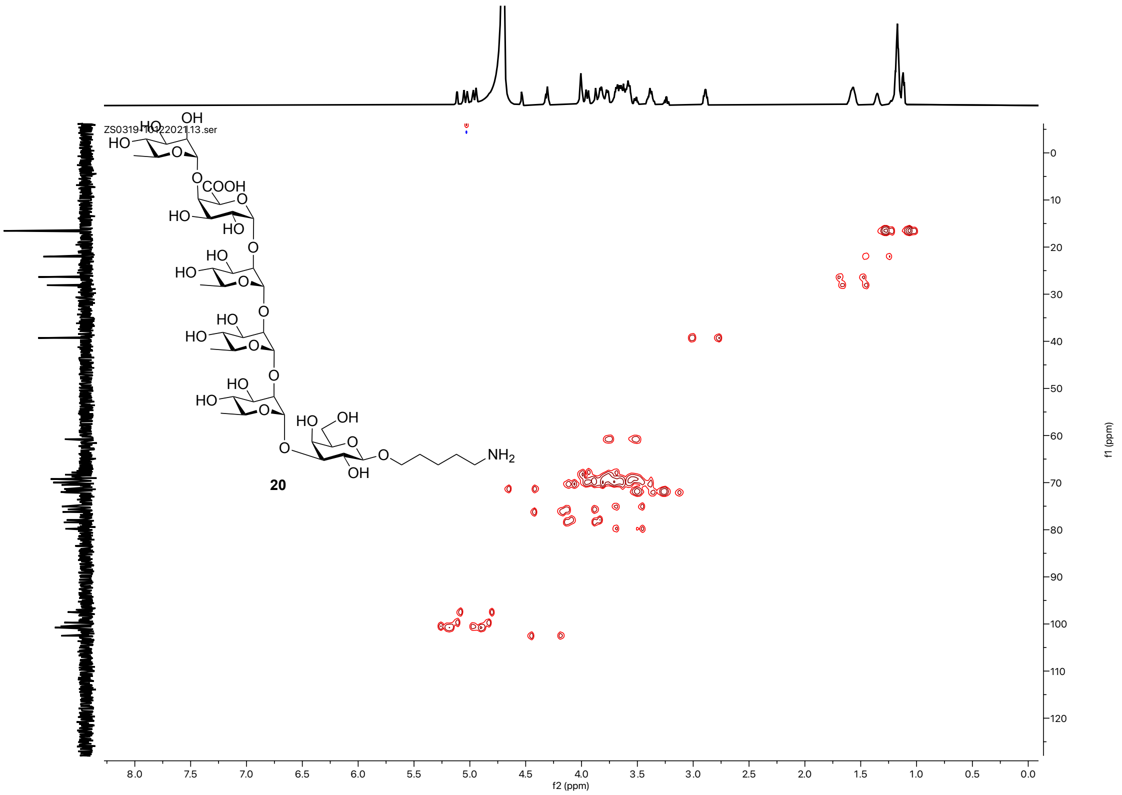

^{1}H NMR (D_2O, 600 MHz) of compound **21**

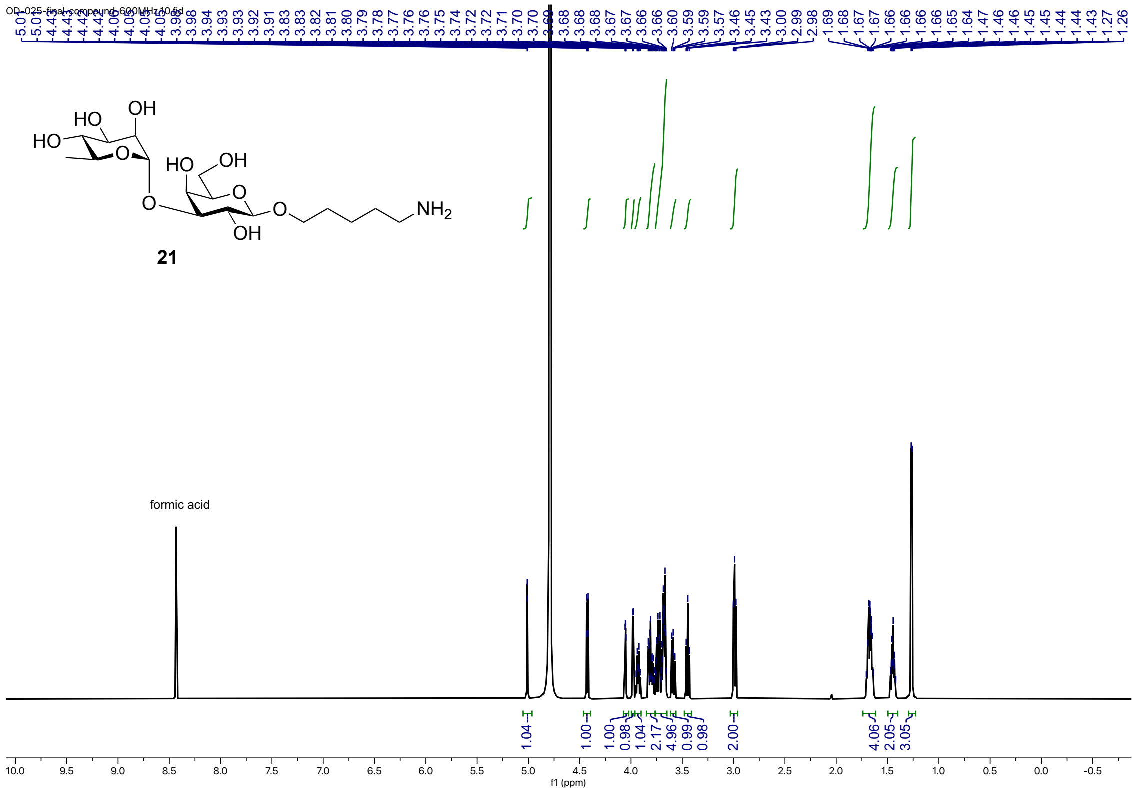

^{13}C NMR (D_2O, 151 MHz) of compound **21**

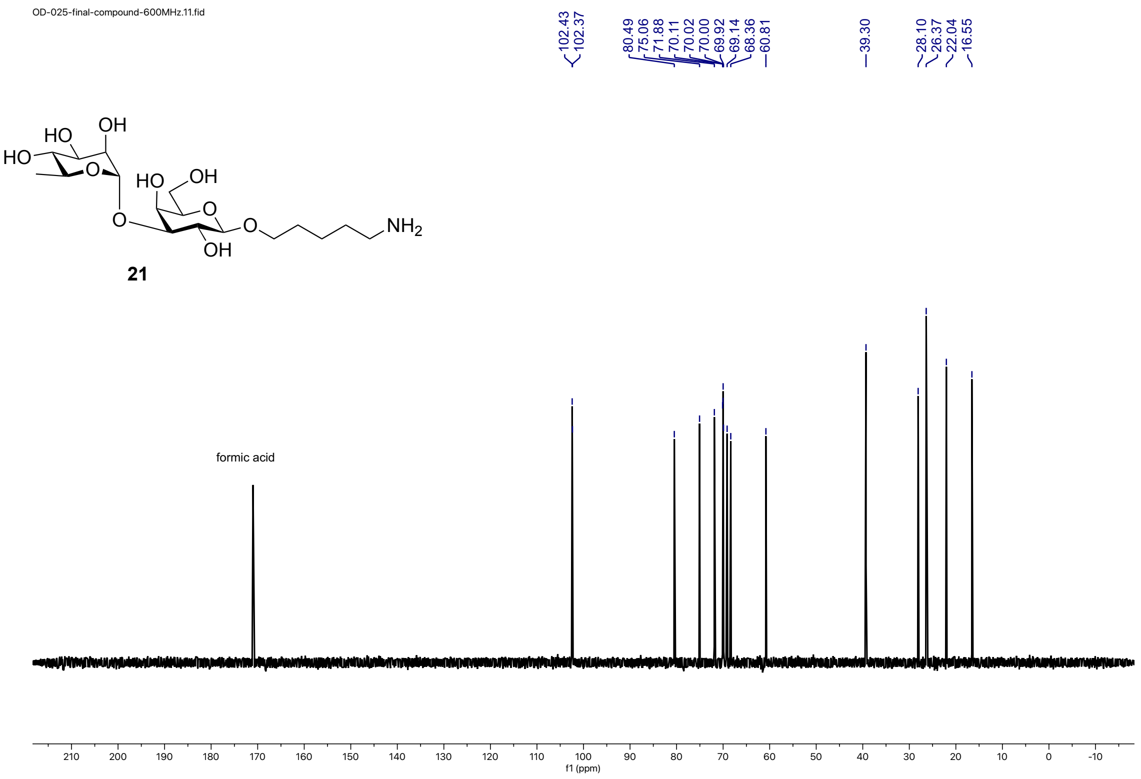

^1H-^1H COSY (D_2O, 600 MHz) of compound **21**

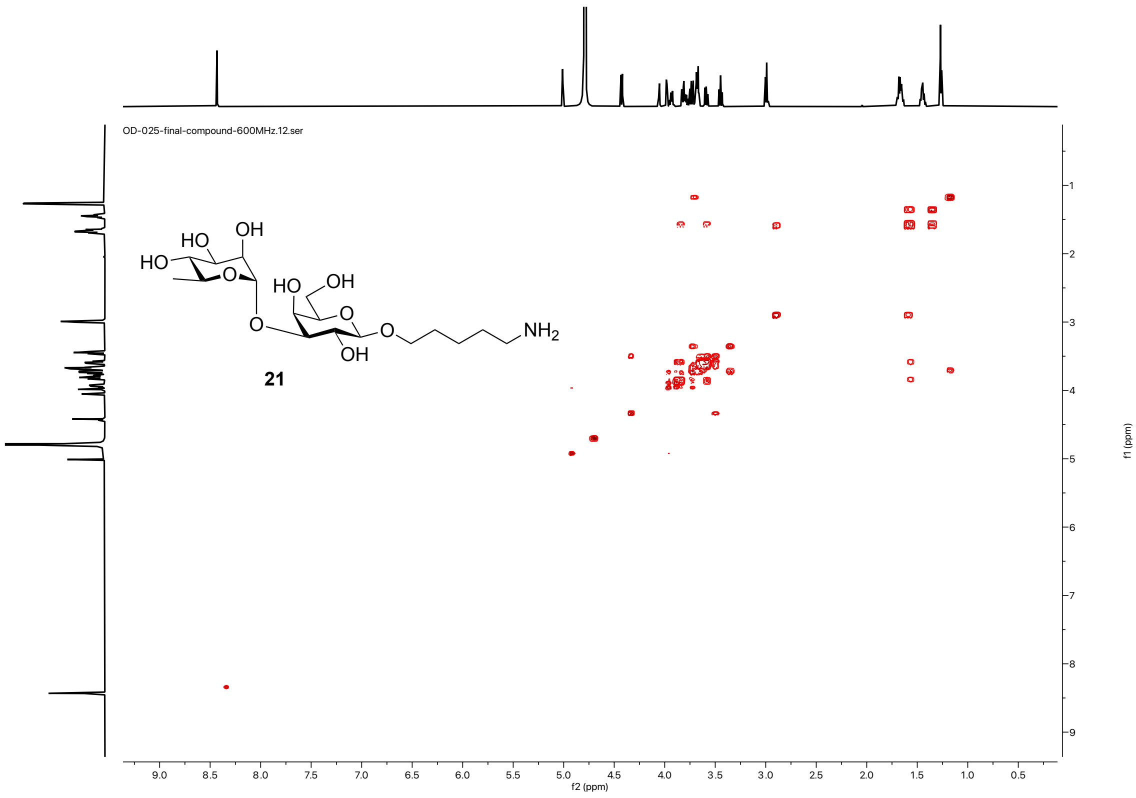

^1H-^{13}C HSQC (No decoupling, D_2O, 600 MHz) of compound **21**

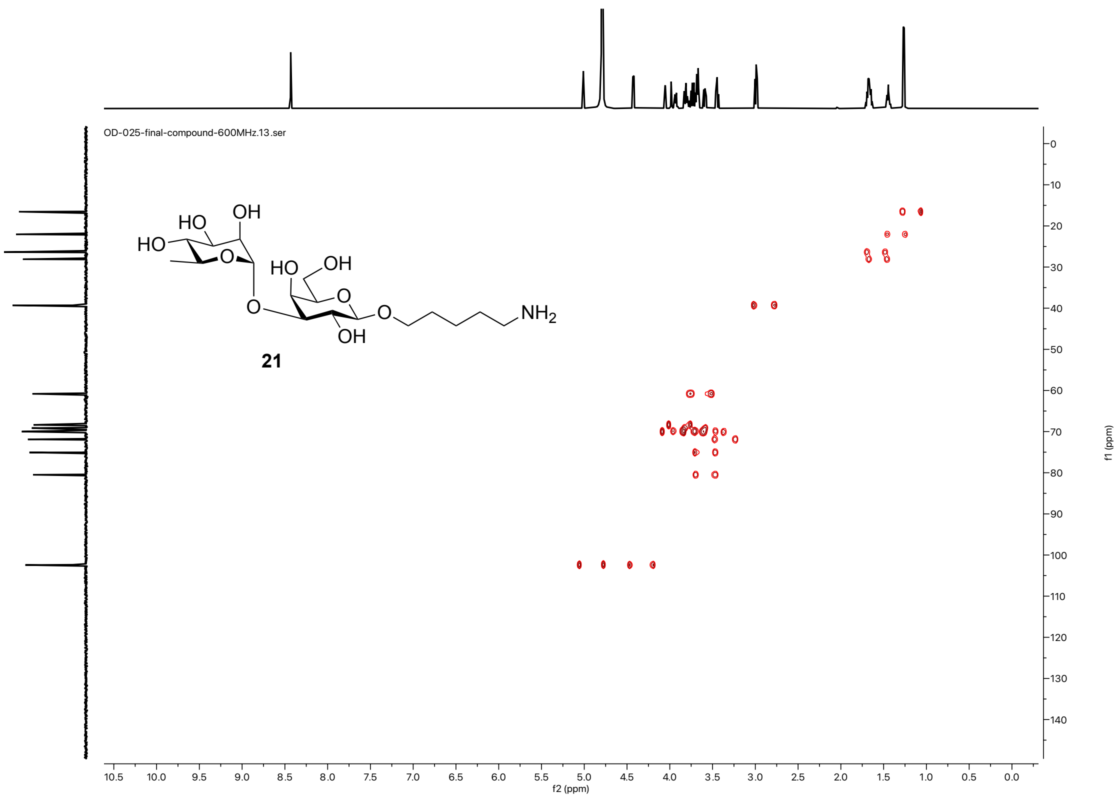

^{1}H NMR (D_2O, 700 MHz) of compound **22**

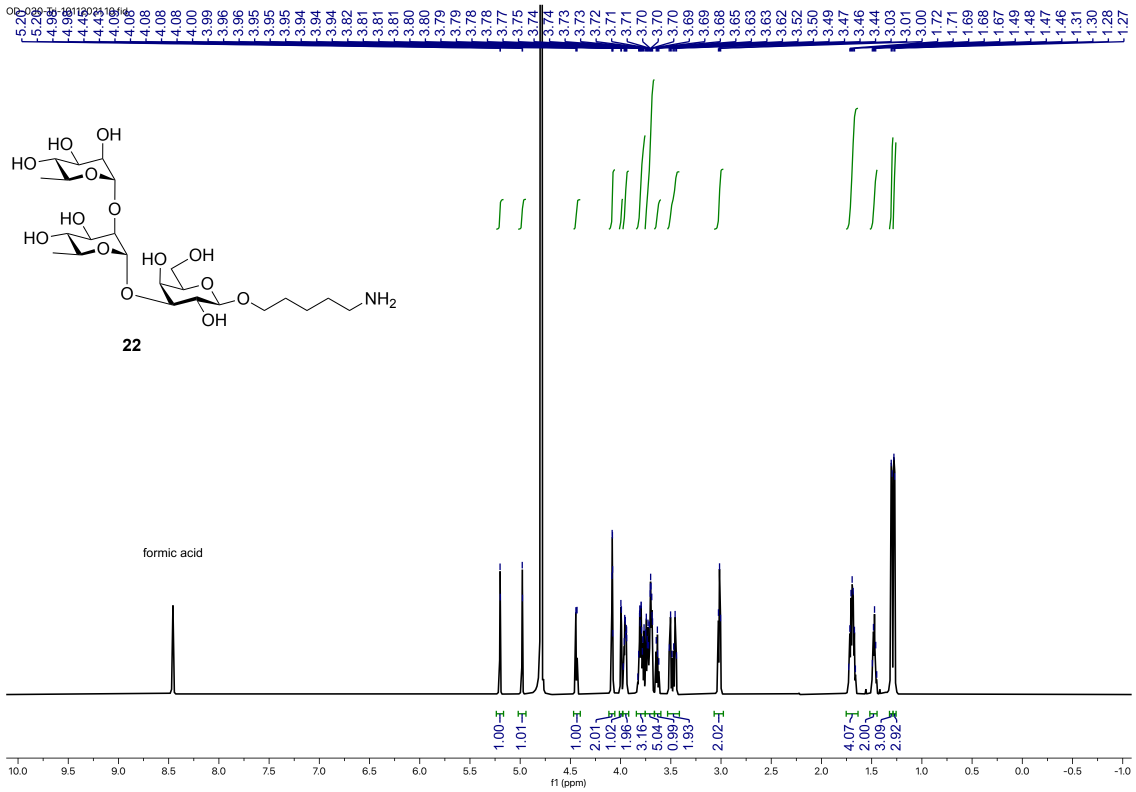

^{13}C NMR (D_2O, 176 MHz) of compound **22**

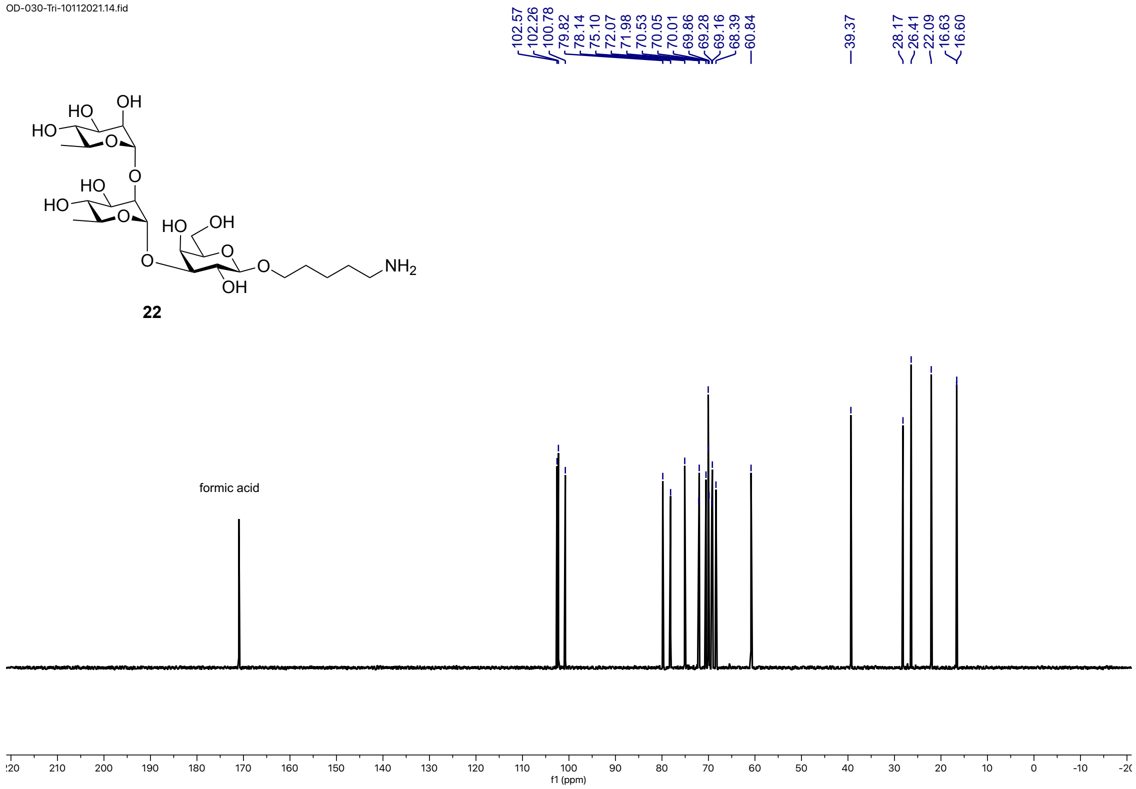

^{1}H-^{1}H COSY (D_2O, 700 MHz) of compound **22**

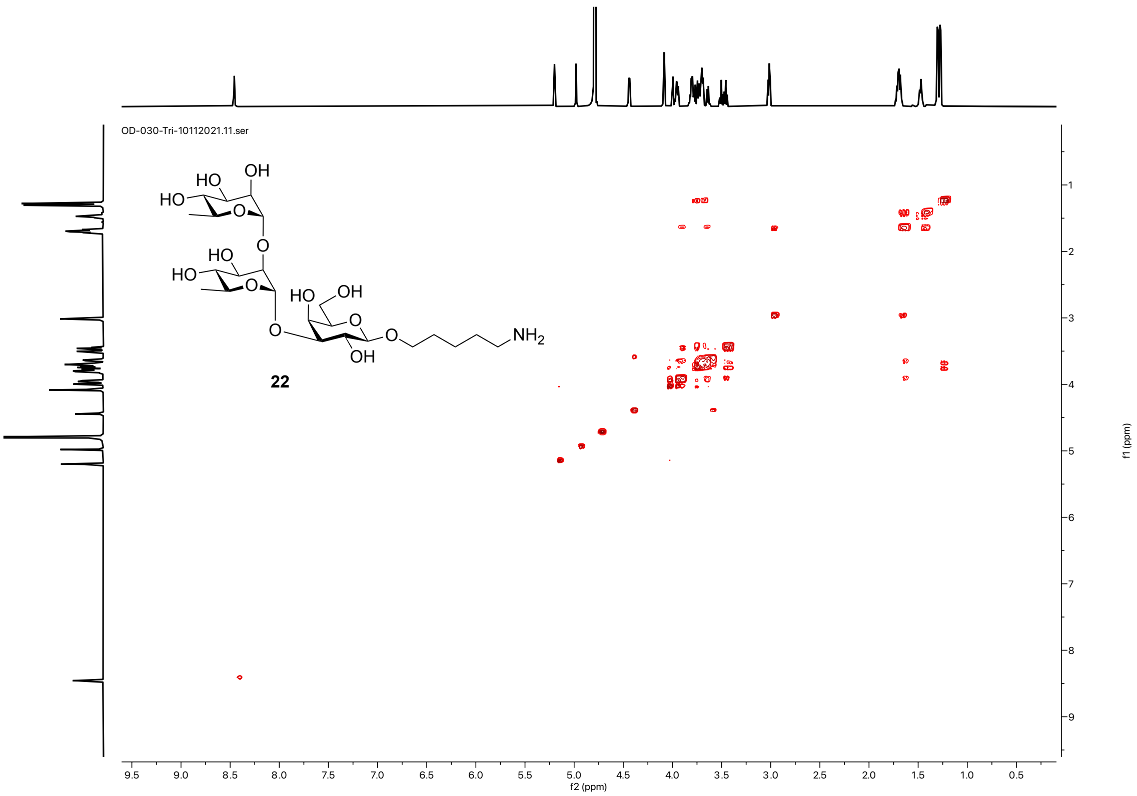

^{1}H-^{13}C HSQC (Decoupling, D_2O, 700 MHz) of compound **22**

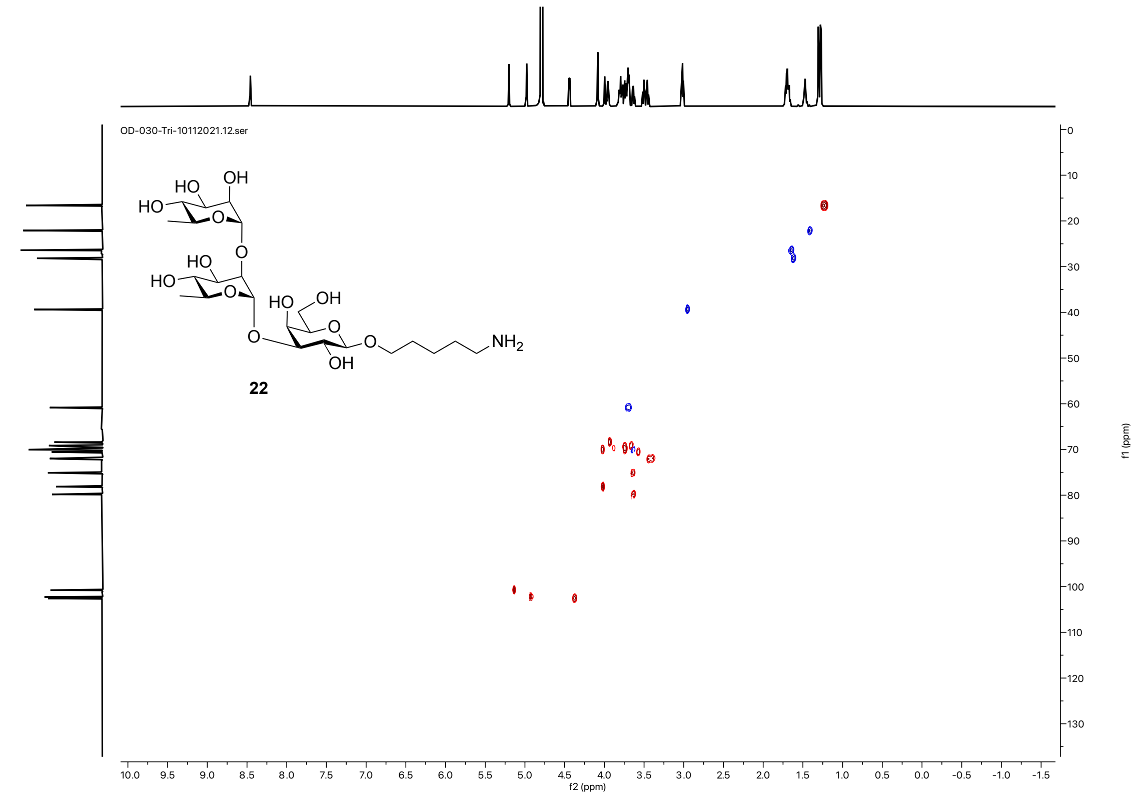

^{1}H-^{13}C HSQC (No decoupling, D_2O, 700 MHz) of compound **22**

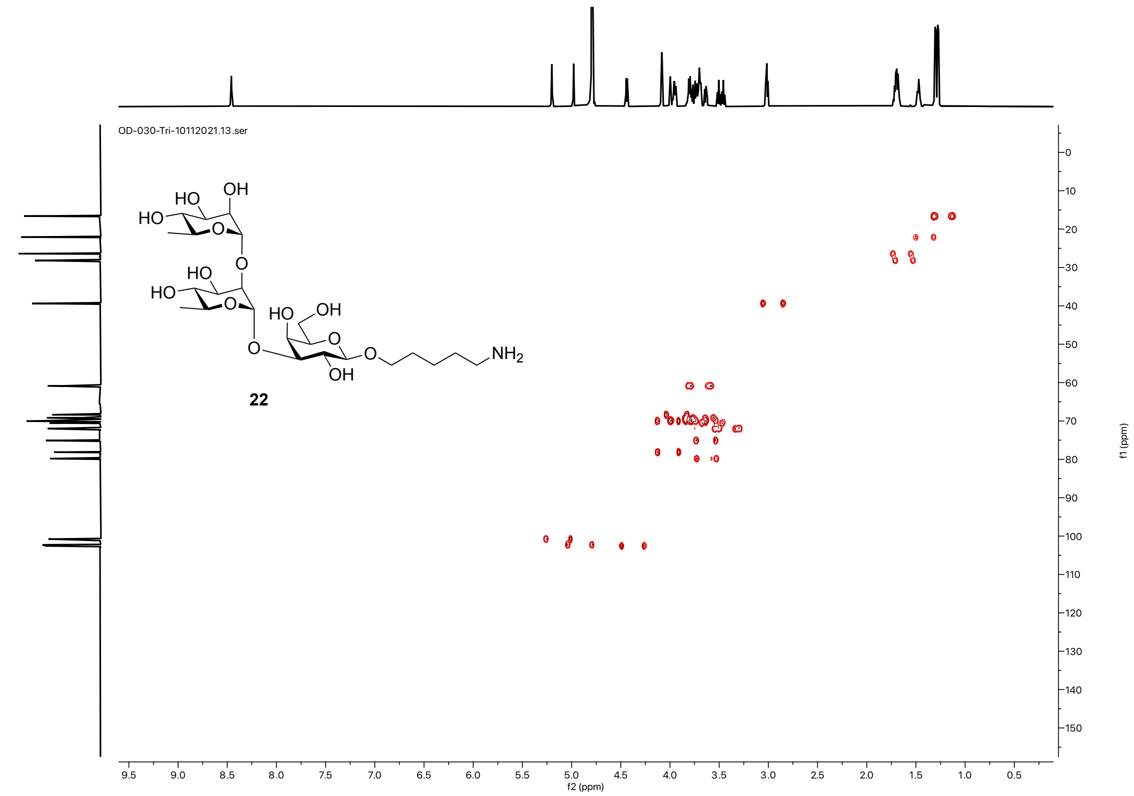

^{1}H NMR (D_2O, 600 MHz) of compound **23**

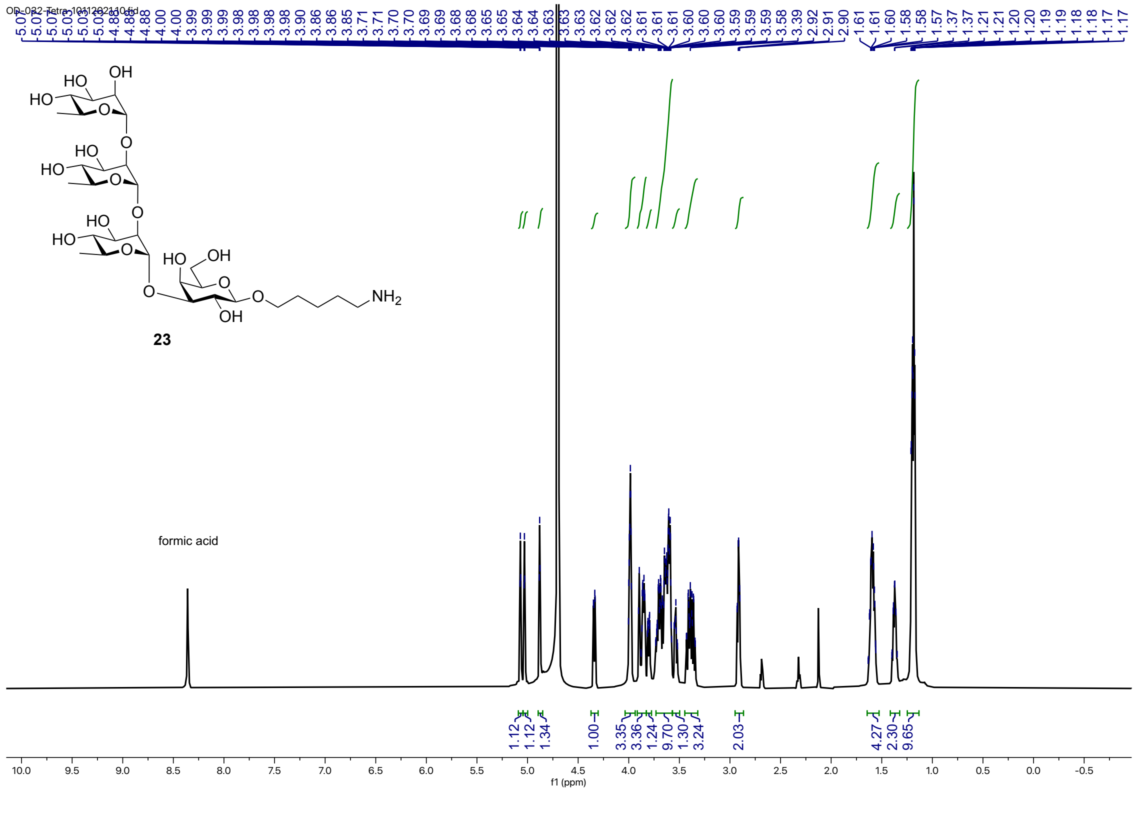

^{13}C NMR (D_2O, 151 MHz) of compound **23**

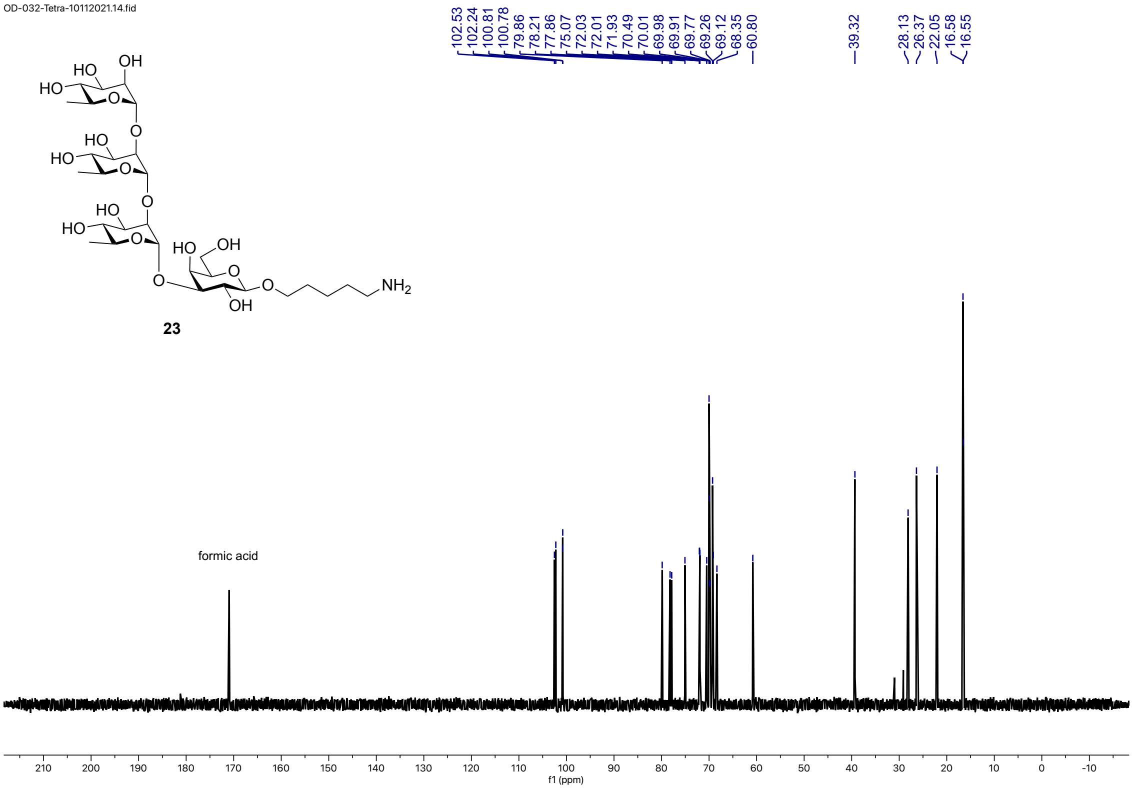

^{1}H-^{1}H COSY (D_2O, 600 MHz) of compound **23**

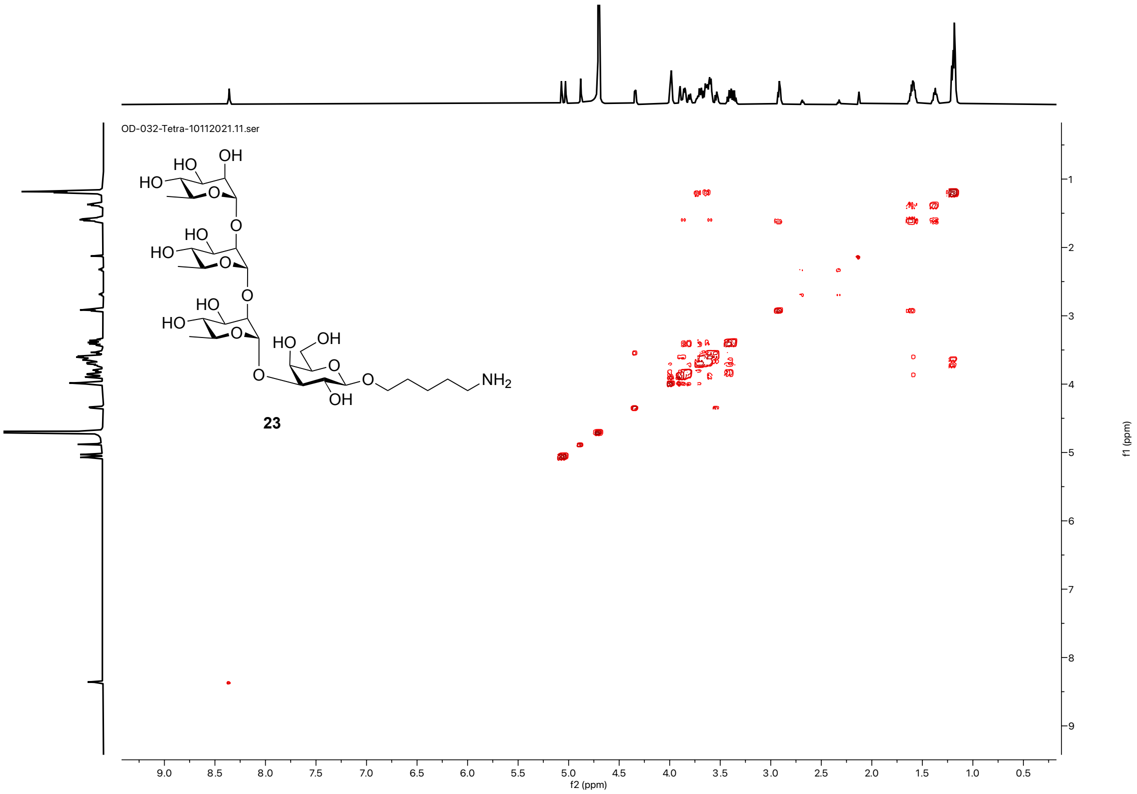

^{1}H-^{13}C HSQC (Decoupling, D_2O, 600 MHz) of compound **23**

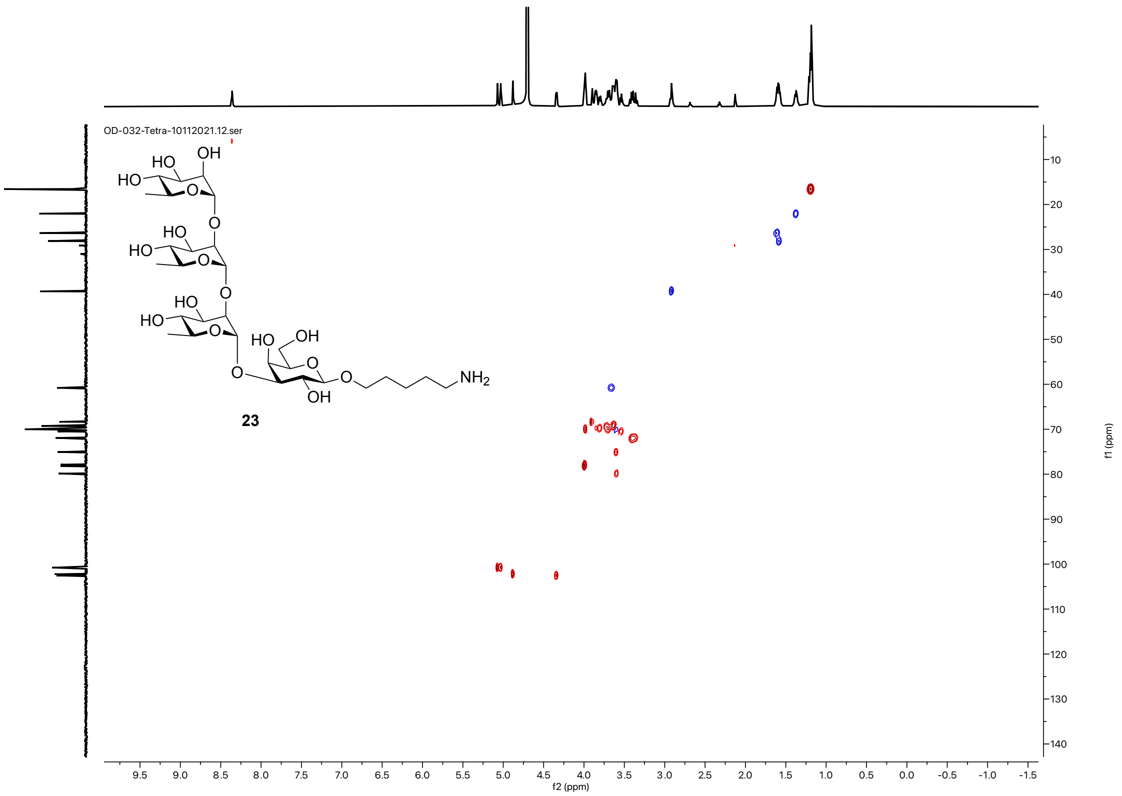

^{1}H-^{13}C HSQC (No decoupling, D_2O, 600 MHz) of compound **23**

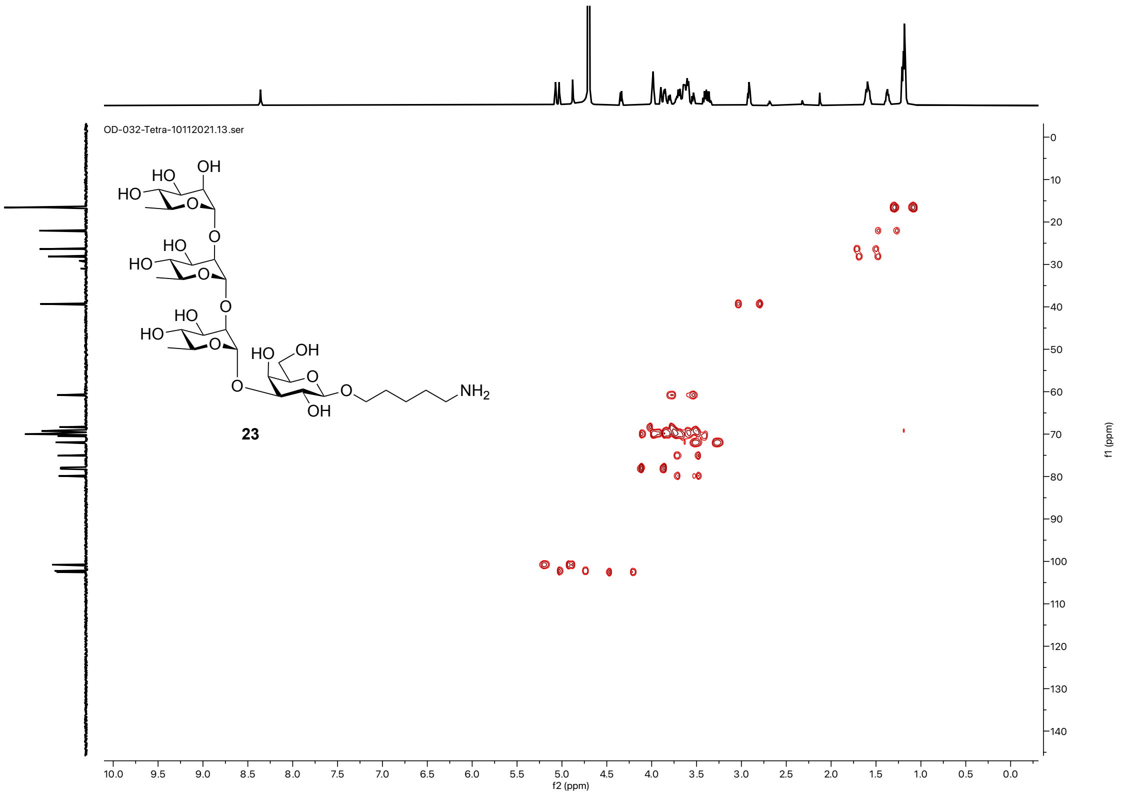

^1H NMR (D_2O, 600 MHz) of compound **24**

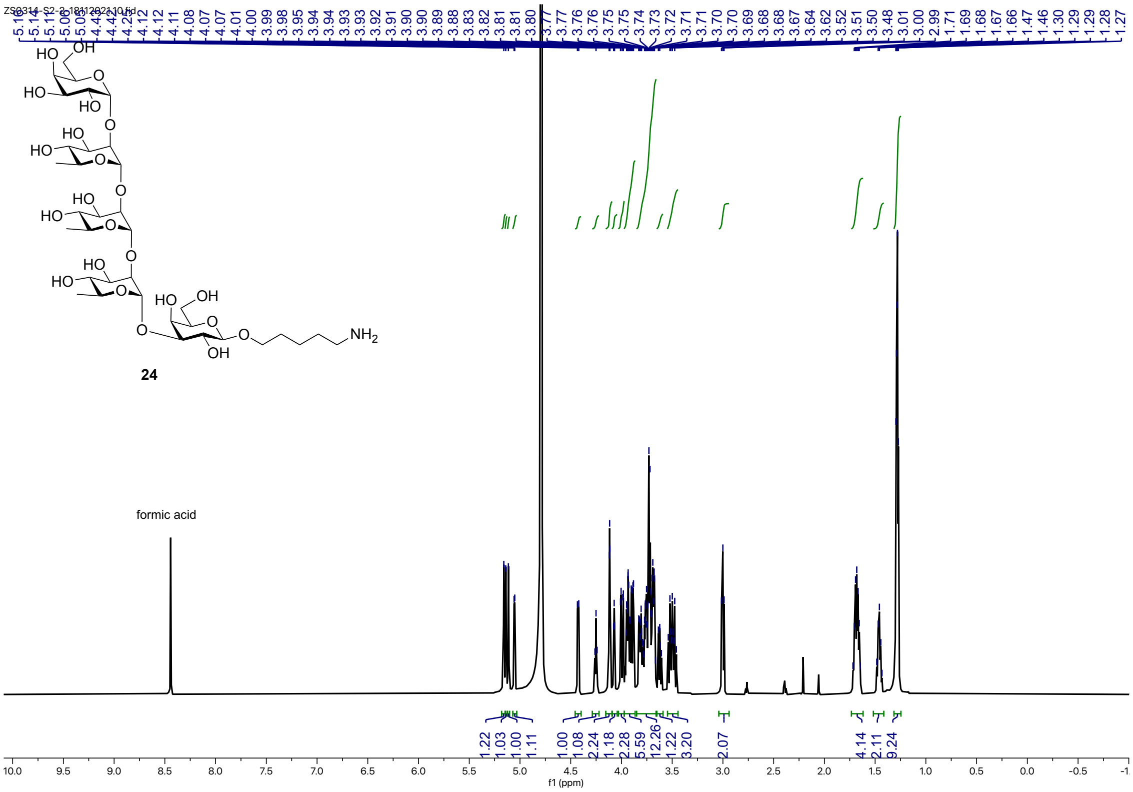

^{13}C NMR (D_2O, 151 MHz) of compound **24**

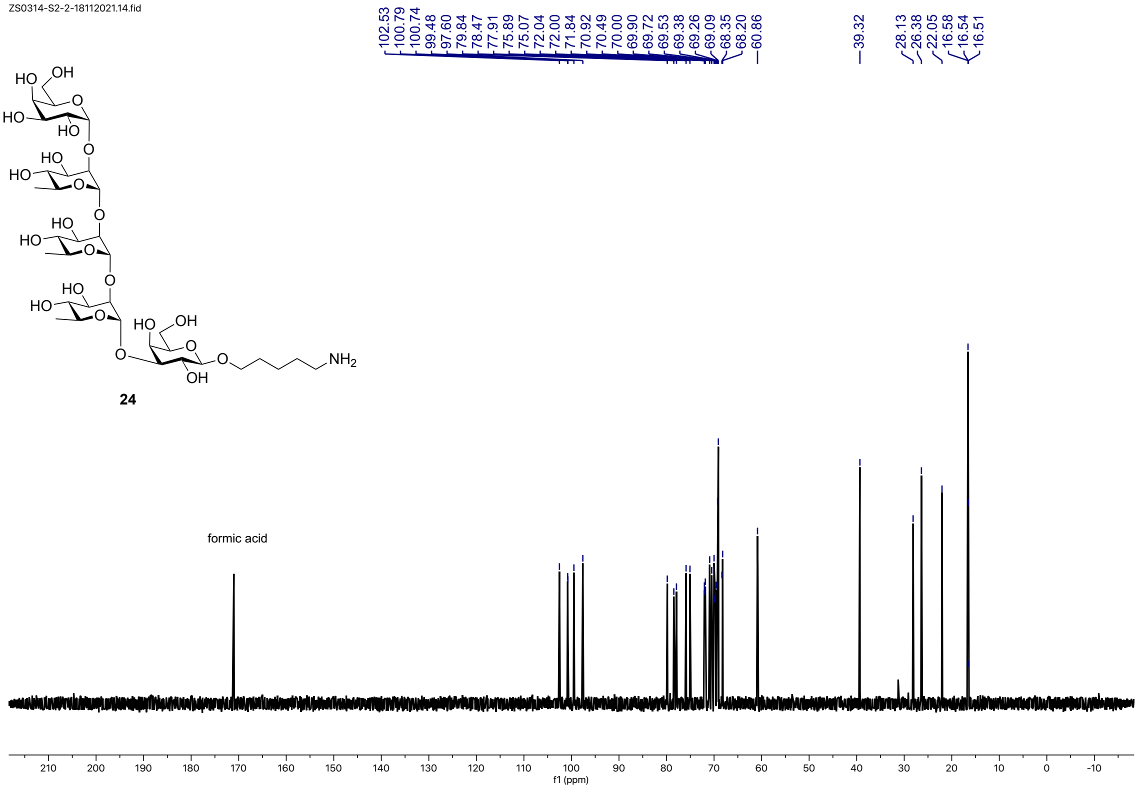

^1H-^1H COSY (D_2O, 600 MHz) of compound **24**

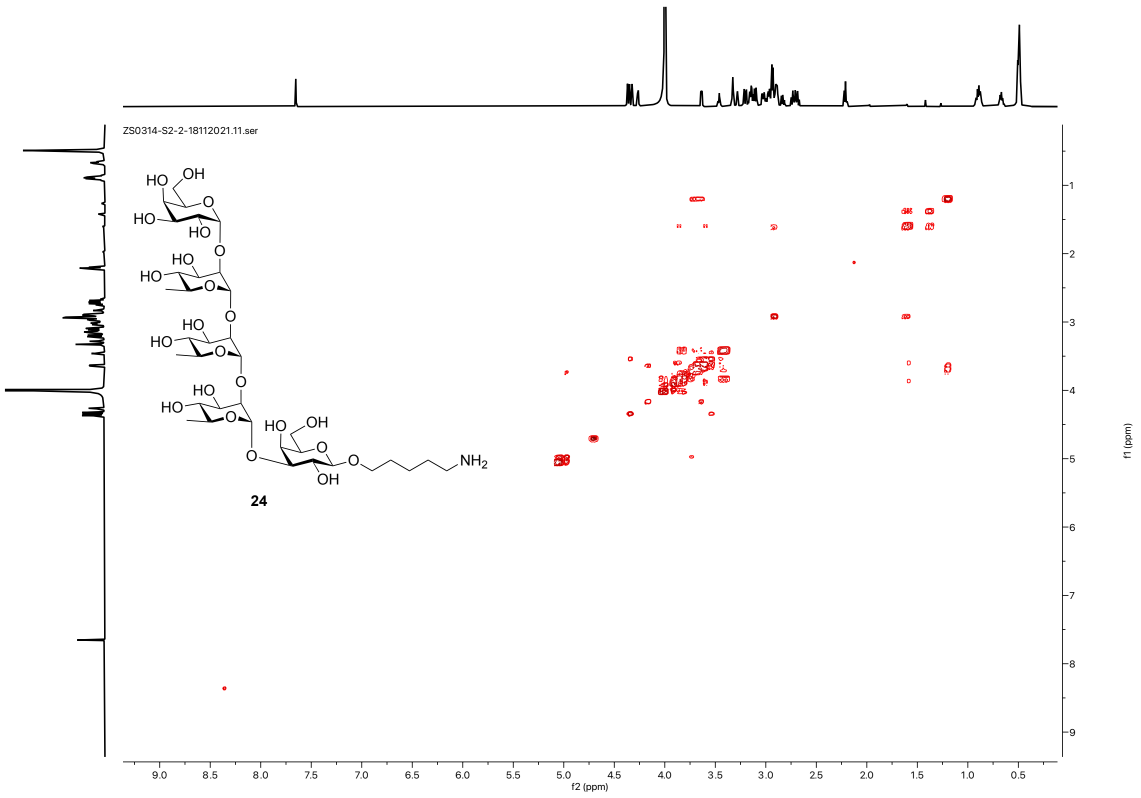

^1H-^{13}C HSQC (Decoupling, D_2O, 600 MHz) of compound **24**

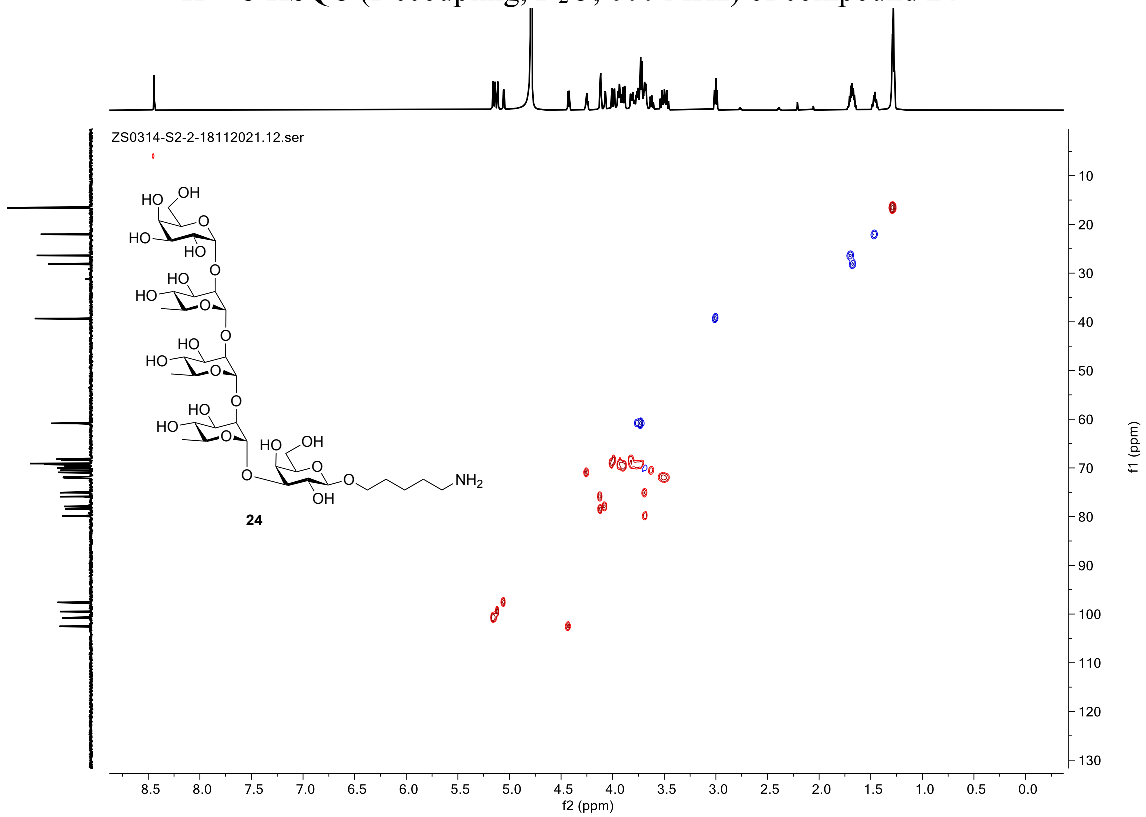

^1H-^{13}C HSQC (No decoupling, D_2O, 600 MHz) of compound **24**

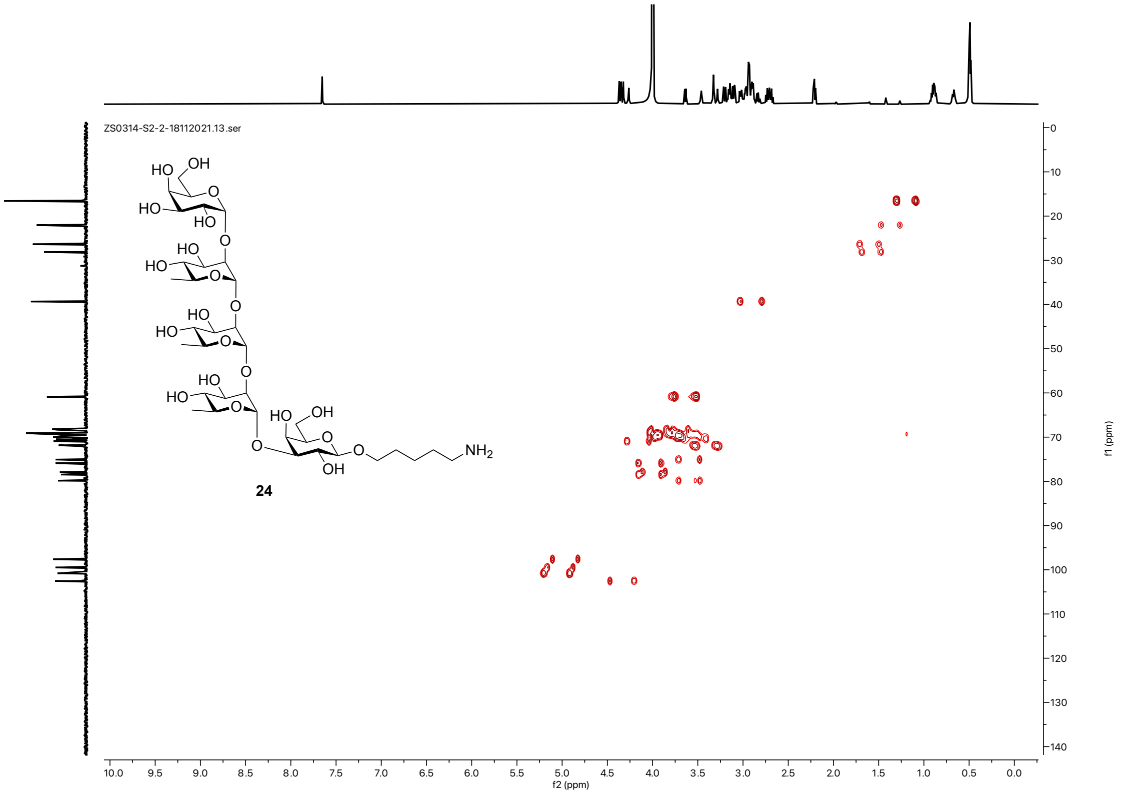

4. References

(1) Crich, D.; Vinogradova, O. Synthesis of the Antigenic Tetrasaccharide Side Chain from the Major Glycoprotein of *Bacillus Anthracis* Exosporium. *J. Org. Chem.* **2007**, *72* (17), 6513–6520. https://doi.org/10.1021/jo070750s.

(2) Zhang, S.; Sella, M.; Sianturi, J.; Priegue, P.; Shen, D.; Seeberger, P. H. Discovery of Oligosaccharide Antigens for Semi-Synthetic Glycoconjugate Vaccine Leads against Streptococcus Suis Serotypes 2, 3, 9 and 14. *Angewandte Chemie International Edition* **2021**, *60* (26), 14679–14692. https://doi.org/10.1002/anie.202103990.

(3) Castelli, R.; Overkleeft, H. S.; Van Der Marel, G. A.; Codée, J. D. C. 2,2-Dimethyl-4-(4-Methoxy-Phenoxy) Butanoate and 2,2-Dimethyl-4-Azido Butanoate: Two New Pivaloate-Ester-like Protecting Groups. *Org. Lett.* **2013**, *15* (9), 2270–2273. https://doi.org/10.1021/ol4008475.

(4) Lin, S.; Lowary, T. L. Synthesis of the Highly Branched Hexasaccharide Core of Chlorella Virus *N* -Linked Glycans. *Chemistry A European J* **2018**, *24* (64), 16992–16996. https://doi.org/10.1002/chem.201804795.

(5) Agnihotri, G.; Tiwari, P.; Misra, A. K. One-Pot Synthesis of per-O-Acetylated Thioglycosides from Unprotected Reducing Sugars. *Carbohydrate Research* **2005**, *340* (7), 1393–1396. https://doi.org/10.1016/j.carres.2005.02.027.
